# Cumulative evidence for associations between genetic variants in interleukin 17 family gene and risk of human diseases

**DOI:** 10.3389/fimmu.2022.1008184

**Published:** 2022-10-10

**Authors:** Tianyu Liu, Lei Yang, Xiaolong Lv, Chunjian Zuo, Chenhao Jia, Zelin Yang, Chongqi Fan, Huanwen Chen

**Affiliations:** ^1^ Department of Cardiothoracic Surgery, The First Affiliated Hospital of Chongqing Medical University, Chongqing, China; ^2^ Department of Thoracic Surgery, Army Medical Center of People’s Liberation Army of China (PLA), Chongqing, China

**Keywords:** interleukin 17 family gene, variant, cancer, noncancerous diseases, susceptibility

## Abstract

**Background:**

Genetic association studies have elucidated the link of variants in the interleukin 17 (*IL-17*) family genes with susceptibility to human diseases, yet have obtained controversial outcomes. Therefore, we sought to update comprehensive synopsis of variants in the *IL-17* family genes with susceptibility to human diseases.

**Methods:**

Our study screened the Pubmed and Web of Science to enroll eligible articles and performed a meta-analysis, then graded the cumulative evidence of significant association using Venice criteria and false-positive report probability test, and finally assessed the function of variants with strong evidence.

**Results:**

Seven variants in *IL-17* family genes had significant relationships with susceptibility to 18 human diseases identified by meta-analyses. Strong evidence was assigned to 4 variants (*IL-17A* rs2275913, *IL-17A* rs8193037, *IL-17F* rs1889570, *IL-17F* rs763780) with susceptibility to 6 human diseases (lung and cervical cancer, spondyloarthritis, asthma, multiple sclerosis, rheumatoid arthritis), moderate to 2 variants with risk of 5 diseases, weak to 5 variants with risk of 10 diseases. Bioinformatics analysis suggested that the variants with strong evidence might fall in putative functional regions. Additionally, positive relationships for 5 variants with risk of 4 diseases (based on two datasets) and 14 variants with risk of 21 diseases (based on one dataset) were considered noteworthy.

**Conclusions:**

This study offers updated and comprehensive clues that variants in the *IL-17* family genes are significantly linked with susceptibility to cervical, lung cancer, asthma, multiple sclerosis, rheumatoid arthritis and spondyloarthritis, and elucidates the crucial role of the *IL-17* regions in the genetic predisposition to cancer or noncancerous diseases.

## Introduction

Interleukin (IL) 17 (*IL-17*), a homodimeric glycoprotein composed of 155 amino acids, remains a pro-inflammatory and its family genes contain six groups (*IL-17A* to *F*) (1). The *IL-17* signaling system has a crucial impact on different tissues such as lung, skin, kidney, brain, bone, articular cartilage, meniscus and hematopoietic tissue (1); this system mediated by the binding to *IL-17* receptors can active multiple cell types (such as fibroblasts, endothelial cells, epithelial cells, keratinocytes and macrophages) (2). It could be activated which produces cell subsets of *IL-17* that play a crucial role in multiple essential biological activities and accelerating occurrences of human diseases, involving novel coronavirus disease 2019 (COVID-19) (3).

As early as 2006, Hizawa et al. found five single nucleotide polymorphisms (SNPs) in *IL-17F* and found that rs763780 {His-to-Arg substitution at amino acid 161 (H161R)} in the third exon of the *IL-17F* gene influenced the susceptibility to asthma and chronic obstructive pulmonary disease (COPD) in the Japanese population (4, 5). Since then, a range of genetic association studies found that SNPs in *IL-17* family genes have been shown to be linked with the risk of multiple diseases. In 2007, Arisawa et al. identified that *IL-17F* rs763780 and *IL-17A* rs2275913 in Japanese population had been shown to be linked with the risk of ulcerative colitis (UC) (6). Subsequently, studies also found that *IL-17* family genes are linked with multiple cancers risk, including ovarian (7), breast (8), hepatocellular (9), esophageal (10), gastric (11) and lung cancer (12). In 2014, two researchers independently performed a meta-analysis and attempted to elucidate the relationship between *IL-17A* rs2275913 and *IL-17F* rs763780 and cancer risk in Asians (13, 14). Interestingly, the outcomes of the two studies were inconsistent. Recently, in an updated meta-analysis conducted in multiple countries from the Asian ancestry, SNP rs2275913 and SNP rs763780 associated with 31,234 subjects were tested. Then it was discovered that *IL-17A* rs2275913 acted as risk factors for gastric, cervical, colorectal and oral cancer, and *IL-17F* rs763780 for cervical and oral cancer (15).

Even though previous studies evaluate the relationship between SNPs in *IL-17* family genes and the risk of diseases, the outcomes are controversial. In addition, an updated research synopsis with comprehensive functional annotation had not been conducted to assess the epidemiological evidence of associations with *IL-17* family genes and risk of all human diseases thus far. Therefore, we carried out meta-analysis to elucidate the relationships of SNPs in the *IL-17* genes with susceptibility to disease, offered the epidemiological evidence for variants with significant relationships, and evaluated the functions of significant variants using public sources.

## Materials and methods

Our research followed the guidelines of the Preferred Reporting Items for Systematic Reviews and Meta-Analyses Statement (PRISMA) and the Human Genome Epidemiology Network for systematic review of genetic association studies (16, 17).

We screened genetic association studies from Pubmed and Web of science up to 30 Apr 2022 using “{interleukin-17} OR {IL-17} OR {IL-17}” AND “{variant} OR {variation} OR {polymorphism} OR {genotype} OR {single nucleotide polymorphism} OR {SNP}”. We also collected additional articles by retrieving published reviews, meta-analyses studies, etc.

The inclusion criteria are as follows: (i) they were concentrated on the relationships between SNPs in *IL-17* family genes and susceptibility to human cancers or non-neoplastic diseases performed in case-control, cohort or cross-sectional studies (ii) they could provide the genotype data to calculate the odds ratios (ORs) and corresponding 95% confidence intervals (95% CIs) under additive genetic model, (iii) they were published in English by form of full-text. The exclusion criteria are presented, (i) they lacked sufficient information (especially the quantity of genotype and/or allelic distributions), (ii) the study was not focused on SNP in *IL-17* family genes, (iii) they were not published as full reports, such as conference abstracts and letters to editors, (iv) they concentrated on cancer mortality.

### Data extraction

Two authors extracted the data independently using a predesigned collection sheet and any disagreement could be solved with the corresponding author by discussion together. The extracted data were as follows: first author, publishing year, study design, country or region, ethnicity, gene name, variant, cases and controls, genotype counts, minor allelic frequency (MAF). When previous articles studied on the same or overlapping data, we only extracted data from papers with largest sample size and most detailed information.

### Statistical analysis

In our study, statistical tests of meta-analysis in the additive genetic association were two-tailed, and a *P* < 0.05 was significant level unless otherwise stated, which were conducted using Stata, version 15 (Stata, College Station, TX, USA). Meta-analyses were performed for variants with at least three datasets. We used the Cochran’s *Q* test to evaluate the heterogeneity between studies (18), while *I^2^* statistic was applied to quantify and evaluate the heterogeneity (19). Sensitive analyses were performed to evaluate whether the significant association was lost when excluding a single study (dataset), or the first published study, or studies deviated from the Hardy-Weinberg equilibrium (HWE) in the controls. We investigated the probability of an excess of significant findings for single meta-analysis (20). Begg’s test and Egger’s test were conducted to assess potential publication bias and small-study bias, respectively (21, 22). Moreover, *P*<0.1 as the significant level in the assessment of heterogeneity, an excess of significant findings, Begg’s test and Egger’s test.

### Assessment of epidemiological credibility

Our study graded the epidemiological credibility of significant associations identified by main meta analyses using the Venice guideline (23) and false positive report probability (FPRP) test (24) (see **Supplementary Method**).

### Functional annotation

Our study assessed the potential functional effect of variants on 6p12.2 using data from the Encyclopedia of DNA Elements (ENCODE) tool HaploReg (v4.1) (25) and the UCSC Genome browser (http://genome.ucsc.edu/). We analyzed the regions of promoter or enhancer activity, local histone modification, DNase I hypersensitivity, transcription factor binding motifs and proteins bound to these regulatory sites. In addition, we examined genome-wide cis-eQTL data in multiple tissues from two major eQTL databases: the Genotype-Tissue Expression Project (26) and the Multiple Tissue Human Expression Resource Project (27) to determine whether these genes might explain the observed associations in these loci. We used the data from the Phase 3 of the 1000 Genomes Project to perform linkage disequilibrium (LD) analysis for variants positively associated with susceptibility to cancer and noncancerous diseases in current study (28).

## Result

### Characteristics of the included studies

**Figure 1:**
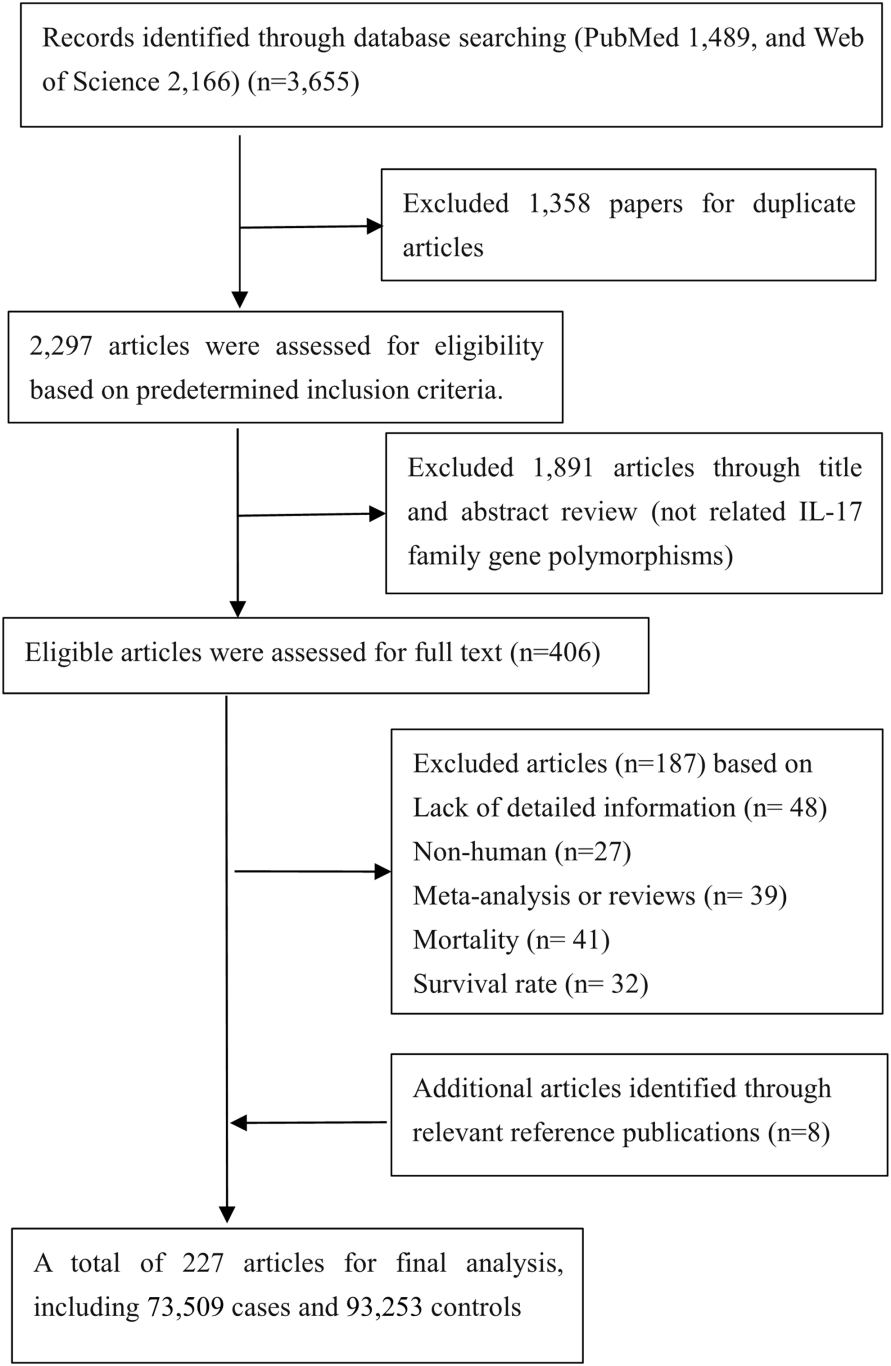
Flow diagram of search strategy and study selection.

As presented in **Figure 1**, a total of 227 eligible papers including 73,509 cases and 93,253 controls were enrolled (**Supplementary Table 1**); 135 papers focused on relationships between 10 variants in *IL-17* family genes and 25 diseases (5 cancers as well as 20 noncancerous diseases). The distributions of SNPs (*n*) with human diseases were presented: asthma (n=5), autoimmune thyroid diseases (AITD) (*n*=1), cervical cancer (*n=*3), COPD (*n=*2), colorectal cancer (*n=*2), coronary artery disease (CAD) (*n*=2), functional dyspepsia (FD) (*n=*3), gastric cancer (*n=*4), hepatitis B Virus (HBV) infection (*n=*4), hepatocellular carcinoma (*n=*1), immune thrombocytopenia (ITP) (*n*=3), inflammatory bowel disease (IBD) (*n=*3), leprosy (*n*=2), lung cancer (*n=*4), multiple sclerosis (MS) (*n=*1), osteoarthritis (OA) (*n=*2), periodontitis (*n=*2), pre-eclampsia (PE) (*n=*2), psoriasis (*n=*1), recurrent miscarriage (RM) (*n=*1), rheumatoid arthritis (RA) (*n=*5), spondyloarthritis (SpA) (*n=*2), systemic lupus erythematosus (SLE) (*n=*1), tuberculosis (TB) (*n=*3), Type 1 diabetes mellitus (T1DM) (n=1).

### Associations between *IL-17* variants and risk of human diseases

we carried out meta-analyses to investigate correlations of 10 SNPs in *IL-17* family genes with 5 cancers as well as 20 non-cancer diseases based on at least 3 datasets, under an additive genetic model. As presented in **Table 1** and **Supplementary Table 2**, 7 polymorphisms (rs1889570, rs2275913, rs2397084, rs3748067, rs763780, rs8193036, rs8193037) were associated with susceptibility to 4 types of carcinoma (colorectal, cervical, lung and gastric cancer) and 14 non-cancer diseases (AITP, asthma, CAD, HBV infection, ITP, IBD, leprosy, MS, OA, psoriasis, RA, SpA, SLE, TB) (28 associations, *P*<0.05). The cervical cancer susceptibility had positive association with minor allele of rs2275913 in Asians (OR=1.391), rs3748067 (OR=1.493) and rs763780 (OR=1.184). Apart from that, rs2275913 had an elevated susceptibility to colorectal cancer (OR=1.452); the positive association could be found in Asians and mixed populations (OR=1.524, OR=2.038, respectively), rather than in Caucasians. Apart from colorectal cancer, rs2275913 had an increased predisposition to gastric cancer (OR=1.281); the positive association could be found both in Asians and Caucasians (OR=1.264, OR=1.723, respectively); this SNP could increase risk of lung cancer (OR=1.213). Moreover, rs8193037 could elevate lung carcinoma susceptibility (OR=2.129).

**Table 1:** Associations between variants in the IL-17 family genes associated with risk of human disease in meta-analysis under additive model (at least 3 datasets).

Gene	Variant	Alleles^a^	Ethnicity	MAF^b^	Number Evaluation			Disease Risk		Heterogeneity		Venice Criteria^c^	FPRP values^d^	Credibility of Evidence^e^
					Studies	Cases	Controls	OR (95%CI)	*P* value	*I ^2^* (%)	*P _Q_*		
**Asthma**														
IL17F	rs1889570	C/T	Overall	0.3826	3	1275	1376	1.109 (0.888-1.384)	0.361	73.5	0.023			
IL17F	rs1889570	C/T	Asian	0.3235	2	856	983	1.230 (1.075-1.401)	0.003	32.9	0.222	ABA	0.032	Strong
IL17F	rs1889570	C/T	Other	0.5305	1	419	393	0.915 (0.753-1.112)	0.371	NA	NA			
IL17A	rs2275913	G/A	Overall	0.3527	6	1051	1741	0.946 (0.690-1.296)	0.728	78.8	< 0.001			
IL17A	rs2275913	G/A	Asian	0.4277	3	774	883	0.976 (0.681-1.400)	0.896	78.7	0.009			
IL17A	rs2275913	G/A	Caucasian	0.2331	3	277	534	0.891 (0.419-1.896)	0.765	85.9	0.001			
IL17F	rs2397084	T/C	Overall	0.0230	3	415	565	3.220 (1.895-5.473)	< 0.001	0.0	0.965	CAA	0.110	Weak
IL17F	rs2397084	T/C	Asian	0.0045	1	221	223	3.572 (0.738-17.292)	0.114	NA	NA			
IL17F	rs2397084	T/C	Caucasian	0.0351	2	194	342	3.167 (1.805-5.556)	< 0.001	0.0	0.826			
IL17F	rs763780	T/C	Overall	0.1060	7	1714	2032	0.925 (0.670-1.277)	0.635	71.6	0.002			
IL17F	rs763780	T/C	Asian	0.1050	5	1520	1690	1.039 (0.743-1.452)	0.824	74.5	0.003			
IL17F	rs763780	T/C	Caucasian	0.0848	2	194	342	0.536 (0.294-0.979)	0.042	0.0	0.363	CAA	0.771	Weak
IL17A	rs8193036	C/T	Asian	0.2856	3	1087	1224	0.833 (0.721-0.962)	0.013	83.1	0.003	ACC	0.197	Weak
**Autoimmune thyroid diseases**														
IL17F	rs763780	T/C	Asian	0.1161	3	1265	1189	1.623 (1.101-2.393)	0.014	73.8	0.022	BCC	0.444	Weak
**Cervical cancer**														
IL17A	rs2275913	G/A	Asian	0.3086	6	1634	2235	1.391 (1.263-1.532)	< 0.001	0.0	0.617	AAA	<0.001	Strong
IL17A	rs3748067	C/T	Asian	0.1721	4	1017	1418	1.493 (1.112-2.015)	0.008	69.0	0.022	BCC	0.264	Weak
IL17F	rs763780	T/C	Asian	0.2329	5	1449	1865	1.184 (1.045-1.342)	0.008	7.7	0.363	AAA	0.135	Strong
**Chronic obstructive pulmonary disease**														
IL17A	rs2275913	G/A	Overall	0.2026	5	1404	1729	1.023 (0.778-1.344)	0.871	76.0	0.002			
IL17A	rs2275913	G/A	Asian	0.4527	1	152	201	0.624 (0.455-0.875)	0.004	NA	NA			
IL17A	rs2275913	G/A	Mixed	0.1695	4	1252	1528	1.163 (0.976-1.385)	0.091	30.1	0.231			
IL17A	rs8193036	C/T	Overall	0.7273	5	1404	1729	1.070 (0.868-1.320)	0.525	65.1	0.022			
IL17A	rs8193036	C/T	Asian	0.2985	1	152	201	1.448 (1.049-2.000)	0.025	NA	NA			
IL17A	rs8193036	C/T	Mixed	0.7833	4	1252	1528	0.995 (0.820-1.208)	0.960	52.5	0.097			
**Colorectal cancer**														
IL17A	rs2275913	G/A	Overall	0.2714	7	1345	1535	1.452 (1.178-1.790)	< 0.001	65.1	0.009	ACC	0.014	Moderate
IL17A	rs2275913	G/A	Asian	0.3250	3	630	825	1.524 (1.155-2.011)	0.003	63.0	0.067			
IL17A	rs2275913	G/A	Caucasian	0.2120	3	598	610	1.254 (0.871-1.805)	0.223	69.8	0.036			
IL17A	rs2275913	G/A	Mixed	0.1850	1	117	100	2.038 (1.298-3.198)	0.002	NA	NA			
IL17F	rs763780	T/C	Overall	0.1880	6	1841	1307	1.267 (0.933-1.720)	0.130	58.9	0.033			
IL17F	rs763780	T/C	Asian	0.1417	3	1422	867	1.270 (0.825-1.956)	0.278	77.1	0.011			
IL17F	rs763780	T/C	Caucasian	0.0754	2	302	340	1.403 (0.655-3.004)	0.384	65.8	0.087			
IL17F	rs763780	T/C	Mixed	0.9700	1	117	100	1.003 (0.331-3.035)	0.996	NA	NA			
**Coronary artery disease**														
IL17A	rs2275913	G/A	Overall	0.3293	8	3654	3298	1.179 (1.026-1.354)	0.020	69.4	0.002	ACA	0.272	Weak
IL17A	rs2275913	G/A	Asian	0.3178	6	2534	2411	1.197 (1.053-1.360)	0.006	52.1	0.064			
IL17A	rs2275913	G/A	Caucasian	0.2841	1	220	220	1.527 (1.150-2.026)	0.003	NA	NA			
IL17A	rs2275913	G/A	Mixed	0.1904	1	900	667	0.870 (0.724-1.045)	0.137	NA	NA			
IL17A	rs3748067	C/T	Asian	0.6603	4	1197	1170	0.723 (0.572-0.913)	0.006	55.3	0.082	ACA	0.140	Weak
**Functional dyspepsia**														
IL17A	rs2275913	G/A	Asian	0.3936	3	175	564	0.815 (0.632-1.052)	0.116	0.0	0.943			
IL17F	rs2397084	T/C	Other	0.0860	3	237	695	0.885 (0.290-2.702)	0.831	79.1	0.008			
IL17F	rs763780	T/C	Overall	0.1178	6	412	1314	0.891 (0.688-1.154)	0.383	0.0	0.667			
IL17F	rs763780	T/C	Asian	0.1257	3	175	564	0.676 (0.447-1.022)	0.063	0.0	0.998			
IL17F	rs763780	T/C	Other	0.1120	3	237	750	1.089 (0.779-1.522)	0.617	0.0	0.952			
**Gastric cancer**														
IL17A	rs2275913	G/A	Overall	0.3845	18	6207	8902	1.281 (1.179-1.392)	< 0.001	60.5	< 0.001	ACA	<0.001	Moderate
IL17A	rs2275913	G/A	Asian	0.3855	17	6046	8731	1.264 (1.165-1.372)	< 0.001	58.4	0.001			
IL17A	rs2275913	G/A	Caucasian	0.3333	1	161	171	1.723 (1.258-2.358)	0.001	NA	NA			
IL17A	rs3748067	C/T	Asian	0.3385	11	3022	5284	0.981 (0.750-1.283)	0.889	83.6	< 0.001			
IL17A	rs4711998	A/G	Asian	0.3316	3	1750	2066	1.001 (0.906-1.105)	0.989	41.7	0.180			
IL17F	rs763780	T/C	Asian	0.6781	10	3944	4566	1.042 (0.805-1.348)	0.756	87.4	< 0.001			
**Hepatitis B Virus infection**														
IL17A	rs2275913	G/A	Overall	0.4986	13	2633	2479	0.968 (0.834-1.124)	0.671	68.4	< 0.001			
IL17A	rs2275913	G/A	Asian	0.5085	12	2434	2307	0.964 (0.821-1.133)	0.660	70.7	< 0.001			
IL17A	rs2275913	G/A	Caucasian	0.3663	1	199	172	1.024 (0.760-1.381)	0.875	NA	NA			
IL17A	rs4711998	A/G	Asian	0.2324	4	533	617	1.068 (0.678-1.683)	0.776	81.9	0.001			
IL17F	rs763780	T/C	Overall	0.1727	7	1445	1546	0.852 (0.739-0.982)	0.027	82.0	< 0.001	BBC	0.082	Weak
IL17F	rs763780	T/C	Asian	0.1727	6	1246	1374	0.826 (0.713-0.957)	0.011	83.8	< 0.001			
IL17F	rs763780	T/C	Caucasian	0.0610	1	199	172	1.345 (0.760-2.379)	0.309	NA	NA			
IL17A	rs8193036	C/T	Asian	0.2656	6	1260	945	0.947 (0.707-1.269)	0.717	74.7	0.001			
**Hepatocellular carcinoma**														
IL17A	rs2275913	G/A	Asian	0.5100	4	462	450	1.108 (0.755-1.626)	0.600	72.9	0.011			
**Immune thrombocytopenia**														
IL17F	rs763780	T/C	Overall	0.2721	6	568	748	0.683 (0.290-1.608)	0.382	89.5	< 0.001			
IL17F	rs763780	T/C	Asian	0.3186	3	413	473	0.486 (0.362-0.653)	< 0.001	0.0	0.966	CAC	0.002	Moderate
IL17F	rs763780	T/C	African	0.1916	3	155	274	0.975 (0.110-8.619)	0.382	94.0	< 0.001			
**Inflammatory Bowel Disease**														
IL17A	rs2275913	G/A	Asian	0.4202	3	580	764	1.051 (0.783-1.412)	0.740	71.1	0.031			
IL17F	rs763780	T/C	Overall	0.0840	5	1108	2928	0.877 (0.728-1.055)	0.164	51.9	0.081			
IL17F	rs763780	T/C	Asian	0.1531	3	393	994	0.750 (0.585-0.962)	0.024	51.9	0,125	CCC	0.352	Weak
IL17F	rs763780	T/C	Caucasian	0.0486	2	715	1934	1.081 (0.817-1.429)	0.587	0.0	0.417			
IL17A	rs8193036	C/T	Asian	0.2854	3	597	1016	1.012 (0.764-1.340)	0.936	65.1	0.057			
**Leprosy**														
IL17A	rs2275913	G/A	Mixed	0.2304	3	132	369	0.971 (0.726-1.299)	0.841	6.9	0.342			
IL17F	rs763780	T/C	Overall	0.1791	5	444	537	0.364 (0.268-0.496)	< 0.001	80.8	< 0.001	CCC	<0.001	Moderate
IL17F	rs763780	T/C	Mixed	0.0707	3	304	369	0.870 (0.537-1.409)	0.571	0.0	0.884			
IL17F	rs763780	T/C	Other	0.4167	2	140	168	0.200 (0.131-0.303)	< 0.001	0.0	0.936			
**Lung cancer**														
IL17F	rs12203582	G/A	Asian	0.5968	4	320	1432	1.103 (0.918-1.325)	0.293	0.0	0.790			
IL17A	rs2275913	G/A	Overall	0.3778	5	559	1690	1.213 (1.039-1.375)	0.014	0.0	0.918	AAA	0.046	Strong
IL17A	rs2275913	G/A	Asian	0.4092	4	320	1432	1.260 (1.053-1.509)	0.012	0.0	0.963			
IL17A	rs2275913	G/A	Caucasian	0.2035	1	239	258	1.088 (0.802-1.477)	0.586	NA	NA			
IL17A	rs3819024	A/G	Asian	0.4426	3	322	1098	0.940 (0.784-1.127)	0.503	0.0	0.832			
IL17A	rs8193037	G/A	Asian	0.1120	3	322	1098	2.129 (1.677-2.702)	< 0.001	49.5	0.138	BBA	<0.001	Strong
**Multiple sclerosis**														
IL17F	rs763780	T/C	Overall	0.1414	4	774	1064	1.154 (0.608-2.193)	0.661	85.1	< 0.001			
IL17F	rs763780	T/C	Asian	0.1386	2	691	874	0.687 (0.548-0.862)	0.001	0.0	0.884	BAA	0.036	Strong
IL17F	rs763780	T/C	African	0.1737	2	83	190	2.064 (1.348-3.161)	0.001	0.0	0.682			
**Osteoarthritis**														
IL17A	rs2275913	G/A	Overall	0.3448	6	1791	2324	1.232 (1.024-1.483)	0.027	71.7	0.003	ACA	0.347	Weak
IL17A	rs2275913	G/A	Asian	0.3665	3	1306	1383	1.411 (1.105-1.804)	0.006	77.8	0.011			
IL17A	rs2275913	G/A	Caucasian	0.3130	3	485	941	1.048 (0.839-1.310)	0.680	38.8	0.195			
IL17F	rs763780	T/C	Overall	0.1088	7	2671	4420	1.344 (1.111-1.626)	0.002	57.1	0.030	ACA	0.049	Moderate
IL17F	rs763780	T/C	Asian	0.1348	4	2102	3237	1.239 (1.026-1.497)	0.026	57.3	0.071			
IL17F	rs763780	T/C	Caucasian	0.0376	3	569	1138	1.754 (1.157-2.661)	0.008	39.3	0.193			
**Periodontitis**														
IL17A	rs2275913	G/A	Overall	0.3253	11	935	971	1.304 (0.892-1.908)	0.171	83.0	< 0.001			
IL17A	rs2275913	G/A	Caucasian	0.3547	3	535	524	0.942 (0.782-1.134)	0.526	0.0	0.744			
IL17A	rs2275913	G/A	Mixed	0.2920	4	300	347	1.040 (0.514-2.103)	0.914	84.0	< 0.001			
IL17A	rs2275913	G/A	Other	0.2800	4	100	100	2.498 (0.993-6.235)	0.052	74.3	0.009			
IL17F	rs763780	T/C	Overall	0.0563	5	500	507	1.251 (0.840-1.860)	0.270	0.0	0.520			
IL17F	rs763780	T/C	Mixed	0.0656	4	300	347	1.506 (0.955-2.373)	0.078	0.0	0.935			
IL17F	rs763780	T/C	Caucasian	0.0375	1	200	160	0.658 (0.281-1.543)	0.336	NA	NA			
**Pre-eclampsia**														
IL17A	rs2275913	G/A	Overall	0.4129	3	1923	2296	1.009 (0.919-1.108)	0.848	0.0	0.860			
IL17A	rs2275913	G/A	Asian	0.4111	2	1662	2018	1.020 (0.922-1.129)	0.702	0.0	0.896			
IL17A	rs2275913	G/A	Caucasian	0.4245	1	261	278	0.950 (0.745-1.210)	0.676	NA	NA			
IL17F	rs763780	T/C	Overall	0.0979	3	1923	2296	0.995 (0.852-1.162)	0.947	54.4	0.111			
IL17F	rs763780	T/C	Asian	0.1017	2	1662	2018	0.938 (0.793-1.109)	0.451	0.0	0.342			
IL17F	rs763780	T/C	Caucasian	0.0737	1	261	278	1.449 (0.948-2.217)	0.087	NA	NA			
**Psoriasis**														
IL17F	rs763780	T/C	Overall	0.1219	6	1151	975	1.499 (0.899-2.499)	0.121	80.2	< 0.001			
IL17F	rs763780	T/C	Asian	0.0955	2	324	363	1.571 (1.118-2.207)	0.009	0.0	0.371	BAA	0.307	Weak
IL17F	rs763780	T/C	Caucasian	0.0274	2	601	462	1.378 (0.811-2.343)	0.236	0.0	0.371			
IL17F	rs763780	T/C	African	0.0750	1	60	60	4.111 (1.856-9.105)	< 0.001	NA	NA			
IL17F	rs763780	T/C	Other	0.4567	1	166	150	0.676 (0.492-0.929)	0.016	NA	NA			
**Recurrent Miscarriage**														
IL17A	rs2275913	G/A	Overall	0.4725	3	290	309	1.148 (0.753-1.752)	0.520	65.6	0.055			
IL17A	rs2275913	G/A	Caucasian	0.5265	2	170	189	0.924 (0.666-1.282)	0.634	0.0	0.451			
IL17A	rs2275913	G/A	African	0.3875	1	120	120	1.634 (1.137-2.348)	0.008	NA	NA			
**Rheumatoid arthritis**														
IL17A	rs2275913	G/A	Overall	0.3661	13	3826	4011	0.862 (0.833-0.955)	0.001	6.8	0.378	AAA	0.001	Strong
IL17A	rs2275913	G/A	Asian	0.4633	1	615	839	0.875 (0.754-1.016)	0.080	NA	NA			
IL17A	rs2275913	G/A	Caucasian	0.3908	8	2452	2389	0.896 (0.824-0.974)	0.010	27.4	0.209			
IL17A	rs2275913	G/A	Mixed	0.2687	4	759	783	0.897 (0.744-1.082)	0.255	5.0	0.368			
IL17F	rs2397084	T/C	Caucasian	0.1056	4	800	695	1.575 (0.499-4.968)	0.439	94.9	< 0.001			
IL17A	rs3819024	A/G	Overall	0.3712	3	2053	2266	0.914 (0.834-1.002)	0.056	0.0	0.603			
IL17A	rs3819024	A/G	Asian	0.4816	1	615	839	0.880 (0.758-1.021)	0.092	NA	NA			
IL17A	rs3819024	A/G	Caucasian	0.3842	1	937	928	0.913 (0.800-1.043)	0.179	NA	NA			
IL17A	rs3819024	A/G	Mixed	0.1513	1	501	499	1.019 (0.798-1.300)	0.882	NA	NA			
IL17F	rs763780	T/C	Overall	0.0713	9	1297	1264	1.650 (0.792-3.437)	0.181	89.9	< 0.001			
IL17F	rs763780	T/C	Caucasian	0.0751	6	1039	980	2.070 (0.794-5.395)	0.137	92.9	< 0.001			
IL17F	rs763780	T/C	Mixed	0.0581	3	258	284	0.977 (0.527-1.812)	0.941	23.4	0.271			
IL17A	rs8193036	C/T	Overall	0.3941	3	2052	2261	1.074 (0.905-1.276)	0.414	65.8	0.054			
IL17A	rs8193036	C/T	Asian	0.2555	1	615	839	1.250 (1.056-1.479)	0.010	NA	NA			
IL17A	rs8193036	C/T	Caucasian	0.2974	1	936	923	0.953 (0.827-1.097)	0.502	NA	NA			
IL17A	rs8193036	C/T	Mixed	0.7986	1	501	499	1.050 (0.843-1.309)	0.662	NA	NA			
**Spondyloarthritis**														
IL17A	rs2275913	G/A	Mixed	0.2500	4	439	784	1.401 (1.156-1.698)	0.001	25.2	0.260	BBA	0.015	Strong
IL17F	rs763780	T/C	Mixed	0.0522	4	439	788	3.684 (2.737-4.960)	< 0.001	0.0	0.431	BAA	<0.001	Strong
**Systemic lupus erythematosus**														
IL17A	rs2275913	G/A	Overall	0.2338	5	941	1289	1.160 (1.007-1.336)	0.040	53.5	0.072	ACA	0.429	Weak
IL17A	rs2275913	G/A	African	0.2807	3	515	695	1.281 (1.071-1.533)	0.007	63.2	0.066			
IL17A	rs2275913	G/A	Caucasian	0.3105	1	59	95	0.975 (0.592-1.604)	0.920	NA	NA			
IL17A	rs2275913	G/A	Mixed	0.1573	1	367	499	0.985 (0.758-1.281)	0.910	NA	NA			
**Tuberculosis**														
IL17A	rs2275913	G/A	Overall	0.4066	12	4240	4983	0.998 (0.854-1.165)	0.975	79.5	< 0.001			
IL17A	rs2275913	G/A	Asian	0.4283	5	3137	3549	1.066 (0.894-1.270)	0.478	80.4	< 0.001			
IL17A	rs2275913	G/A	Caucasian	0.4039	5	727	1052	1.120 (0.799-1.572)	0.511	79.4	0.001			
IL17A	rs2275913	G/A	Mixed	0.2287	2	376	382	0.594 (0.399-0.884)	0.010	48.6	0.163	BBA	0.406	Weak
IL17A	rs3748067	C/T	Asian	0.1429	3	2365	2305	1.244 (0.939-1.649)	0.129	71.4	0.030			
IL17F	rs763780	T/C	Overall	0.0990	8	3574	4238	1.380 (1.084-1.758)	0.009	72.0	0.001	ACA	0.188	Weak
IL17F	rs763780	T/C	Asian	0.1139	5	3137	3549	1.426 (1.082-1.879)	0.012	0.0	0.665			
IL17F	rs763780	T/C	Caucasian	0.0225	3	437	689	1.088 (0.607-1.951)	0.776	83.3	< 0.001			
**Type 1 diabetes mellitus**														
IL17A	rs2275913	G/A	Overall	0.3288	3	155	184	1.223 (0.887-1.685)	0.219	31.4	0.233			
IL17A	rs2275913	G/A	Other	0.2333	2	30	30	0.580 (0.229-1.466)	0.249	0.0	0.782			
IL17A	rs2275913	G/A	Caucasian	0.3474	1	125	154	1.360 (0.965-1.918)	0.079	NA	NA			

OR, odds ratio; A, adenine; T, thymine; G, guanine; C, cytosine; NA, not applicable.

**^a^** Major alleles (reference)/minor alleles.

**^b^** Frequency of minor allele in controls.

**^c^** Strength of epidemiological evidence based on the Venice criteria.

**^d^** FPRP values at prior probability of 0.05 at power OR of 1.5, and the FPRP level of noteworthiness is 0.20.

**^e^** Degree of epidemiological credibility based on the combination of results from Venice guidelines and FPRP tests.

For non-cancer disease, current results showed that significant relationships with asthma risk were found for rs1889570 in Asians (OR=1.230), rs2397084 (OR=3.220), rs763780 (OR=0.536) and rs8193036 (OR=0.833). In addition, rs763780 had an elevated susceptibility to AITD (OR=1.623) in Asians. For CAD, significant relationships were found for rs2275913 (OR=1.179). Additionally, rs3748067 could reduce risk of CAD in Asians (OR=0.723). For HBV infection, rs763780 had a decreased susceptibility to HBV infection (OR=0.852), especially among Asians (OR=0.826). Apart from that, rs763780 could decrease susceptibility to ITP (OR=0.486) and risk of IBD (OR=0.750) in Asians. Apart from that, rs763780 had a reduced susceptibility to leprosy (OR=0.364) and risk of MS (OR=0.687), respectively. Interestingly, current results showed that rs2275913 could increase risk of OA (OR=1.232), SpA (OR=1.401) and SLE (OR=1.160), whereas decrease susceptibility to RA (OR=0.862) and TB (OR=0.594); rs763780 could increase risk of OA (OR=1.344), psoriasis (OR=1.571), SpA (OR=3.684) and TB (OR=1.380), respectively.

Additionally, 8 SNPs (rs12203582, rs2275913, rs2397084, rs3748067, rs3819024, rs8193036, rs763780 and rs4711998) had no association with susceptibility to 14 human diseases (asthma, COPD, colorectal cancer, FD, gastric cancer, HBV infection, hepatocellular carcinoma, IBD, lung cancer, PE, RM, RA, TB and T1DM) in additive model. Of these, 3 SNPs had no association with 3 diseases (rs3748067 and rs763780 for gastric cancer; rs2275913 for HBV infection; rs3748067 for TB) with at least 2,300 case and 2,300 controls. Also, we calculated the statistical power to confirm whether the bigger sample size confirming these relationships is required in next study (**Table 2** and **Supplementary Table 3**).

**Table 2:** Variants in IL-17 family genes showing no relation to risk of disease in meta-analyses in additive model.

Gene	Variant	Alleles^a^	Disease	Ethnicity	MAF^b^	Number Evaluation		Meta-analysis risk		Heterogeneity	
						Studies	Sample size (case/control)	OR (95%CI)	*P*_value_	I^2^(%)	P _(Q)_
IL17A	rs2275913	G/A	Asthma	Overall	0.3527	6	2792 (1051/1741)	1.108 (0.755-1.626)	0.600	72.9	0.011
IL17A	rs2275913	G/A	COPD	Overall	0.2026	5	3133 (1404/1729)	1.051 (0.783-1.412)	0.740	71.1	0.031
IL17A	rs8193036	C/T	COPD	Overall	0.7273	5	3133 (1404/1729)	1.012 (0.764-1.340)	0.936	65.1	0.057
IL17F	rs763780	T/C	Colorectal cancer	Overall	0.1880	6	3148 (1841/1307)	1.103 (0.918-1.325)	0.293	0.0	0.790
IL17A	rs2275913	G/A	Functional dyspepsia	Asian	0.3936	3	739 (175/564)	0.940 (0.784-1.127)	0.503	0.0	0.832
IL17F	rs763780	T/C	Functional dyspepsia	Overall	0.1178	6	1726 (412/1314)	1.304 (0.892-1.908)	0.171	83.0	< 0.001
IL17A	rs3748067	C/T	Gastric Cancer	Asian	0.3385	11	8306 (3022/5284)	1.251 (0.840-1.860)	0.270	0.0	0.520
IL17A	rs4711998	A/G	Gastric Cancer	Asian	0.3316	3	3816 (1750/2066)	1.009 (0.919-1.108)	0.848	0.0	0.860
IL17F	rs763780	T/C	Gastric Cancer	Asian	0.6781	10	8510 (3944/4566)	0.995 (0.852-1.162)	0.947	54.4	0.111
IL17A	rs2275913	G/A	Hepatitis B Virus infection	Overall	0.4986	13	5112 (2633/2479)	1.148 (0.753-1.752)	0.520	65.6	0.055
IL17A	rs4711998	A/G	Hepatitis B Virus infection	Asian	0.2324	4	1150 (533/617)	1.575 (0.499-4.968)	0.439	94.9	< 0.001
IL17A	rs8193036	C/T	Hepatitis B Virus infection	Asian	0.2656	6	2205 (1260/945)	1.650 (0.792-3.437)	0.181	89.9	< 0.001
IL17A	rs2275913	G/A	Hepatocellular carcinoma	Asian	0.5100	4	912 (462/450)	1.074 (0.905-1.276)	0.414	65.8	0.054
IL17A	rs2275913	G/A	Inflammatory Bowel Disease	Asian	0.4202	3	1344 (580/764)	1.244 (0.939-1.649)	0.129	71.4	0.030
IL17A	rs8193036	C/T	Inflammatory Bowel Disease	Asian	0.2854	3	1613 (597/1016)	1.223 (0.887-1.685)	0.219	31.4	0.233
IL17F	rs12203582	G/A	Lung cancer	Asian	0.5968	4	1752 (320/1432)	1.108 (0.755-1.626)	0.600	72.9	0.011
IL17A	rs3819024	A/G	Lung cancer	Asian	0.4426	3	1420 (322/1098)	1.051 (0.783-1.412)	0.740	71.1	0.031
IL17A	rs2275913	G/A	Periodontitis	Overall	0.3253	11	1906 (935/971)	1.012 (0.764-1.340)	0.936	65.1	0.057
IL17F	rs763780	T/C	Periodontitis	Overall	0.0563	5	1007 (500/507)	1.103 (0.918-1.325)	0.293	0.0	0.790
IL17A	rs2275913	G/A	Periodontitis	Overall	0.4129	3	4219 (1923/2296)	0.940 (0.784-1.127)	0.503	0.0	0.832
IL17F	rs763780	T/C	Periodontitis	Overall	0.0979	3	4219 (1923/2296)	1.304 (0.892-1.908)	0.171	83.0	< 0.001
IL17A	rs2275913	G/A	Recurrent Miscarriage	Overall	0.4725	3	599 (290/309)	1.251 (0.840-1.860)	0.270	0.0	0.520
IL17F	rs2397084	T/C	Rheumatoid arthritis	Caucasian	0.1056	4	1495 (800/695)	1.009 (0.919-1.108)	0.848	0.0	0.860
IL17F	rs763780	T/C	Rheumatoid arthritis)	Overall	0.0713	9	2561 (1297/1264)	0.995 (0.852-1.162)	0.947	54.4	0.111
IL17A	rs8193036	C/T	Rheumatoid arthritis	Overall	0.3941	3	4313 (2052/4313)	1.148 (0.753-1.752)	0.520	65.6	0.055
IL17A	rs3748067	C/T	Tuberculosis	Asian	0.1429	3	4670 (2365/2305)	1.575 (0.499-4.968)	0.439	94.9	< 0.001
IL17A	rs2275913	G/A	Type 1 diabetes mellitus	Overall	0.3288	3	339 (155/184)	1.650 (0.792-3.437)	0.181	89.9	< 0.001

OR, odds ratio; A, adenine; C, cytosine; G, guanine; T, thymine;

a Major alleles (reference)/Minor alleles.

b Frequency of minor allele in controls.

### Heterogeneity, bias and sensitivity analysis

As shown in **Table 1**, heterogeneity was investigated for 28 significant associations (7 SNPs for 18 human diseases). Mild heterogeneity (*I^2^* < 25%) was assigned to 3 variants with risk of 2 cancers and 6 noncancerous diseases (10 associations); moderate heterogeneity (25% ≤ *I^2^* ≤ 50%) was assigned to 3 variants with risk of 1 cancer and 3 noncancerous diseases (4 associations); high heterogeneity (*I^2^* > 50%) was assigned to 4 variants with risk of 3 cancers and 9 noncancerous diseases (14 associations). Moreover, the results indicated that publication bias existed (*p* < 0.10) in associations for rs3748067 and rs2275913 with colorectal cancer risk. Apart from that, sensitivity analyses indicated that some significant summary ORs were lost, including rs8193036 in asthma and AITD, rs763780 in HBV infection (excess of significant findings); rs763780 in AITD and ITP (small study), and in IBD (HWE), and in leprosy (small study).

### Cumulative evidence of association

As shown in **Table 1**, our study firstly used the Venice guideline and FPRP tests to grade epidemiological credibility of 28 significant. In terms of Venice guideline, strong, moderate and weak evidence were assigned to 4, 7 and 17 associations, respectively. Then, the probability for a true association between the 28 positive associations was investigated based on FPRP tests. The FPRP value < 0.05 was observed for 13 associations, FPRP 0.05 to 0.2 for 6 associations, and FPRP > 0.2 was found for 9 associations, respectively. At last, combing Venice guideline and FPRP tests, strong evidence was assigned to 4 variants (*IL-17F* rs1889570, *IL-17A* rs2275913, *IL-17F* rs763780, *IL-17A* rs8193037) and 2 cancer (cervical and lung cancer) as well as 4 noncancerous diseases (asthma, MS, RA, SpA), moderate to 2 SNPs (*IL-17A* rs2275913, *IL-17F* rs763780) and colorectal and gastric cancer as well as 3 noncancerous diseases (ITP, leprosy, OA), weak to 5 SNPs (*IL-17F* rs2397084, *IL-17F* rs763780, *IL-17A* rs8193036, *IL-17A* rs3748067, *IL-17A* rs2275913) and 1 cancer (cervical cancer) as well as 9 noncancerous diseases (asthma, AITD, CAD, HBV infection, IBD, OA, psoriasis, SLE, TB).

In addition, we attempted to pool the ORs and 95%CIs on the basis of two datasets and found that 9 variants (rs1889570, rs2275913, rs3819024, rs4711998, rs4819554, rs6973569, rs763780, rs8193036 and rs8193037) had significantly associated with susceptibility to 4 cancers (bladder, colorectal, papillary thyroid cancer and hepatocellular carcinoma) as well as 10 noncancerous diseases (ankylosing spondylitis, Behcet’s disease, bronchiolitis, brucellosis, chronic chagas cardiomyopathy, CAD, gastro-duodenal ulcer, recurrent miscarriage, silicosis and TB); of these, 5 SNPs (rs4819554, rs8193036, rs8193037, rs2275913, rs763780) and risk of 4 noncancerous diseases (ankylosing spondylitis, CAD, gastro-duodenal ulcer, recurrent miscarriage) were considered noteworthy (*P* < 0.2 for FPRP) (see **Supplementary Table 4**). Additionally, we calculated the ORs and 95% CI in the additive model to assess the relationships between 53 variants and susceptibility to 90 diseases (based on one dataset), yielding significant relationships between 22 variants and the risk of 47 types of carcinoma. Apart from that, *P* value of FPRP for the significant associations also be calculated. Finally, we considered the associations between 14 variants and susceptibility to 21 diseases noteworthy (see **Supplementary Table 5**).

### Functional annotation for variants with strong evidence

As shown in **Table 3**, we used the Encyclopedia of DNA Elements tool HaploReg v4.1 to assess the potential functional roles for strong evidence (4 variants with risk of 6 human diseases). For functional annotations, rs763780 was annotated as missense. The total 4 SNPs might be located in a region with strong promoter and enhancer activity, and two SNPs in alteration in regulatory motif. Subsequently, as the consequence of the function evaluation using the PolyPhen-2 web server (29), the unique non-synonymous variant rs763780 was qualitatively predicted to be “benign” with a naïve Bayes posterior probability of less than 0.15. As shown in **Supplementary Figure 1**, the linkage disequilibrium (LD) plots presented that the regions represented by significant SNPs had distinct genetic structures among in European, Asian and African ancestry. The information extracted from the Genotype-Tissue Expression Project shows that rs2275913, rs763780, rs8193037, rs1889570 are eQTLs for the *IL-17A, IL-17F, GSTA8P, MCM3*. In addition, rs2275913 had an increased expression in *GSTA8P, IL-17A* genes in testis tissues; rs763780 and rs8193037 had an increased expression in *MCM3,IL-17F* genes in muscle and esophagus tissues, respectively (**Supplementary Table 6**). In our study, rs2275913 and rs763780 had significantly associated with susceptibility to cervical cancer and SpA. The Phase 3 of the 1000 Genomes Project (30) (**Supplementary Table 7**) indicated *IL-17A* rs2275913 is uncorrelated with *IL-17F* rs763780 in Europeans, East Asians and Africans (*r* ^2^< 0.05 for all tests). Moreover, *IL-17A* rs2275913 and *IL-17A* rs8193037 had associated with predisposition to lung cancer. We also found that rs2275913 is weak LD with rs8193037 in East Asians (*r* ^2^ = 0.1) and is uncorrelated with rs8193037 in Europeans and Africans (*r* ^2^< 0.05).

**Table 3:** Summary of functional annotations for 4 SNPs in 6 human diseases (strong epidemiological credibility).

Variant	Gene	Position^a^	Annotation	Promoter histone marks^b^	Enhancer histone marks^c^	DNAse^d^	Proteins bound^e^	Motifs changed^f^
rs763780	IL17F	52101739	missense	ESDR, BLD	BLD, HRT			Lmo2-complex,Mtf1
rs1889570	IL17F	52110734		ESDR, IPSC, BRST	GI, LIV			HDAC2,NF-I,NRSF
rs2275913	IL17A	52051033		BLD	BLD			
rs8193037	IL17A	52051109		BLD, GI	BLD			

a chromosome position is based on NCBI Build 37.

b Histone modification of H3K4me1 and H3K27ac (tissue types: if >3, only the number is included).

c Histone modification of H3K4me3 (tissue types: if >3, only the number is included).

d Levels of DNase I hypersensitivity (tissue types: if >3, only the number is included).

e Alteration in transcription factor binding (disruptions: if >3, only the number is included).

f Alteration in regulatory motif (disruptions: if >3, only the number is included).

## Discussion

Our study performs a comprehensive research synopsis and meta-analysis, summarizes and updates the associations between SNPs in *IL-17* family genes and predisposition to human diseases for the first time, which offers precise results for the SNPs and provides more variants and diseases that never been investigated before. Our study included 227 papers with 73,509 cases and 93,253 controls and performed a meta-analysis using 135 papers with available information to assess relationships of 10 variants with susceptibility to 25 diseases (5 cancers as well as 20 non-cancer diseases); 7 SNPs had positively associated with 18 human disease predisposition. Our study used the Venice guidelines and FPRP tests to grade the cumulative evidence of significant relationships. At last, 4 SNPs were assigned to strong evidence with predisposition to 6 human diseases (9 associations: *IL-17F* rs1889570 in asthma; *IL-17A* rs2275913 in lung cancer, cervical cancer, RA, SpA; *IL-17F* rs763780 in cervical cancer, MS, SpA; *IL-17A* rs8193037 in lung cancer), moderate to 2 SNPs and 2 cancer as well as 3 noncancerous diseases, weak to 5 SNPs and 1 cancer as well as 9 noncancerous diseases. Moreover, we attempted to construct functional annotations for these 4 variants with strong evidence using data from the Encyclopedia of DNA Elements Project and other public databases and then uncovered that the SNPs with strong evidence might fall in several putative regulatory regions. In summary, this study provides updated evidence that SNPs in the *IL-17* family genes had significant associations with predisposition to lung, cervical cancer, asthma, RA, SpA and MS.

The *IL-17*, a kind of proinflammatory cytokine, plays a crucial role in both innate and acquired immune responses (31). Previous papers demonstrated that *IL-17* is activated by microbial products, and may accelerate carcinoma occurrence and development by angiogenic functions (32). The *IL-17A* gene (Gene ID: 3605) is located at chromosome 6p12.2, and the encoded protein is a proinflammatory cytokine produced by activated T cells, which might involve in the development of human diseases (31). In the previous paper, it was pointed out that *IL-17A* rs2275913 acted as risk factor for multiple cancers (gastric, cervical, colorectal and oral carcinoma) (15) and non-cancerous diseases (RA) (33). Consistent with our meta-analysis, strong evidence was assigned to *IL-17A* rs2275913 in lung cancer, cervical cancer, RA, SpA, and *IL-17A* rs8193037 in lung cancer. LD analysis indicates that *IL-17A* rs2275913 and *IL-17A* rs8193037 were associated with susceptibility to lung cancer. We also found that rs2275913 is weak LD with rs8193037 in East Asians and is uncorrelated with rs8193037 in Europeans and Africans, indicating that the functional mechanisms of the two variants associated with lung cancer risk may be distinct in different ethnic groups and partly explain why some variants are found to be associated with a cancer site in one ethnic group but not in others. Current evidence presents that high expression of *IL-17A* is linked with the development and progression of cancers, and *IL-17A* could be regulated at the transcriptional level (34). *IL-17A* rs2275913 could influence the expression of the *IL-17A* protein and trigger cell transformation and maintain the autonomous proliferation of the transformed cells, and thus increase the susceptibility of cervical cancer, especially in HPV infection individuals (35). *IL-17A* could influence the transcriptional activity of *NFAT* and trigger the stimulation of T lymphocytes cells, which might increase risk of lung cancer (36). Moreover, a recent study indicated that the G allele polymorphism of *IL-17A* rs2275913 (a change from glutamic acid to lysine) was protective in RA individuals (37), which is consistent with our results; *IL-17A* and *TNF-α* had been considered as a predictor of a poor outcome in RA individuals; interestingly, previous study concluded that therapies targeting *IL-17* in autoimmune diseases ameliorated the inadequate response to anti-*TNF-α* therapy (38), which indicated that SNP rs2275913 could be considered as a novel target for gene therapy of RA and promote drug developments against RA. Moreover, the SNP rs2275913 A allele is linked with high IL-17 expression, which has an elevated susceptibility to autoimmune and inflammatory diseases, including SpA (39). Moreover, previous papers found that drugs target other molecules of the immune system, such as anti-IL-17A (ixekizumab and secukinumab) and anti-IL-17A receptor (brodalumab). The efficacy of anti-IL-17R and anti-IL-17 agents has been shown in Phase II (40) and III trials (41, 42), indicating that IL-17A might have impact on the pathogenesis of psoriasis.

The *IL-17F* gene (Gene ID:112744) is located at chromosome 6p12.2, and the protein encoded by *IL-17F* gene is a cytokine activated by T cells. It could stimulate the production of other cytokines, such as *IL-6*, *IL-8*, and *CSF2/GM_CSF* (31, 43). These cytokines might have similar synergistic effects on risk of human diseases (44). In the previous paper, it was pointed out that *IL-17F* rs763780 might trigger the development of cervical and oral carcinoma (15) and non-cancerous diseases (such as asthma) (45). Consistent with our meta-analysis, strong evidence was assigned to *IL-17F* rs1889570 in asthma, and *IL-17F* rs763780 in cervical cancer, MS, SpA. In our study, rs2275913 and rs763780 had positive association with susceptibility to SpA and cervical cancer. LD analysis indicates that *IL-17A* rs2275913 is uncorrelated with *IL-17F* rs763780 in Europeans, East Asians and Africans, demonstrating that there might be different causal variants and functional mechanisms involved in relationships of variants in the *IL-17A*-*IL-17F* genes with risk of cervical cancer and SpA. Current evidence presents that *IL-17F* rs1889570 could increase risk of asthma by influencing the expression of proinflammatory cytokines, chemokines, and growth factors associated with leukocyte activation and airway remodeling (46). Additionally, a previous study found that the *IL-17F* rs763780, a missense located in the *IL-17F* exon3 region, could trigger high *IL-17* expression which influenced cervical cancer cell growth, and thus increased risk of cervical cancer (47). Moreover, the SNP rs763780 C allele is linked with high IL-17 expression, which has proved to increase risk of SpA (39).

Additionally, we calculated the ORs and 95% CI in the additive model to assess the relationships between 53 variants and susceptibility to 90 diseases (based on one dataset), yielding significant relationships between 22 variants and the risk of 47 types of carcinoma. For example, our results found that some non-cancerous diseases including autoimmune diseases (such as autoimmune thyroid diseases), alopecia areata, and some type of autoimmune blistering diseases (such as bullous pemphigoid), which have presented in our supplementary Tables (see **Supplementary Table 1**). Moreover, in our study, we performed meta-analysis based on at least three datasets. However, we found that alopecia areata and bullous pemphigoid only contained 1 dataset for each SNP, which could not be assessed by meta-analysis. Therefore, we just presented these information in our supplementary files (see **Supplementary Table 1** and **Supplementary Table 5**). Finally, we hope to attempt to collect more information in order to solve this issue in our study in the future. Apart from that, *P* value of FPRP for the significant associations also be calculated. In summary, we considered the associations between 14 variants and susceptibility to 21 diseases noteworthy (see **Supplementary Table 5**). Further, well-designed studies are recommended to clarify the association with multiple diseases for these variants.

Additionally, 8 SNPs had no association with susceptibility to 14 human diseases in additive model. Of these, 3 SNPs had no association with 3 diseases (rs3748067 and rs763780 for gastric cancer; rs2275913 for HBV infection; rs3748067 for TB) with at least 2,300 cases and 2,300 controls, which presented over 80% statistical power to detect an OR of 1.15 for a variant with MAF 0.20 (Type 1 error 0.05). Further study less than current individuals on these 3 variants for these 3 diseases will not yield fruitful results (**Supplementary Table 3**). Apart from that, our study identified that significant relationships for 5 variants with risk of 4 diseases (based on two datasets) and 14 variants with risk of 21 diseases (based on one dataset) were considered noteworthy, which might be required to confirm or refute these associations by large-scale studies in the future.

Some limitations apply to this research: (i) even though we conducted a comprehensive search to screen eligible papers, some articles may have been missed. Also, some malignancies and non-cancer diseases could not be completely assessed by meta-analysis owing to insufficient information (for example, lack of genotype amount, fewer than 3 datasets in some associations); (ii) only ethnicity was assessed by subgroup, other factors (such as pathological/clinical type, gene-gene or gene-environment associations and interactions) might be required to confirm or refute the relationships with susceptibility to disease; (iii) the unreasonable data, like errors in genotype, could not be investigated, and (iv) moderate and weak evidence should be explained with caution.

In summary, this large-scale meta-analysis identified that 4 SNPs in the *IL-17* family genes were graded as demonstrating strong association to 2 cancer and 4 non-cancer disease risk. Apart from that, these findings provide a foundation for further demonstrating the variations in the *IL-17* family genes are positively linked with susceptibility to cervical cancer, lung cancer, asthma, MS, RA, SpA, and highlight that the variants in *IL-17* family genes might become a valuable genetic tool to investigate the pharmacological targeting potential of *IL-17* family genes. We should further understand its biological pathway and apply these clues to clinical practice and public health for risk assessment and management.

## Data availability statement

The original contributions presented in the study are included in the article/**Supplementary Material**. Further inquiries can be directed to the corresponding author.

## Author contributions

TL, LY, and HC designed this work. TL and LY integrated and analyzed the data. TL, LY, and HC wrote this manuscript. TL, LY, XL, CZ, CJ, ZY, CF, and HC finished the related Tables and Figures. TL, LY, and HC edited and revised the manuscript. All authors approved this manuscript.

## Funding

This study was supported by funding from the Chongqing Natural Science Foundation (grant No. cstc2020jcyj-msxmX0257).

## Conflict of interest

The authors declare that the research was conducted in the absence of any commercial or financial relationships that could be construed as a potential conflict of interest.

## Publisher’s note

All claims expressed in this article are solely those of the authors and do not necessarily represent those of their affiliated organizations, or those of the publisher, the editors and the reviewers. Any product that may be evaluated in this article, or claim that may be made by its manufacturer, is not guaranteed or endorsed by the publisher.
