# Supplementary material for: Cumulative evidence for associations between genetic variants in interleukin 17 family gene and risk of human diseases
Source: Front Immunol. 2022 Oct 10;13:1008184. doi: 10.3389/fimmu.2022.1008184 (PMC9589504; doi:10.3389/fimmu.2022.1008184)
Supplement: Supplementary file 1 [file DataSheet_1.pdf]

Supplementary Materials to:

Cumulative Evidence for Associations Between Genetic Variants in Interleukin 17 Family Gene and Risk of Human Diseases

Tianyu Liu[1#], Lei Yang[1#], Xiaolong Lv[1#], Chunjian Zuo[2], Chenhao Jia[1], Zelin Yang[1], Chongqi Fan[1], Huanwen Chen[1*]

[1]Department of Cardiothoracic Surgery, The First Affiliated Hospital of Chongqing Medical University, Chongqing 400010, China.

[2]Department of Thoracic Surgery, Army Medical Center of PLA, No.10, Daping Changjiang Branch Road, Yuzhong District, Chongqing 400042, China.

[#] These authors have contributed equally to this work.

[*]**Correspondence:**

Huanwen Chen

Department of Cardiothoracic Surgery, The First Affiliated Hospital of Chongqing Medical University, Chongqing 400010, China.

Email address: coolstarchw9527@163.com

Contents

Supplementary Method

Method for Venice Criteria

Method for false positive report probability (FPRP) test

Supplementary Figure and Tables

Supplementary Figure 1

Supplementary Table 1. Characteristics of the included articles.

Supplementary Table 2: Associations between variants in the *IL17* famliy genes associated with risk of human disease in meta-analysis under additive model (at least 3 datasets).

Supplementary Table 3: Variants in *IL-17* family genes showing no relation to risk of disease in meta -analyses in additive model (at least 3 datasets)

Supplementary Table 4: Associations between variants in the *IL17* family genes associated with risk of human disease in meta -analysis under additive model based on two datasets.

Supplementary Table 5: Associations between variants in the *IL17* family genes associated with risk of human disease in meta-analysis under additive model based on one dataset.

Supplementary Table 6. Analyses of expression quantitative trait locus (eQTL) in significant variants associated with risk of cancer and non-neoplastic diseases.

Supplementary Table 7. Correlations (r^2) among the four variants showing strong evidence using data from the 1000 Genomes Project.

Supplementary Method:
Method for Venice Criteria

We applied the Venice Criteria to evaluate the epidemiological credibility of significant associations identified by meta-analysis [(1)]. Specifically, the level of credibility was defined as strong, moderate or weak on the basis of three criteria including amount of evidence, replication and protection from bias, with each criterion assigned with grades of A, B or C. The criterion of amount of evidence was graded by the total number of the tested alleles or genotypes in cases and controls: A for >1000, B for 100–1000 and C for <100. The criterion of replication was graded by the I^2 value: A for $I^2 < 25\%$, B for I^2 between 25% and 50% and C for $I^2 > 50\%$. It might be reasonable to assign grade A to associations with moderate or high heterogeneity on the criterion of replication if they had extensive replication record such as GWAS and GWAS meta-analysis from large collaborative efforts [(2)]. The criterion of protection from bias was graded by a series of sensitivity analyses and bias tests: (i) grade A would be assigned if there was no evident bias or the bias could not explain the existence of association; (ii) grade B would be assigned if there was moderate bias and (iii) grade C would be assigned if bias was obvious or the bias could affect the presence of association. We also took the magnitude of an association into consideration when assessing the protection from bias. If the summary OR was <1.15 (or >0.87 in a protection effect), grade C would be assigned on this criterion except that this association had been replicated extensively by large collaborative studies including GWAS or GWAS meta-analysis, showing no evidence of bias [(2)]. Consequently, the level of credibility of epidemiological evidence was considered to be strong if grades of A were assigned to all three criteria, moderate if the grades were A or B, and weak if there was grade C for any category [(3)].

The checklist of Venice Criteria was presented as follow,

Amount of evidence

A: Large-scale evidence — minor genetic group (alleles or genotypes) in cases and controls > 1,000.

B: Moderate amount of evidence — minor genetic group in cases and controls between 100 and 1,000.

C: Little evidence — minor genetic group in cases and controls < 100.

Replication of association

A: Little between-study heterogeneity — $I^2 < 25\%$.

B: Moderate between-study heterogeneity — I^2 between 25% and 50%.

C: Large between-study heterogeneity — $I^2 > 50\%$.

Qualitative epidemiologic considerations about the presence of heterogeneity and potential explanation for heterogeneity would need to be taken into account in judging replication. It may be reasonable to grade as A on this criterion for associations with moderate or high heterogeneity with an extensive replication record such as associations identified by GWAS or large GWAS meta-analysis from collaborative studies.

Protection from bias

A: No observable bias and bias was unlikely to explain the presence of the association. B: No obvious bias may affect the presence of the association, but there is considerable missing information on the identification of evidence. C: Bias is demonstrable or is likely to explain the presence of the association. The Venice criteria include an extensive checklist for sources of bias in different settings. The checklist has different considerations depending on whether the evidence comes from retrospective meta-analyses of published data or prospective GWAS and replication studies from collaborative consortia with harmonization of data collection and analysis.

General checks for bias that have been adopted for meta-analysis are: (1) Association lost with exclusion of first study; (2) Association lost with exclusion of studies deviated from HWE; (3) Small effect size of association (i.e., $0.87 < OR < 1.15$); (4) Evidence of publication bias ($p < 0.10$ in Begg's test); (5) Evidence of small-study effect ($p < 0.10$ in Egger's test); (6) Evidence is presented for an excess of individual studies with significant findings ($p < 0.10$ in significant bias test).

Method for false positive report probability (FPRP) test

A prior probability of 0.05 and a false positive report probability (FPRP) cut-off value of 0.2 in FPRP assay should be performed to detect the potential false positive results among statistical associations and assess whether these associations should be excluded, as Wacholder et al. recommended [(4)]. If the calculated FPRP value was below the prespecified noteworthiness value of 0.2, we would consider the association noteworthy, indicating the association might be true [(4)]. The true evidence was graded by FPRP value: <0.05, 0.05 - 0.2, >0.2, indicating strong, moderate, or weak, respectively. Cumulative evidence could be upgraded from moderate to strong or from weak to moderate based on a strong FPRP (<0.05). Otherwise, cumulative evidence could be downgraded from strong to moderate or from moderate to weak based on weak FPRP. The cumulative epidemiological evidence remained the same with the result of the Venice criteria for associations with moderate evidence of FPRP. We used the Excel calculator provided by Wacholder *et al*. to calculate the FPRP value and statistical power (>0.2) [(4)].

References:

1. Ioannidis-John PA, Boffetta P, Little J, O'Brien TR, Uitterlinden AG, Vineis P, Balding DJ, Chokkalingam A, Dolan SM, Flanders WD, Higgins-Julian PT, McCarthy MI, McDermott DH, Page GP, Rebbeck TR, Seminara D, Khoury MJ. Assessment of cumulative evidence on genetic associations: interim guidelines. Int J Epidemiol **2008**;37:120-32.
2. Khoury MJ, Bertram L, Boffetta P, Butterworth AS, Chanock SJ, Dolan SM, et al. Genome-wide association studies, field synopses, and the development of the knowledge base on genetic variation and human diseases. Am J Epidemiol 2009;170:269-79.
3. Cui HJ, Tang MS, Zhang M, Liu SS, Chen SY, Zeng ZQ, et al. Variants in the PSCA gene associated with risk of cancer and nonneoplastic diseases: systematic research synopsis, meta-analysis and epidemiological evidence. Carcinogenesis 2019;40:70-83.
4. Wacholder S, Chanock S, Garcia-Closas M, Ghormli LE, Rothman N. Assessing the probability that a positive report is false: an approach for molecular epidemiology studies. J Natl Cancer Inst 2004;96:434-42.

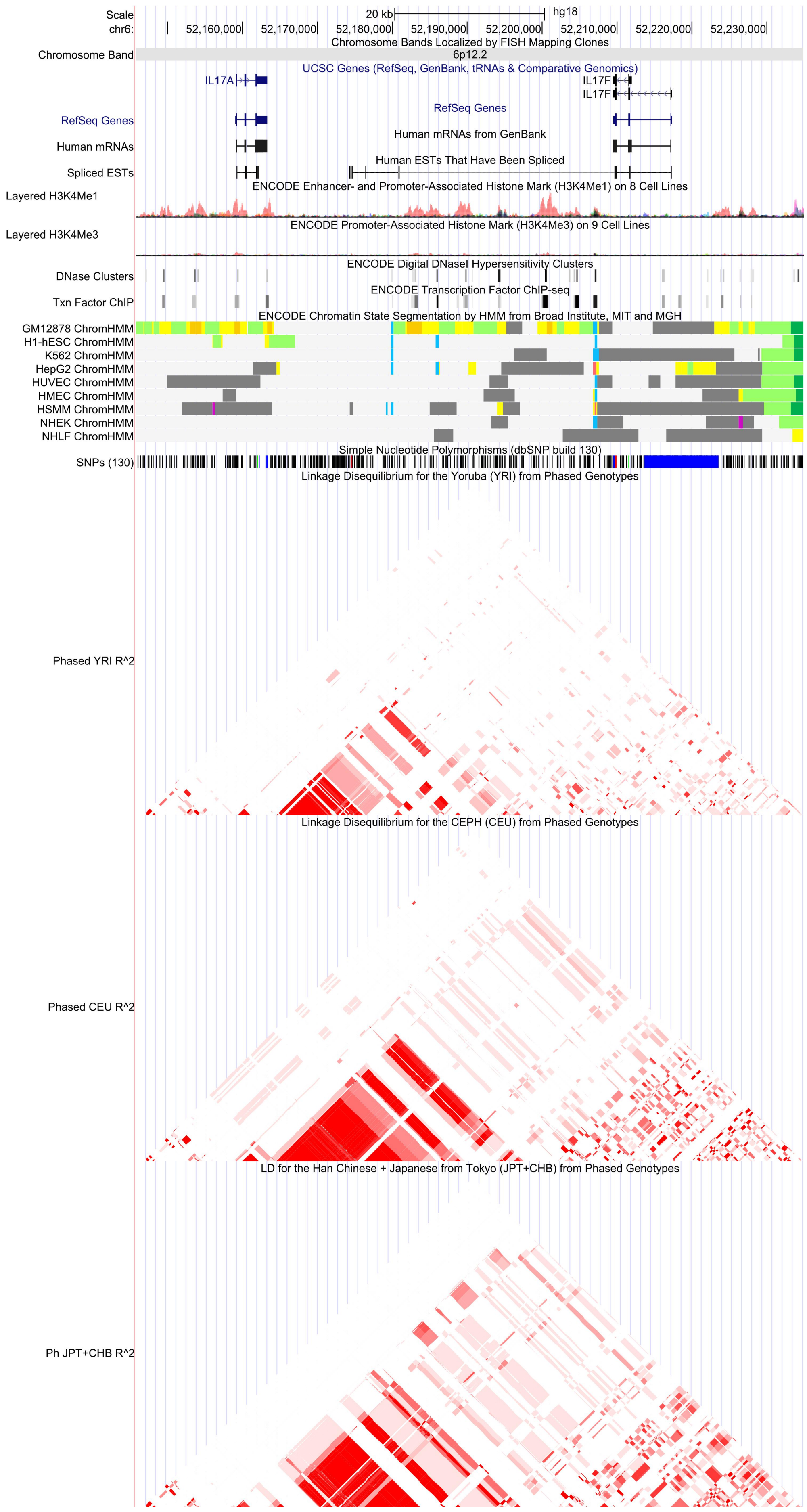
Scale
chr6:
20 kb
hg18
52,160,000
52,170,000
52,180,000
52,190,000
52,200,000
52,210,000
52,220,000
52,230,000
Chromosome Bands Localized by FISH Mapping Clones
Chromosome Band
6p12.2
UCSC Genes (RefSeq, GenBank, tRNAs & Comparative Genomics)
IL17A
IL17F
IL17F
RefSeq Genes
RefSeq Genes
Human mRNAs from GenBank
Human mRNAs
Human ESTs That Have Been Spliced
Spliced ESTs
ENCODE Enhancer- and Promoter-Associated Histone Mark (H3K4Me1) on 8 Cell Lines
Layered H3K4Me1
ENCODE Promoter-Associated Histone Mark (H3K4Me3) on 9 Cell Lines
Layered H3K4Me3
ENCODE Digital DNaseI Hypersensitivity Clusters
DNase Clusters
ENCODE Transcription Factor ChIP-seq
Txn Factor ChIP
ENCODE Chromatin State Segmentation by HMM from Broad Institute, MIT and MGH
GM12878 ChromHMM
H1-hESC ChromHMM
K562 ChromHMM
HepG2 ChromHMM
HUVEC ChromHMM
HMEC ChromHMM
HSMM ChromHMM
NHEK ChromHMM
NHLF ChromHMM
Simple Nucleotide Polymorphisms (dbSNP build 130)
SNPs (130)
Linkage Disequilibrium for the Yoruba (YRI) from Phased Genotypes
Phased YRI R^2
Linkage Disequilibrium for the CEPH (CEU) from Phased Genotypes
Phased CEU R^2
LD for the Han Chinese + Japanese from Tokyo (JPT+CHB) from Phased Genotypes
Ph JPT+CHB R^2

Figure S1: Evidence from the Encyclopedia of DNA Elements (ENCODE) data for regulatory function of variants in 6p12.2 using the UCSC Genome Browser. The plot represents 6p12.2 within a 20-kb window centered on IL-17 genes region. Tracks (from top to bottom) in each of the plots are Genome Base Position, Chromosome Bands, UCSC Genes, Human messenger RNAs from GenBank, Human expressed sequence tag (ESTs) That Have Been Spliced, ENCODE Enhancer and Promoter-Associated Histone Mark (H3K4Me1) on 8 Cell Lines, ENCODE Promoter-Associated Histone Mark (H3K4Me3) on 9 Cell Lines, ENCODE Digital DNaseI Hypersensitivity Clusters, ENCODE Transcription Factor ChIP-seq, ENCODE Chromatin State Segmentation by Hidden Markov Model (HMM) from Broad Institute (bright red, active promoter; light red, weak promoter; purple, inactive/poised promoter; orange, strong enhancer; yellow, weak/poised enhancer; blue, insulator; dark green, transcriptional transition/elongation; light green, weak transcribed; gray, polycomb-repressed; light gray, heterochromatin/low signal/repetitive/copy number variation), Simple Nucleotide Polymorphisms (dbSNP build 130), Linkage Disequilibrium for the Yoruba (YRI) from Phased Genotypes, Linkage Disequilibrium for the CEPH (CEU) from Phased Genotypes and LD for the Han Chinese + Japanese from Tokyo (JPT+CHB) from Phased Genotypes.

Supplementary Table 1. Characteristics of the included articles.

Pubmed ID	First author	year	Disease	Ethnicity	Study design	Case	Control	Sample size	Gene name	Variant	Allelic
16630936	Kawaguchi, M	2006	Asthma	Asian	Case-control study	432	435	867	IL17F	rs1889570 C/T	C/T
16630936	Kawaguchi, M	2006	Asthma	Asian	Case-control study				IL17F	rs1266828 G/A	G/A
16630936	Kawaguchi, M	2006	Asthma	Asian	Case-control study				IL17F	rs7771511 C/T	C/T
16630936	Kawaguchi, M	2006	Asthma	Asian	Case-control study				IL17F	rs766748 C/T	C/T
16630936	Kawaguchi, M	2006	Asthma	Asian	Case-control study				IL17F	rs763780 T/C	T/C
17912466	Arisawa, T	2007	Functional dyspepsia (FD)	Asian	Case-control study	90	188	278	IL17A	rs2275913 G/A	G/A
17912466	Arisawa, T	2007	Functional dyspepsia (FD)	Asian	Case-control study				IL17A	rs2275913 G/A	G/A
17912466	Arisawa, T	2007	Functional dyspepsia (FD)	Asian	Case-control study				IL17A	rs2275913 G/A	G/A
17912466	Arisawa, T	2007	Functional dyspepsia (FD)	Asian	Case-control study				IL17F	rs763780 T/C	T/C
17912466	Arisawa, T	2007	Functional dyspepsia (FD)	Asian	Case-control study				IL17F	rs763780 T/C	T/C
17912466	Arisawa, T	2007	Functional dyspepsia (FD)	Asian	Case-control study				IL17F	rs763780 T/C	T/C
18088064	Seiderer, J	2008	Inflammatory Bowel Disease (IBD)	Caucasian	Case-control study	499	967	1466	IL17F	rs763780 T/C	T/C
18088064	Seiderer, J	2008	Inflammatory Bowel Disease (IBD)	Caucasian	Case-control study	216	967	1183	IL17F	rs763780 T/C	T/C
18769923	Jang, W. C	2008	Behcet's disease (BD)	Asian	Case-control study	99	114	213	IL17F	rs2397084 T/C	T/C
18769923	Jang, W. C	2008	Behcet's disease (BD)	Asian	Case-control study				IL17F	rs11465553 C/T	C/T
18769923	Jang, W. C	2008	Behcet's disease (BD)	Asian	Case-control study				IL17F	rs763780 T/C	T/C
18774769	Metzger, K	2008	Chronic fatigue syndrome (CFS)	Caucasian	Case-control study	89	56	145	IL17F	rs763780 T/C	T/C
19019635	Shibata, S	2009	Psoriasis vulgaris (PsV)	Asian	Case-control study	153	103	256	IL17F	rs763780 T/C	T/C

19019635	Shibata, S	2009	Atopic dermatitis (AD)	Asian	Case-control study				IL17F	rs763780 T/C	T/C
19118269	Jung, J. S	2009	Asthma	Asian	Case-control study	954	265	1219	IL17RB	rs28385732 G/A	G/A
19118269	Jung, J. S	2009	Asthma	Asian	Case-control study				IL17RB	rs3733072 G/A	G/A
19118269	Jung, J. S	2009	Asthma	Asian	Case-control study				IL17RB	rs3733075 C/T	C/T
19118269	Jung, J. S	2009	Asthma	Asian	Case-control study				IL17RB	rs6766099 C/T	C/T
19118269	Jung, J. S	2009	Asthma	Asian	Case-control study				IL17RB	rs1043261 C/T	C/T
19118269	Jung, J. S	2009	Asthma	Asian	Case-control study				IL17RB	rs3017 A/G	A/G
19208686	Nordang, G. B	2009	Rheumatoid arthritis (RA)	Caucasian	Case-control study	914	899	1813	IL17A	rs4711998 A/G	A/G
19208686	Nordang, G. B	2009	Rheumatoid arthritis (RA)	Caucasian	Case-control study	937	928	1865	IL17A	rs3819024 A/G	A/G
19208686	Nordang, G. B	2009	Rheumatoid arthritis (RA)	Caucasian	Case-control study	938	920	1858	IL17A	rs2275913 G/A	G/A
19208686	Nordang, G. B	2009	Rheumatoid arthritis (RA)	Caucasian	Case-control study	921	920	1841	IL17A	rs7747909 G/A	G/A
19208686	Nordang, G. B	2009	Rheumatoid arthritis (RA)	Caucasian	Case-control study	936	923	1859	IL17A	rs8193036 C/T	C/T
19208686	Nordang, G. B	2009	Rheumatoid arthritis (RA)	Caucasian	Case-control study	580	504	1084	IL17A	rs2275913 G/A	G/A
19210369	Wang, J. Y	2009	Asthma	Asian	Case-control study	481	546	1027	IL17A	rs4711998 A/G	A/G
19210369	Wang, J. Y	2009	Asthma	Asian	Case-control study				IL17A	rs8193036 C/T	C/T
19210369	Wang, J. Y	2009	Asthma	Asian	Case-control study				IL17A	rs3819024 A/G	A/G
19210369	Wang, J. Y	2009	Asthma	Asian	Case-control study				IL17A	rs2275913 G/A	G/A
19210369	Wang, J. Y	2009	Asthma	Asian	Case-control study				IL17A	rs3819025 G/A	G/A
19210369	Wang, J. Y	2009	Asthma	Asian	Case-control study				IL17A	rs8193038 A/G	A/G
19210369	Wang, J. Y	2009	Asthma	Asian	Case-control study				IL17A	rs3804513 A/T	A/T
19210369	Wang, J. Y	2009	Asthma	Asian	Case-control study				IL17A	rs1974226 C/T	C/T
19210369	Wang, J. Y	2009	Asthma	Asian	Case-control study				IL17A	rs3748067 C/T	C/T
19210369	Wang, J. Y	2009	Asthma	Asian	Case-control study				IL17A	rs8193036 C/T	C/T
19263269	Chen, B	2009	Inflammatory Bowel Disease (IBD)	Asian	Case-control study	134	373	507	IL17F	rs763780 T/C	T/C
19263269	Chen, B	2009	Inflammatory Bowel Disease (IBD)	Asian	Case-control study	148	373	521	IL17F	rs763780 T/C	T/C
19414056	Shibata, T	2009	Gastric Cancer (GC)	Asian	Case-control study	278	524	802	IL17A	rs2275913 G/A	G/A
19414056	Shibata, T	2009	Gastric Cancer (GC)	Asian	Case-control study				IL17F	rs763780 T/C	T/C
19838108	Pei, F	2009	Myocardial infarction (MI)	Asian	Case-control study	513	477	990	IL17F	rs763780 T/C	T/C
19904747	Wu, X	2010	Gastric Cancer (GC)	Asian	Case-control study	962	787	1749	IL17A	rs2275913 G/A	G/A

19904747	Wu, X	2010	Gastric Cancer (GC)	Asian	Case-control study				IL17F	rs763780 T/C	T/C
20437253	Chen, J	2010	Asthma	Asian	Case-control study	168	205	373	IL17A	rs2275913 G/A	G/A
20437253	Chen, J	2010	Bronchiolitis	Asian	Case-control study	144	205	349	IL17A	rs2275913 G/A	G/A
20618772	Paradowska G, A	2010	Rheumatoid arthritis (RA)	Caucasian	Case-control study	220	106	326	IL17F	rs763780 T/C	T/C
20618772	Paradowska G, A	2010	Rheumatoid arthritis (RA)	Caucasian	Case-control study				IL17F	rs2397084 T/C	T/C
20620187	Shu, Q	2010	Vogt–Koyanagi–Harada (VKH) syndrome	Asian	Case-control study	385	412	797	IL17F	rs763780 T/C	T/C
20620187	Shu, Q	2010	Behcet's disease (BD)	Asian	Case-control study	362	412	774	IL17F	rs763780 T/C	T/C
20620187	Shu, Q	2010	Vogt–Koyanagi–Harada (VKH) syndrome	Asian	Case-control study				IL17A	rs2275913 G/A	G/A
20620187	Shu, Q	2010	Behcet's disease (BD)	Asian	Case-control study				IL17A	rs2275913 G/A	G/A
21062626	Zhang, X	2010	Coronary artery disease (CAD)	Asian	Case-control study	1031	935	1966	IL17A	rs4711998 A/G	A/G
21062626	Zhang, X	2010	Coronary artery disease (CAD)	Asian	Case-control study				IL17A	rs3819024 A/G	A/G
21062626	Zhang, X	2010	Coronary artery disease (CAD)	Asian	Case-control study				IL17A	rs2275913 G/A	G/A
21062626	Zhang, X	2011	Coronary artery disease (CAD)	Asian	Case-control study				IL17A	rs8193037 G/A	G/A
21062626	Zhang, X	2011	Coronary artery disease (CAD)	Asian	Case-control study				IL17A	rs3819025 G/A	G/A
21196754	Jin, E. H	2011	Asthma	Asian	Case-control study	424	548	972	IL17F	rs1889570 C/T	C/T
21196754	Jin, E. H	2011	Asthma	Asian	Case-control study				IL17F	rs763780 T/C	T/C
21615796	Saitoh, T	2011	Immune thrombocytopenia (ITP)	Asian	Case-control study	102	188	290	IL17F	rs763780 T/C	T/C
21672939	Kim, S. W	2011	Inflammatory Bowel Disease (IBD)	Asian	Case-control study	201	258	459	IL17A	rs8193036 C/T	C/T
21672939	Kim, S. W	2011	Inflammatory Bowel Disease (IBD)	Asian	Case-control study	268	258	526	IL17A	rs8193036 C/T	C/T
21672939	Kim, S. W	2011	Inflammatory Bowel Disease (IBD)	Asian	Case-control study				IL17A	rs2275913 G/A	G/A
21672939	Kim, S. W	2011	Inflammatory Bowel	Asian	Case-control study				IL17A	rs2275913 G/A	G/A

			Disease (IBD)								
21672939	Kim, S. W	2011	Inflammatory Bowel Disease (IBD)	Asian	Case-control study				IL17A	rs3819025 G/A	G/A
21672939	Kim, S. W	2011	Inflammatory Bowel Disease (IBD)	Asian	Case-control study				IL17A	rs3819025 G/A	G/A
21737283	Jiang, N	2011	Brain arteriovenous malformation (BAVM)	Asian	Case-control study	53	120	173	IL17A	rs2275913 G/A	G/A
22118860	Wang, H	2012	Neuromyelitisoptica (NMO)	Asian	Case-control study	52	131	183	IL17A	rs2275913 G/A	G/A
22118860	Wang, H	2012	Multiple sclerosis (MS)	Asian	Case-control study	69	131	200	IL17A	rs2275913 G/A	G/A
22118860	Wang, H	2012	Neuromyelitisoptica (NMO)	Asian	Case-control study				IL17F	rs763780 T/C	T/C
22118860	Wang, H	2012	Multiple sclerosis (MS)	Asian	Case-control study				IL17F	rs763780 T/C	T/C
22123380	Kohyama, K	2011	Aspirin-exacerbated respiratory disease (AERD)	Asian	Case-control study	95	100	195	IL17A	rs8193036 C/T	C/T
22123380	Kohyama, K	2011	Aspirin tolerant asthma (ATA)	Asian	Case-control study	300	100	400	IL17A	rs8193036 C/T	C/T
22461912	Wang, L	2012	Breast cancer	Asian	Case-control study	491	502	993	IL17A	rs2275913 G/A	G/A
22461912	Wang, L	2012	Breast cancer	Asian	Case-control study				IL17A	rs3819025 G/A	G/A
22461912	Wang, L	2012	Breast cancer	Asian	Case-control study				IL17A	rs3748067 C/T	C/T
22461912	Wang, L	2012	Breast cancer	Asian	Case-control study				IL17F	rs763780 T/C	T/C
22461912	Wang, L	2012	Breast cancer	Asian	Case-control study				IL17F	rs7771511 C/T	C/T
22461912	Wang, L	2012	Breast cancer	Asian	Case-control study				IL17F	rs12203582 G/A	G/A
22461912	Wang, L	2012	Breast cancer	Asian	Case-control study				IL17F	rs9382084 G/T	G/T
22461912	Wang, L	2012	Breast cancer	Asian	Case-control study				IL17F	rs1266828 G/A	G/A
22483685	Kim, E. S	2012	Behcet's disease (BD)	Asian	Case-control study	141	259	400	IL17A	rs4711998 A/G	A/G
22483685	Kim, E. S	2012	Behcet's disease (BD)	Asian	Case-control study				IL17A	rs8193036 C/T	C/T
22483685	Kim, E. S	2012	Behcet's disease (BD)	Asian	Case-control study				IL17A	rs9791323 A/C	A/C
22483685	Kim, E. S	2012	Behcet's disease (BD)	Asian	Case-control study				IL17A	rs3819024 A/G	A/G
22483685	Kim, E. S	2012	Behcet's disease (BD)	Asian	Case-control study				IL17A	rs2275913 G/A	G/A
22483685	Kim, E. S	2012	Behcet's disease (BD)	Asian	Case-control study				IL17A	rs8193037 G/A	G/A
22507625	Wang, M	2012	Allergic rhinitis (AR)	Asian	Case-control study	279	281	560	IL17A	rs4711998 A/G	A/G
22507625	Wang, M	2012	Allergic rhinitis (AR)	Asian	Case-control study				IL17A	rs8193036 C/T	C/T
22507625	Wang, M	2012	Allergic rhinitis (AR)	Asian	Case-control study				IL17A	rs3819024 A/G	A/G
22507625	Wang, M	2012	Allergic rhinitis (AR)	Asian	Case-control study				IL17A	rs3819025 G/A	G/A

22507625	Wang, M	2012	Allergic rhinitis (AR)	Asian	Case-control study				IL17A	rs3804513 A/T	A/T
22507625	Wang, M	2012	Allergic rhinitis (AR)	Asian	Case-control study				IL17A	rs1974226 C/T	C/T
22507625	Wang, M	2012	Allergic rhinitis (AR)	Asian	Case-control study				IL17F	rs13209590 A/G	A/G
22507625	Wang, M	2012	Allergic rhinitis (AR)	Asian	Case-control study				IL17F	rs7771466 C/A	C/A
22507625	Wang, M	2012	Allergic rhinitis (AR)	Asian	Case-control study				IL17F	rs1266828 G/A	G/A
22507625	Wang, M	2012	Allergic rhinitis (AR)	Asian	Case-control study				IL17F	rs669161 A/G	A/G
22507625	Wang, M	2012	Allergic rhinitis (AR)	Asian	Case-control study	197	281	478	IL17A	rs4711998 A/G	A/G
22507625	Wang, M	2012	Allergic rhinitis (AR)	Asian	Case-control study				IL17A	rs8193036 C/T	C/T
22507625	Wang, M	2012	Allergic rhinitis (AR)	Asian	Case-control study				IL17A	rs3819024 A/G	A/G
22507625	Wang, M	2012	Allergic rhinitis (AR)	Asian	Case-control study				IL17A	rs3819025 G/A	G/A
22507625	Wang, M	2012	Allergic rhinitis (AR)	Asian	Case-control study				IL17A	rs3804513 A/T	A/T
22507625	Wang, M	2012	Allergic rhinitis (AR)	Asian	Case-control study				IL17A	rs1974226 C/T	C/T
22507625	Wang, M	2012	Allergic rhinitis (AR)	Asian	Case-control study				IL17F	rs13209590 A/G	A/G
22507625	Wang, M	2012	Allergic rhinitis (AR)	Asian	Case-control study				IL17F	rs7771466 C/A	C/A
22507625	Wang, M	2012	Allergic rhinitis (AR)	Asian	Case-control study				IL17F	rs1266828 G/A	G/A
22507625	Wang, M	2012	Allergic rhinitis (AR)	Asian	Case-control study				IL17F	rs669161 A/G	A/G
22537748	Arisawa, T	2012	Gastric Cancer (GC)	Asian	Case-control study	199	587	786	IL17A	rs2275913 G/A	G/A
22537748	Arisawa, T	2012	Gastric Cancer (GC)	Asian	Case-control study	134	587	721	IL17A	rs3748067 C/T	C/T
22537748	Arisawa, T	2012	Gastric Cancer (GC)	Asian	Case-control study	337	587	924	IL17A	rs2275913 G/A	G/A
22537748	Arisawa, T	2012	Gastric Cancer (GC)	Asian	Case-control study				IL17A	rs3748067 C/T	C/T
22559912	Popovic K, D	2013	Primary antiphospholipid syndrome (PAPS)	Caucasian	Case-control study	50	50	100	IL17A	rs2275913 G/A	G/A
22692973	Zhou, B	2013	Bladder Cancer	Asian	Case-control study	301	446	747	IL17A	rs2275913 G/A	G/A
22692973	Zhou, B	2013	Bladder Cancer	Asian	Case-control study				IL17F	rs763780 T/C	T/C
22812194	Qian, F	2012	Asthma	Asian	Case-control study	318	352	670	IL17F	rs13209590 A/G	A/G
22812194	Qian, F	2012	Asthma	Asian	Case-control study				IL17F	rs763780 T/C	T/C
22816799	Yan, N	2012	Autoimmune thyroid diseases (AITD)	Asian	Case-control study	326	224	550	IL17F	rs763780 T/C	T/C
22816799	Yan, N	2012	Autoimmune thyroid diseases (AITD)	Asian	Case-control study	326	224	550	IL17A	rs2275913 G/A	G/A
22816799	Yan, N	2012	Autoimmune thyroid diseases (AITD)	Asian	Case-control study				IL17A	rs8193037 G/A	G/A

22816799	Yan, N	2012	Autoimmune thyroid diseases (AITD)	Asian	Case-control study				IL17A	rs3819025 G/A	G/A
22816799	Yan, N	2012	Autoimmune thyroid diseases (AITD)	Asian	Case-control study	182	224	406	IL17F	rs763780 T/C	T/C
22816799	Yan, N	2012	Autoimmune thyroid diseases (AITD)	Asian	Case-control study				IL17A	rs2275913 G/A	G/A
22816799	Yan, N	2012	Autoimmune thyroid diseases (AITD)	Asian	Case-control study				IL17A	rs8193037 G/A	G/A
22816799	Yan, N	2012	Autoimmune thyroid diseases (AITD)	Asian	Case-control study				IL17A	rs3819025 G/A	G/A
22999050	Zhou, S	2012	Adolescent idiopathic scoliosis (AIS)	Asian	Case-control study	529	512	1041	IL-17RC	rs708567 C/T	C/T
23012664	Hayashi, R	2012	Gastro-duodenal ulcer (UL)	Asian	Case-control study	127	363	490	IL17A	rs2275913 G/A	G/A
23012664	Hayashi, R	2012	Gastro-duodenal ulcer (UL)	Asian	Case-control study	58	363	421	IL17A	rs2275913 G/A	G/A
23012664	Hayashi, R	2012	Gastro-duodenal ulcer (UL)	Asian	Case-control study	127	363	490	IL17F	rs763780 T/C	T/C
23012664	Hayashi, R	2012	Gastro-duodenal ulcer (UL)	Asian	Case-control study	58	363	421	IL17F	rs763780 T/C	T/C
23049595	Quan, Y	2012	Cervical cancer	Asian	Case-control study	311	463	774	IL17A	rs2275913 G/A	G/A
23049595	Quan, Y	2012	Cervical cancer	Asian	Case-control study				IL17F	rs763780 T/C	T/C
23111159	Guo, T	2013	Autoimmune thyroid diseases (AITD)	Asian	Case-control study	757	741	1498	IL17F	rs761167 C/T	C/T
23111159	Guo, T	2013	Autoimmune thyroid diseases (AITD)	Asian	Case-control study				IL17F	rs9463772 C/T	C/T
23111159	Guo, T	2013	Autoimmune thyroid diseases (AITD)	Asian	Case-control study				IL17F	rs2397084 T/C	T/C
23111159	Guo, T	2013	Autoimmune thyroid diseases (AITD)	Asian	Case-control study				IL17F	rs763780 T/C	T/C
17828618	Arisawa T	2007	Inflammatory Bowel Disease (IBD)	Asian	Case-control study	111	248	359	IL17A	rs2275913 G/A	G/A
17828618	Arisawa T	2007	Inflammatory Bowel Disease (IBD)	Asian	Case-control study				IL17F	rs763780 T/C	T/C
23131423	Rasouli, M	2013	Brucellosis	Caucasian	Case-control study	176	84	260	IL17A	rs4711998 A/G	A/G
23131423	Rasouli, M	2013	Brucellosis	Caucasian	Case-control study				IL17A	rs8193036 C/T	C/T

23131423	Rasouli, M	2013	Brucellosis	Caucasian	Case-control study				IL17A	rs3819024 A/G	A/G
23131423	Rasouli, M	2013	Brucellosis	Caucasian	Case-control study				IL17A	rs2275913 G/A	G/A
23131423	Rasouli, M	2013	Brucellosis	Caucasian	Case-control study				IL17A	rs3819025 G/A	G/A
23131423	Rasouli, M	2013	Brucellosis	Caucasian	Case-control study				IL17A	rs8193038 A/G	A/G
23131423	Rasouli, M	2013	Brucellosis	Caucasian	Case-control study				IL17A	rs3804513 A/T	A/T
23131423	Rasouli, M	2013	Brucellosis	Caucasian	Case-control study				IL17A	rs1974226 C/T	C/T
23131423	Rasouli, M	2013	Brucellosis	Caucasian	Case-control study				IL17A	rs3748067 C/T	C/T
23137879	Saraiva, A. M	2012	Periodontitis	Mixed	Case-control study	45	72	117	IL17A	rs2275913 G/A	G/A
23137879	Saraiva, A. M	2012	Periodontitis	Mixed	Case-control study	85	72	157	IL17A	rs2275913 G/A	G/A
23137879	Saraiva, A. M	2012	Periodontitis	Mixed	Case-control study				IL17F	rs763780 T/C	T/C
23137879	Saraiva, A. M	2012	Periodontitis	Mixed	Case-control study				IL17F	rs763780 T/C	T/C
23219503	Peng, R	2013	Tuberculosis (TB)	Asian	Case-control study	596	622	1218	IL17F	rs1889570 C/T	C/T
23219503	Peng, R	2013	Tuberculosis (TB)	Asian	Case-control study	176	622	798	IL17F	rs1889570 C/T	C/T
23219503	Peng, R	2013	Tuberculosis (TB)	Asian	Case-control study				IL17A	rs2275913 G/A	G/A
23219503	Peng, R	2013	Tuberculosis (TB)	Asian	Case-control study				IL17A	rs2275913 G/A	G/A
23219503	Peng, R	2013	Tuberculosis (TB)	Asian	Case-control study				IL17F	rs763780 T/C	T/C
23219503	Peng, R	2013	Tuberculosis (TB)	Asian	Case-control study				IL17F	rs763780 T/C	T/C
23280722	Li, N	2014	Hepatitis B Virus (HBV)	Asian	Case-control study	395	75	470	IL17A	rs8193036 C/T	C/T
23280722	Li, N	2014	Hepatitis B Virus (HBV)	Asian	Case-control study				IL17A	rs2275913 G/A	G/A
23280722	Li, N	2014	Hepatitis B Virus (HBV)	Asian	Case-control study	395	174	569	IL17A	rs8193036 C/T	C/T
23280722	Li, N	2014	Hepatitis B Virus (HBV)	Asian	Case-control study				IL17A	rs2275913 G/A	G/A
23280722	Li, N	2014	Hepatitis B Virus (HBV)	Asian	Case-control study	75	174	249	IL17A	rs8193036 C/T	C/T
23280722	Li, N	2014	Hepatitis B Virus (HBV)	Asian	Case-control study				IL17A	rs2275913 G/A	G/A
23280722	Li, N	2014	Hepatitis B Virus (HBV)	Asian	Case-control study	134	174	308	IL17A	rs8193036 C/T	C/T
23280722	Li, N	2014	Hepatitis B Virus (HBV)	Asian	Case-control study				IL17A	rs2275913 G/A	G/A
23280722	Li, N	2014	Hepatitis B Virus (HBV)	Asian	Case-control study	125	174	299	IL17A	rs8193036 C/T	C/T
23280722	Li, N	2014	Hepatitis B Virus (HBV)	Asian	Case-control study				IL17A	rs2275913 G/A	G/A
23280722	Li, N	2014	Hepatitis B Virus (HBV)	Asian	Case-control study	136	174	310	IL17A	rs8193036 C/T	C/T
23280722	Li, N	2014	Hepatitis B Virus (HBV)	Asian	Case-control study				IL17A	rs2275913 G/A	G/A
23280722	Li, N	2014	Hepatocellular carcinoma (HCC)	Asian	Case-control study	136	134	270	IL17A	rs8193036 C/T	C/T

23280722	Li, N	2014	Hepatocellular carcinoma (HCC)	Asian	Case-control study				IL17A	rs2275913 G/A	G/A
23280722	Li, N	2014	Hepatocellular carcinoma (HCC)	Asian	Case-control study	136	125	261	IL17A	rs8193036 C/T	C/T
23280722	Li, N	2014	Hepatocellular carcinoma (HCC)	Asian	Case-control study				IL17A	rs2275913 G/A	G/A
23280722	Li, N	2014	Chronic hepatitis (CH)	Asian	Case-control study	134	125	259	IL17A	rs8193036 C/T	C/T
23280722	Li, N	2014	Chronic hepatitis (CH)	Asian	Case-control study				IL17A	rs2275913 G/A	G/A
23304063	Corrêa, J. D	2012	Periodontitis	Mixed	Case-control study	30	30	60	IL17A	rs2275913 G/A	G/A
23304063	Corrêa, J. D	2012	Periodontitis	Mixed	Case-control study				IL17F	rs763780 T/C	T/C
23323524	Kadkhodazadeh, M	2013	Periodontitis	Caucasian	Cross-Sectional Study	75	84	159	IL17A	rs10484879 C/A	C/A
23323524	Kadkhodazadeh, M	2013	peri-implantitis (PI)	Caucasian	Cross-Sectional Study	38	84	122	IL17A	rs10484879 C/A	C/A
23376081	Peng, Y	2013	Dilated cardiomyopathy (DCM)	Asian	Case-control study	288	421	709	IL17A	rs2275913 G/A	G/A
23376081	Peng, Y	2013	Dilated cardiomyopathy (DCM)	Asian	Case-control study				IL17F	rs763780 T/C	T/C
23485736	Nie, K	2013	Parkinson’s Disease	Asian	Case-control study	302	294	596	IL17A	rs8193036 C/T	C/T
23485736	Nie, K	2013	Parkinson’s Disease	Asian	Case-control study				IL17A	rs2275913 G/A	G/A
23778030	Ocejo Vinyals, J. G	2013	Tuberculosis (TB)	Caucasian	Case-control study	192	266	458	IL17A	rs2275913 G/A	G/A
23852838	Kadkhodazadeh, M	2013	Periodontitis	Caucasian	Case-control study	73	83	156	IL-17R	rs879576 G/A	G/A
23852838	Kadkhodazadeh, M	2013	Periodontitis	Caucasian	Case-control study	37	83	120	IL-17R	rs879576 G/A	G/A
23876429	Abhimanyu	2013	Tuberculosis (TB)	Caucasian	Case-control study	165	130	295	IL17A	rs2275913 G/A	G/A
23876429	Abhimanyu	2013	Tuberculosis (TB)	Caucasian	Case-control study	50	130	180	IL17A	rs2275913 G/A	G/A
23876429	Abhimanyu	2013	Tuberculosis (TB)	Caucasian	Case-control study				IL17F	rs763780 T/C	T/C
23876429	Abhimanyu	2013	Tuberculosis (TB)	Caucasian	Case-control study				IL17F	rs763780 T/C	T/C
23895419	Yin, J	2013	Esophageal cancer (EC)	Asian	Case-control study	380	380	760	IL17A	rs2275913 G/A	G/A
23895419	Yin, J	2013	Esophageal cancer (EC)	Asian	Case-control study				IL17A	rs3819024 A/G	A/G
23895419	Yin, J	2013	Esophageal cancer (EC)	Asian	Case-control study				IL17A	rs3819025 G/A	G/A
23895419	Yin, J	2013	Esophageal cancer (EC)	Asian	Case-control study				IL17A	rs4711998 A/G	A/G
23895419	Yin, J	2013	Esophageal cancer (EC)	Asian	Case-control study				IL17A	rs8193036 C/T	C/T

23895419	Yin, J	2013	Esophageal cancer (EC)	Asian	Case-control study				IL17A	rs8193037 G/A	G/A
24039363	Rafiei, A	2013	Gastric Cancer (GC)	Caucasian	Case-control study	161	171	332	IL17A	rs2275913 G/A	G/A
24096234	Han, L	2014	Osteoarthritis (OA)	Asian	Case-control study	302	300	602	IL17A	rs2275913 G/A	G/A
24096234	Han, L	2014	Osteoarthritis (OA)	Asian	Case-control study				IL17F	rs763780 T/C	T/C
24164796	Chaitanya, V. S	2014	Leprosy	Other	Case-control study	53	84	137	IL17F	rs763780 T/C	T/C
24164796	Chaitanya, V. S	2014	Leprosy	Other	Case-control study	87	84	171	IL17F	rs763780 T/C	T/C
24218334	Zhang, X	2014	Gastric Cancer (GC)	Asian	Case-control study	182	512	694	IL17A	rs2275913 G/A	G/A
24218334	Zhang, X	2014	Gastric Cancer (GC)	Asian	Case-control study	78	512	590	IL17A	rs2275913 G/A	G/A
24218334	Zhang, X	2014	Gastric Cancer (GC)	Asian	Case-control study				IL17A	rs3748067 C/T	C/T
24218334	Zhang, X	2014	Gastric Cancer (GC)	Asian	Case-control study				IL17A	rs3748067 C/T	C/T
24218334	Zhang, X	2014	Gastric Cancer (GC)	Asian	Case-control study	260	512	772	IL17F	rs763780 T/C	T/C
24315816	Qinghai, Z	2014	Gastric Cancer (GC)	Asian	Case-control study	206	550	756	IL17A	rs2275913 G/A	G/A
24315816	Qinghai, Z	2014	Gastric Cancer (GC)	Asian	Case-control study	87	550	637	IL17A	rs2275913 G/A	G/A
24315816	Qinghai, Z	2014	Gastric Cancer (GC)	Asian	Case-control study				IL17A	rs3748067 C/T	C/T
24315816	Qinghai, Z	2014	Gastric Cancer (GC)	Asian	Case-control study				IL17A	rs3748067 C/T	C/T
24315816	Qinghai, Z	2014	Gastric Cancer (GC)	Asian	Case-control study				IL17F	rs9382084 G/T	G/T
24315816	Qinghai, Z	2014	Gastric Cancer (GC)	Asian	Case-control study				IL17F	rs9382084 G/T	G/T
24315816	Qinghai, Z	2014	Gastric Cancer (GC)	Asian	Case-control study	293	550	843	IL17A	rs3819025 G/A	G/A
24315816	Qinghai, Z	2014	Gastric Cancer (GC)	Asian	Case-control study				IL17F	rs763780 T/C	T/C
24315816	Qinghai, Z	2014	Gastric Cancer (GC)	Asian	Case-control study				IL17F	rs12203582 G/A	G/A
24393079	Maalmi, H	2014	Asthma	Caucasian	Case-control study	47	171	218	IL17A	rs2275913 G/A	G/A
24393079	Maalmi, H	2014	Asthma	Caucasian	Case-control study				IL17F	rs763780 T/C	T/C
24393079	Maalmi, H	2014	Asthma	Caucasian	Case-control study				IL17F	rs2397084 T/C	T/C
24393079	Maalmi, H	2014	Asthma	Caucasian	Case-control study	147	171	318	IL17A	rs2275913 G/A	G/A
24393079	Maalmi, H	2014	Asthma	Caucasian	Case-control study				IL17F	rs763780 T/C	T/C
24393079	Maalmi, H	2014	Asthma	Caucasian	Case-control study				IL17F	rs2397084 T/C	T/C
24440568	Omrane, I	2014	Colorectal cance (CRC)	Caucasian	Case-control study	100	137	237	IL17F	rs763780 T/C	T/C
24548421	Kaabachi, W	2014	Non-small cell lung cancer (NSCLC)	Caucasian	Case-control study	239	258	497	IL17F	rs763780 T/C	T/C
24548421	Kaabachi, W	2014	Non-small cell lung cancer (NSCLC)	Caucasian	Case-control study				IL17F	rs2397084 T/C	T/C

24548421	Kaabachi, W	2014	Non-small cell lung cancer (NSCLC)	Caucasian	Case-control study				IL17A	rs2275913 G/A	G/A
24690400	Karakas Celik, S	2014	Subacute sclerosing panencephalitis (SSPE)	Caucasian	Case-control study	54	81	135	IL17F	rs763780 T/C	T/C
24690400	Karakas Celik, S	2014	Subacute sclerosing panencephalitis (SSPE)	Caucasian	Case-control study				IL17F	rs2397084 T/C	T/C
24690400	Karakas Celik, S	2014	Subacute sclerosing panencephalitis (SSPE)	Caucasian	Case-control study				IL17A	rs2275913 G/A	G/A
24699997	Omrane, I	2014	Colorectal cance (CRC)	Caucasian	Case-control study	102	139	241	IL17A	rs2275913 G/A	G/A
24793548	Wróbel, T	2014	Leukemia	Caucasian	Case-control study	62	125	187	IL17A	rs2275913 G/A	G/A
24793548	Wróbel, T	2014	Leukemia	Caucasian	Case-control study				IL17F	rs763780 T/C	T/C
24857622	Wang, S	2014	Multiple sclerosis (MS)	Asian	Case-control study	622	743	1365	IL17A	rs2275913 G/A	G/A
24857622	Wang, S	2014	Multiple sclerosis (MS)	Asian	Case-control study				IL17F	rs763780 T/C	T/C
24893702	Ren, Z	2014	Gastric Cancer (GC)	Asian	Case-control study	243	476	719	IL17A	rs2275913 G/A	G/A
24893702	Ren, Z	2014	Gastric Cancer (GC)	Asian	Case-control study				IL17A	rs3819024 A/G	A/G
24893702	Ren, Z	2014	Gastric Cancer (GC)	Asian	Case-control study				IL17A	rs3819025 G/A	G/A
24893702	Ren, Z	2014	Gastric Cancer (GC)	Asian	Case-control study				IL17A	rs4711998 A/G	A/G
24893702	Ren, Z	2014	Gastric Cancer (GC)	Asian	Case-control study				IL17A	rs8193036 C/T	C/T
24893702	Ren, Z	2014	Gastric Cancer (GC)	Asian	Case-control study				IL17A	rs8193037 G/A	G/A
24975966	Bahadori, M	2014	Recurrent Miscarriage (RM)	Caucasian	Case-control study	85	104	189	IL17A	rs2275913 G/A	G/A
25012243	Wang, N	2014	Gastric Cancer (GC)	Asian	Case-control study	462	462	924	IL17A	rs2275913 G/A	G/A
25012243	Wang, N	2014	Gastric Cancer (GC)	Asian	Case-control study				IL17A	rs3748067 C/T	C/T
25012243	Wang, N	2014	Gastric Cancer (GC)	Asian	Case-control study				IL17F	rs763780 T/C	T/C
25159155	Paradowska G, A	2015	Mixed connective tissue disease (MCTD)	Caucasian	Case-control study	66	106	172	IL17F	rs763780 T/C	T/C
25216863	Zhu, L	2014	Colorectal cance (CRC)	Asian	Case-control study	214	627	841	IL17	rs6973569 G/A	G/A
25216863	Zhu, L	2014	Colorectal cance (CRC)	Asian	Case-control study	387	627	1014	IL17	rs6973569 G/A	G/A
25223230	Narbutt, J	2015	Atopic dermatitis (AD)	Caucasian	Case-control study	166	160	326	IL17A	rs2275913 G/A	G/A
25227691	Najafi, S	2014	Recurrent Miscarriage (RM)	Caucasian	Case-control study	85	85	170	IL17F	rs763780 T/C	T/C
25227691	Najafi, S	2014	Recurrent Miscarriage (RM)	Caucasian	Case-control study				IL17A	rs2275913 G/A	G/A
25289477	Cheng, S	2015	Non-small cell lung cancer (NSCLC)	Asian	Case-control study	113	366	479	IL17A	rs3819024 A/G	A/G

25289477	Cheng, S	2015	Non-small cell lung cancer (NSCLC)	Asian	Case-control study	171	366	537	IL17A	rs3819024 A/G	A/G
25289477	Cheng, S	2015	Non-small cell lung cancer (NSCLC)	Asian	Case-control study	38	366	404	IL17A	rs3819024 A/G	A/G
25289477	Cheng, S	2015	Non-small cell lung cancer (NSCLC)	Asian	Case-control study	113	366	479	IL17A	rs8193037 G/A	G/A
25289477	Cheng, S	2015	Non-small cell lung cancer (NSCLC)	Asian	Case-control study	171	366	537	IL17A	rs8193037 G/A	G/A
25289477	Cheng, S	2015	Non-small cell lung cancer (NSCLC)	Asian	Case-control study	38	366	404	IL17A	rs8193037 G/A	G/A
25296730	Ma, M	2015	Colorectal cance (CRC)	Asian	Case-control study	1000	354	1354	IL17F	rs763780 T/C	T/C
25296730	Ma, M	2015	Colorectal cance (CRC)	Asian	Case-control study				IL17F	rs2397084 T/C	T/C
25374205	Chen, Y	2014	Silicosis	Asian	Case-control study	113	116	229	IL17A	A-832G A/G	A/G
25374205	Chen, Y	2014	Silicosis	Asian	Case-control study				IL17F	rs763780 T/C	T/C
25387578	Bogunia Kubik, K	2015	Rheumatoid arthritis (RA)	Caucasian	Case-control study	89	125	214	IL17A	rs2275913 G/A	G/A
25387578	Bogunia Kubik, K	2015	Rheumatoid arthritis (RA)	Caucasian	Case-control study				IL17F	rs763780 T/C	T/C
25429834	Xi, X. E	2015	Hepatocellular carcinoma (HCC)	Asian	Case-control study	155	171	326	IL17A	rs2275913 G/A	G/A
25429834	Xi, X. E	2015	Hepatocellular carcinoma (HCC)	Asian	Case-control study				IL17F	rs763780 T/C	T/C
25429834	Xi, X. E	2015	Hepatocellular carcinoma (HCC)	Asian	Case-control study				IL17A	rs4711998 A/G	A/G
25431761	Escamilla Tilch, M	2014	Leprosy	Mixed	Case-control study	68	69	137	IL17A	rs2275913 G/A	G/A
25431761	Escamilla Tilch, M	2014	Leprosy	Mixed	Case-control study				IL17F	rs763780 T/C	T/C
25469655	Ma, Q. Y	2015	Non-small cell lung cancer (NSCLC)	Asian	Case-control study	330	382	712	IL17A	rs142078089 A/G	A/G
25469655	Ma, Q. Y	2015	Non-small cell lung cancer (NSCLC)	Asian	Case-control study	330	382	712	IL17A	rs17879568 G/A	G/A
25484349	Lee, Y. C	2015	Papillary thyroid cancer (PTC)	Asian	Case-control study	94	260	354	IL17A	rs3819024 A/G	A/G
25484349	Lee, Y. C	2015	Papillary thyroid cancer (PTC)	Asian	Case-control study				IL17A	rs2275913 G/A	G/A
25484349	Lee, Y. C	2015	Papillary thyroid cancer (PTC)	Asian	Case-control study				IL17RA	rs879575 C/T	C/T
25484349	Lee, Y. C	2015	Papillary thyroid cancer (PTC)	Asian	Case-control study				IL17RA	rs879577 C/T	C/T

25484349	Lee, Y. C	2015	Papillary thyroid cancer (PTC)	Asian	Case-control study				IL17RA	rs2229151 G/A	G/A
25484349	Lee, Y. C	2015	Papillary thyroid cancer (PTC)	Asian	Case-control study				IL17RA	rs4819554 G/A	G/A
25484349	Lee, Y. C	2015	Papillary thyroid cancer (PTC)	Asian	Case-control study				IL17RB	rs2232350 T/C	T/C
25484349	Lee, Y. C	2015	Papillary thyroid cancer (PTC)	Asian	Case-control study				IL17RB	rs1025689 C/G	C/G
25510225	Boroń, D	2014	Osteoporosis	Caucasian	Case-control study	306	158	464	IL17A	rs2275913 G/A	G/A
25579009	Li, N	2015	Oral squamous cell carcinoma (OSCC)	Asian	Case-control study	121	103	224	IL17A	rs2275913 G/A	G/A
25579009	Li, N	2015	Oral squamous cell carcinoma (OSCC)	Asian	Case-control study				IL17F	rs763780 T/C	T/C
25579009	Li, N	2015	Oral squamous cell carcinoma (OSCC)	Asian	Case-control study				IL17F	rs9382084 G/T	G/T
25596084	Lv, Q	2015	Cervical cancer	Asian	Case-control study	264	264	528	IL17A	rs2275913 G/A	G/A
25596084	Lv, Q	2015	Cervical cancer	Asian	Case-control study				IL17A	rs3748067 C/T	C/T
25596084	Lv, Q	2015	Cervical cancer	Asian	Case-control study				IL17A	rs3819025 G/A	G/A
25596084	Lv, Q	2015	Cervical cancer	Asian	Case-control study				IL17F	rs763780 T/C	T/C
25596084	Lv, Q	2015	Cervical cancer	Asian	Case-control study				IL17F	rs9382084 G/T	G/T
25596084	Lv, Q	2015	Cervical cancer	Asian	Case-control study				IL17F	rs1266828 G/A	G/A
25615631	Vargas Alarcón, G	2015	Coronary artery disease (CAD)	Mixed	Case-control study	900	667	1567	IL17A	rs8193036 C/T	C/T
25615631	Vargas Alarcón, G	2015	Coronary artery disease (CAD)	Mixed	Case-control study				IL17A	rs3819024 A/G	A/G
25615631	Vargas Alarcón, G	2015	Coronary artery disease (CAD)	Mixed	Case-control study				IL17A	rs2275913 G/A	G/A
25615631	Vargas Alarcón, G	2015	Coronary artery disease (CAD)	Mixed	Case-control study				IL17A	rs8193037 G/A	G/A
25636567	Coto, E	2015	Chronic kidney disease (CKD)	Caucasian	Case-control study	90	560	650	IL17RA	rs4819554 G/A	G/A
25663919	Gao, Y. W	2015	Gastric Cancer (GC)	Asian	Case-control study	572	572	1144	IL17A	rs2275913 G/A	G/A
25663919	Gao, Y. W	2015	Gastric Cancer (GC)	Asian	Case-control study				IL17F	rs763780 T/C	T/C
25663919	Gao, Y. W	2015	Gastric Cancer (GC)	Asian	Case-control study				IL17A	rs3748067 C/T	C/T
25680555	Nemati, K	2015	Colorectal cance (CRC)	Caucasian	Case-control study	202	203	405	IL17A	rs2275913 G/A	G/A
25680555	Nemati, K	2015	Colorectal cance (CRC)	Caucasian	Case-control study				IL17F	rs763780 T/C	T/C
25857634	Bulat Kardum, L. J	2015	Tuberculosis (TB)	Caucasian	Case-control study	244	407	651	IL17A	rs2275913 G/A	G/A

25857634	Bulat Kardum, L. J	2015	Tuberculosis (TB)	Caucasian	Case-control study	222	429	651	IL17F	rs763780 T/C	T/C
25871515	Shen, L	2015	Rheumatoid arthritis (RA)	Asian	Case-control study	615	839	1454	IL17A	rs2275913 G/A	G/A
25871515	Shen, L	2015	Rheumatoid arthritis (RA)	Asian	Case-control study				IL17A	rs3819024 A/G	A/G
25871515	Shen, L	2015	Rheumatoid arthritis (RA)	Asian	Case-control study				IL17A	rs3819025 G/A	G/A
25871515	Shen, L	2015	Rheumatoid arthritis (RA)	Asian	Case-control study				IL17A	rs4711998 A/G	A/G
25871515	Shen, L	2015	Rheumatoid arthritis (RA)	Asian	Case-control study				IL17A	rs8193036 C/T	C/T
25871515	Shen, L	2015	Rheumatoid arthritis (RA)	Asian	Case-control study				IL17A	rs8193037 G/A	G/A
26017027	Aytekin, N	2015	Aopecia areata	Caucasian	Case-control study	100	71	171	IL17F	rs763780 T/C	T/C
26045845	Du, J	2015	Tuberculosis (TB)	Asian	Case-control study	428	428	856	IL17A	rs2275913 G/A	G/A
26045845	Du, J	2015	Tuberculosis (TB)	Asian	Case-control study				IL17A	rs3748067 C/T	C/T
26045845	Du, J	2015	Tuberculosis (TB)	Asian	Case-control study				IL17F	rs763780 T/C	T/C
26045845	Du, J	2015	Tuberculosis (TB)	Asian	Case-control study				IL17F	rs9382084 G/T	G/T
26073462	He, Y	2017	Non-small cell lung cancer (NSCLC)	Asian	Case-control study	173	358	531	IL17A	rs2275913 G/A	G/A
26073462	He, Y	2017	Non-small cell lung cancer (NSCLC)	Asian	Case-control study	95	358	453	IL17A	rs2275913 G/A	G/A
26073462	He, Y	2017	Non-small cell lung cancer (NSCLC)	Asian	Case-control study	23	358	381	IL17A	rs2275913 G/A	G/A
26073462	He, Y	2017	Small cell lung cancer (SCLC)	Asian	Case-control study	29	358	387	IL17A	rs2275913 G/A	G/A
26073462	He, Y	2017	Non-small cell lung cancer (NSCLC)	Asian	Case-control study				IL17F	rs12203582 G/A	G/A
26073462	He, Y	2017	Non-small cell lung cancer (NSCLC)	Asian	Case-control study				IL17F	rs12203582 G/A	G/A
26073462	He, Y	2017	Non-small cell lung cancer (NSCLC)	Asian	Case-control study				IL17F	rs12203582 G/A	G/A
26073462	He, Y	2017	Lung cancer (LC)	Asian	Case-control study				IL17F	rs12203582 G/A	G/A
26073462	He, Y	2017	Lung cancer (LC)	Asian	Case-control study	320	358	678	IL17A	rs3748067 C/T	C/T
26073462	He, Y	2017	Lung cancer (LC)	Asian	Case-control study				IL17A	rs3819025 G/A	G/A
26073462	He, Y	2017	Lung cancer (LC)	Asian	Case-control study				IL17F	rs763780 T/C	T/C
26073462	He, Y	2017	Lung cancer (LC)	Asian	Case-control study				IL17F	rs1266828 G/A	G/A
26108303	Raeiszadeh J, S	2015	Asthma	Other	Case-control study	419	393	812	IL17F	rs1889570 C/T	C/T
26119194	Anvari, F	2015	Pre-eclampsia (PE)	Caucasian	Case-control study	261	278	539	IL17A	rs2275913 G/A	G/A

26119194	Anvari, F	2015	Pre-eclampsia (PE)	Caucasian	Case-control study				IL17F	rs763780 T/C	T/C
26223249	Han, R	2015	Coal workers' pneumoconiosis (CWP)	Asian	Case-control study	692	687	1379	IL17A	rs2275913 G/A	G/A
26223249	Han, R	2015	Coal workers' pneumoconiosis (CWP)	Asian	Case-control study				IL17A	rs3748067 C/T	C/T
26223249	Han, R	2015	Coal workers' pneumoconiosis (CWP)	Asian	Case-control study				IL17A	rs4711998 A/G	A/G
26232893	Carvalho, C	2016	Rheumatoid arthritis (RA)	Mixed	Case-control study	100	75	175	IL17A	rs2275913 G/A	G/A
26232893	Carvalho, C	2016	Rheumatoid arthritis (RA)	Mixed	Case-control study	31	75	106	IL17A	rs2275913 G/A	G/A
26232893	Carvalho, C	2016	Rheumatoid arthritis (RA)	Mixed	Case-control study				IL17F	rs763780 T/C	T/C
26232893	Carvalho, C	2016	Rheumatoid arthritis (RA)	Mixed	Case-control study				IL17F	rs763780 T/C	T/C
26261639	Hou, C	2015	Gastric Cancer (GC)	Asian	Case-control study	326	326	652	IL17A	rs2275913 G/A	G/A
26261639	Hou, C	2015	Gastric Cancer (GC)	Asian	Case-control study				IL17A	rs3748067 C/T	C/T
26261639	Hou, C	2015	Gastric Cancer (GC)	Asian	Case-control study				IL17F	rs763780 T/C	T/C
26309658	Ge, J	2015	Hepatitis B Virus (HBV)	Asian	Case-control study	163	168	331	IL17A	rs2275913 G/A	G/A
26309658	Ge, J	2015	Hepatitis B Virus (HBV)	Asian	Case-control study				IL17F	rs763780 T/C	T/C
26337284	Yue, Y. X	2016	Myasthenia gravis (MG)	Asian	Case-control study	480	487	967	IL17A	rs8193036 C/T	C/T
26337284	Yue, Y. X	2016	Myasthenia gravis (MG)	Asian	Case-control study				IL17A	rs2275913 G/A	G/A
26337284	Yue, Y. X	2016	Myasthenia gravis (MG)	Asian	Case-control study				IL17A	rs3748067 C/T	C/T
26337284	Yue, Y. X	2016	Myasthenia gravis (MG)	Asian	Case-control study				IL17F	rs763780 T/C	T/C
26339129	Zacarias, J. M	2015	Periodontitis	Mixed	Case-control study	140	173	313	IL17A	rs2275913 G/A	G/A
26339129	Zacarias, J. M	2015	Periodontitis	Mixed	Case-control study				IL17F	rs763780 T/C	T/C
26345782	Shi, G. C	2015	Tuberculosis (TB)	Asian	Case-control study	336	351	687	IL17A	rs2275913 G/A	G/A
26345782	Shi, G. C	2015	Tuberculosis (TB)	Asian	Case-control study				IL17A	rs3748067 C/T	C/T
26345782	Shi, G. C	2015	Tuberculosis (TB)	Asian	Case-control study				IL17F	rs763780 T/C	T/C
26347322	Batalla, A	2015	Psoriasis	Caucasian	Case-control study	580	567	1147	IL17RA	rs4819554 G/A	G/A
26347322	Batalla, A	2015	Psoriasis	Caucasian	Case-control study				IL17F	rs2397084 T/C	T/C
26347322	Batalla, A	2015	Psoriasis	Caucasian	Case-control study				IL17E	rs79877597 C/A	C/A
26367076	E. LBassuoni MA	2015	Chronic infections with HCV	African	Case-control study	75	20	95	IL17A	rs2275913 G/A	G/A
26367076	E. LBassuoni MA	2015	Hepatocellular carcinoma (HCC)	African	Case-control study	35	20	55	IL17A	rs2275913 G/A	G/A
26400516	Romanowski, M	2015	Post-transplant diabetes	Caucasian	Case-control study	23	146	169	IL17A	rs2275913 G/A	G/A

			mellitus (PTDM)								
26400516	Romanowski, M	2015	Post-transplant diabetes mellitus (PTDM)	Caucasian	Case-control study				IL17F	rs11465553 C/T	C/T
26400516	Romanowski, M	2015	Post-transplant diabetes mellitus (PTDM)	Caucasian	Case-control study				IL17F	rs2397084 T/C	T/C
26400516	Romanowski, M	2015	Post-transplant diabetes mellitus (PTDM)	Caucasian	Case-control study				IL17F	rs763780 T/C	T/C
26415694	Prieto Pérez, R	2015	Psoriasis	Caucasian	Case-control study	194	197	391	IL17F	rs763780 T/C	T/C
26415694	Prieto Pérez, R	2015	Psoriasis	Caucasian	Case-control study				IL17A	rs2275913 G/A	G/A
26415694	Prieto Pérez, R	2015	Psoriasis	Caucasian	Case-control study				IL17A	rs10484879 C/A	C/A
26436389	Geng, G. Y	2015	Coronary artery disease (CAD)	Asian	Case-control study	306	306	612	IL17F	rs763780 T/C	T/C
26436389	Geng, G. Y	2015	Coronary artery disease (CAD)	Asian	Case-control study				IL17A	rs2275913 G/A	G/A
26451724	Wang, H	2015	Pre-eclampsia (PE)	Asian	Case-control study	1031	1298	2329	IL17F	rs763780 T/C	T/C
26451724	Wang, H	2015	Pre-eclampsia (PE)	Asian	Case-control study				IL17A	rs2275913 G/A	G/A
26451724	Wang, H	2015	Pre-eclampsia (PE)	Asian	Case-control study				IL17RA	rs4819554 G/A	G/A
26464720	Cong, J	2015	Cervical cancer	Asian	Case-control study	352	352	704	IL17A	rs2275913 G/A	G/A
26464720	Cong, J	2015	Cervical cancer	Asian	Case-control study				IL17A	rs3748067 C/T	C/T
26464720	Cong, J	2015	Cervical cancer	Asian	Case-control study				IL17F	rs763780 T/C	T/C
26367076	E. LBassuoni MA	2015	Chronic infections with HCV	African	Case-control study	75	20	95	IL17A	rs2275913 G/A	G/A
26367076	E. LBassuoni MA	2015	Hepatocellular carcinoma (HCC)	African	Case-control study	35	20	55	IL17A	rs2275913 G/A	G/A
26400516	Romanowski, M	2015	Post-transplant diabetes mellitus (PTDM)	Caucasian	Case-control study	23	146	169	IL17A	rs2275913 G/A	G/A
26400516	Romanowski, M	2015	Post-transplant diabetes mellitus (PTDM)	Caucasian	Case-control study				IL17F	rs11465553 C/T	C/T
26467330	Zidan, H. E	2015	Recurrent Miscarriage (RM)	African	Case-control study	120	120	240	IL17A	rs2275913 G/A	G/A
26467330	Zidan, H. E	2015	Recurrent Miscarriage (RM)	African	Case-control study				IL17F	rs763780 T/C	T/C
26505366	Li, L	2015	Cervical cancer	Asian	Case-control study	216	432	648	IL17A	rs2275913 G/A	G/A
26505366	Li, L	2015	Cervical cancer	Asian	Case-control study				IL17A	rs3748067 C/T	C/T
26505366	Li, L	2015	Cervical cancer	Asian	Case-control study				IL17A	rs3819025 G/A	G/A

26505366	Li, L	2015	Cervical cancer	Asian	Case-control study				IL17F	rs763780 T/C	T/C
26505366	Li, L	2015	Cervical cancer	Asian	Case-control study				IL17F	rs9382084 G/T	G/T
26505366	Li, L	2015	Cervical cancer	Asian	Case-control study				IL17F	rs1266828 G/A	G/A
26515887	Hammad, A	2016	Systemic lupus erythematosus (SLE)	African	Case-control study	115	295	410	IL17A	rs2275913 G/A	G/A
26515887	Hammad, A	2016	Systemic lupus erythematosus (SLE)	African	Case-control study				IL17F	rs763780 T/C	T/C
26515887	Hammad, A	2016	Systemic lupus erythematosus (SLE)	African	Case-control study				IL17F	rs2397084 T/C	T/C
26535650	Qi, W. T	2015	Gastric Cancer (GC)	Asian	Case-control study	252	252	504	IL17A	rs2275913 G/A	G/A
26535650	Qi, W. T	2015	Gastric Cancer (GC)	Asian	Case-control study				IL17A	rs3748067 C/T	C/T
26535650	Qi, W. T	2015	Gastric Cancer (GC)	Asian	Case-control study				IL17F	rs763780 T/C	T/C
26535675	Sun, L. X	2015	Cervical cancer	Asian	Case-control study	306	354	660	IL17A	rs2275913 G/A	G/A
26535675	Sun, L. X	2015	Cervical cancer	Asian	Case-control study				IL17F	rs763780 T/C	T/C
26546176	Wang, J	2016	Hepatitis B Virus (HBV)	Asian	Case-control study	130	171	301	IL17A	rs4711998 A/G	A/G
26546176	Wang, J	2016	Hepatitis B Virus (HBV)	Asian	Case-control study				IL17A	rs2275913 G/A	G/A
26546176	Wang, J	2016	Hepatitis B Virus (HBV)	Asian	Case-control study				IL17F	rs763780 T/C	T/C
26546176	Wang, J	2016	Hepatitis B Virus (HBV)	Asian	Case-control study	132	171	303	IL17A	rs4711998 A/G	A/G
26546176	Wang, J	2016	Hepatitis B Virus (HBV)	Asian	Case-control study				IL17A	rs2275913 G/A	G/A
26546176	Wang, J	2016	Hepatitis B Virus (HBV)	Asian	Case-control study				IL17F	rs763780 T/C	T/C
26546176	Wang, J	2016	Hepatitis B Virus (HBV)	Asian	Case-control study	132	130	262	IL17A	rs4711998 A/G	A/G
26546176	Wang, J	2016	Hepatitis B Virus (HBV)	Asian	Case-control study				IL17A	rs2275913 G/A	G/A
26546176	Wang, J	2016	Hepatitis B Virus (HBV)	Asian	Case-control study				IL17F	rs763780 T/C	T/C
26617905	Shuang, L	2015	Coronary artery disease (CAD)	Asian	Case-control study	415	448	863	IL17A	rs2275913 G/A	G/A
26617905	Shuang, L	2015	Coronary artery disease (CAD)	Asian	Case-control study				IL17A	rs3819025 G/A	G/A
26617905	Shuang, L	2015	Coronary artery disease (CAD)	Asian	Case-control study				IL17A	rs3748067 C/T	C/T
26620416	Li, H	2017	Immune thrombocytopenia (ITP)	Asian	Case-control study	165	149	314	IL17F	rs763780 T/C	T/C
26840977	Milano, M	2016	Tuberculosis (TB)	Mixed	Case-control study	191	175	366	IL17A	rs2275913 G/A	G/A

26890073	Zhou, Z	2016	Gout	Asian	Case-control study	1101	1239	2340	IL17A	rs2275913 G/A	G/A
26890073	Zhou, Z	2016	Gout	Asian	Case-control study				IL17F	rs763780 T/C	T/C
26890073	Zhou, Z	2016	Gout	Asian	Case-control study				IL17RA	rs4819554 G/A	G/A
26909948	Zheng, X. S	2016	Coronary artery disease (CAD)	Asian	Case-control study	372	372	744	IL17A	rs2275913 G/A	G/A
26909948	Zheng, X. S	2016	Coronary artery disease (CAD)	Asian	Case-control study				IL17A	rs3748067 C/T	C/T
26910014	Chaudhari, H. L	2016	Periodontitis	Other	Case-control study	35	35	70	IL17A	rs2275913 G/A	G/A
26910014	Chaudhari, H. L	2016	Periodontitis	Other	Case-control study				IL17A	rs2275913 G/A	G/A
26924897	Borilova L, P	2016	Periodontitis	Caucasian	Case-control study	244	154	398	IL17A	rs2275913 G/A	G/A
26924897	Borilova L, P	2016	Type 1 diabetes mellitus (T1DM)	Caucasian	Case-control study				IL17A	rs2275913 G/A	G/A
26954344	Du, J	2016	Asthma	Asian	Case-control study	125	132	257	IL17A	rs2275913 G/A	G/A
26954344	Du, J	2016	Asthma	Asian	Case-control study				IL17A	rs8193036 C/T	C/T
26954344	Du, J	2016	Asthma	Asian	Case-control study				IL17F	rs763780 T/C	T/C
27003425	Korytina, G. F	2016	Chronic obstructive pulmonary disease (COPD)	Tatars	Case-control study	425	457	882	IL17A	rs4711998 A/G	A/G
27003425	Korytina, G. F	2016	Chronic obstructive pulmonary disease (COPD)	Tatars	Case-control study				IL17A	rs1974226 C/T	C/T
27007229	Mokhtar, G. M	2016	Immune thrombocytopenia (ITP)	African	Case-control study	50	50	100	IL17F	rs763780 T/C	T/C
27021337	Xu, H	2016	Henoch–Schonlein purpura (HSP).	Asian	Case-control study	148	202	350	IL17A	rs2275913 G/A	G/A
27021337	Xu, H	2016	Henoch–Schonlein purpura (HSP).	Asian	Case-control study				IL17A	rs8193037 G/A	G/A
27021337	Xu, H	2016	Henoch–Schonlein purpura (HSP).	Asian	Case-control study				IL17A	rs3819025 G/A	G/A
27021337	Xu, H	2016	Henoch–Schonlein purpura (HSP).	Asian	Case-control study				IL17F	rs763780 T/C	T/C
27021337	Xu, H	2016	Henoch–Schonlein purpura (HSP).	Asian	Case-control study				IL17F	rs9463772 C/T	C/T
27097946	Yang, L. J	2016	Gastric Cancer (GC)	Asian	Case-control study	386	374	760	IL17A	rs2275913 G/A	G/A

27097946	Yang, L. J	2016	Gastric Cancer (GC)	Asian	Case-control study				IL17A	rs3748067 C/T	C/T
27097946	Yang, L. J	2016	Gastric Cancer (GC)	Asian	Case-control study				IL17A	rs4711998 A/G	A/G
27097946	Yang, L. J	2016	Gastric Cancer (GC)	Asian	Case-control study				IL17F	rs763780 T/C	T/C
27155288	Rogo, L. D	2016	Influenza A/H3N2 virus infection	Caucasian	Case-control study	96	147	243	IL17A	rs2275913 G/A	G/A
27155288	Rogo, L. D	2016	Influenza like illness (ILI)	Caucasian	Case-control study	114	147	261	IL17A	rs2275913 G/A	G/A
27155445	Erkol İnal, E	2016	Ankylosing spondylitis (AS)	Caucasian	Case-control study	101	106	207	IL17F	rs763780 T/C	T/C
27155445	Erkol İnal, E	2016	Ankylosing spondylitis (AS)	Caucasian	Case-control study				IL17F	rs2397084 T/C	T/C
27155445	Erkol İnal, E	2016	Ankylosing spondylitis (AS)	Caucasian	Case-control study	101	106	207	IL17A	rs2275913 G/A	G/A
27169372	Pawlik, A	2016	Rheumatoid arthritis (RA)	Caucasian	Case-control study	422	337	759	IL17A	rs2275913 G/A	G/A
27169372	Pawlik, A	2016	Rheumatoid arthritis (RA)	Caucasian	Case-control study				IL17F	rs763780 T/C	T/C
27169372	Pawlik, A	2016	Rheumatoid arthritis (RA)	Caucasian	Case-control study				IL17F	rs11465553 C/T	C/T
27169372	Pawlik, A	2016	Rheumatoid arthritis (RA)	Caucasian	Case-control study				IL17F	rs2397084 T/C	T/C
27312555	Liu, S	2016	Immune thrombocytopenia (ITP)	Asian	Case-control study	146	137	283	IL17A	rs2275913 G/A	G/A
27312555	Liu, S	2016	Immune thrombocytopenia (ITP)	Asian	Case-control study				IL17F	rs763780 T/C	T/C
27320770	Paradowska G, A	2016	Systemic lupus erythematosus (SLE)	Caucasian	Case-control study	134	106	240	IL17F	rs763780 T/C	T/C
27320770	Paradowska G, A	2016	Systemic lupus erythematosus (SLE)	Caucasian	Case-control study				IL17F	rs2397084 T/C	T/C
27323118	Xu, B. L	2016	Gastric Cancer (GC)	Asian	Case-control study	202	237	439	IL17A	rs2275913 G/A	G/A
27339100	Wang, M	2016	Tuberculosis (TB)	Asian	Case-control study	1601	1526	3127	IL17A	rs2275913 G/A	G/A
27339100	Wang, M	2016	Tuberculosis (TB)	Asian	Case-control study				IL17A	rs3819024 A/G	A/G
27339100	Wang, M	2016	Tuberculosis (TB)	Asian	Case-control study				IL17A	rs8193036 C/T	C/T
27339100	Wang, M	2016	Tuberculosis (TB)	Asian	Case-control study				IL17A	rs3748067 C/T	C/T
27339100	Wang, M	2016	Tuberculosis (TB)	Asian	Case-control study				IL17F	rs763780 T/C	T/C
27415816	Vidal C, J. R	2016	Ankylosing spondylitis (AS)	Caucasian	Case-control study	180	300	480	IL17RA	rs4819554 G/A	G/A
27415816	Vidal C, J. R	2016	Ankylosing spondylitis (AS)	Caucasian	Case-control study	419	656	1075	IL17RA	rs4819554 G/A	G/A
27525907	Zhao, W. M	2016	Gastric Cancer (GC)	Asian	Case-control study	153	207	360	IL17A	rs2275913 G/A	G/A
27525907	Zhao, W. M	2016	Gastric Cancer (GC)	Asian	Case-control study				IL17F	rs763780 T/C	T/C
27525938	Su, G. B	2016	Coronary artery disease	Asian	Case-control study	219	219	438	IL17A	rs3819024 A/G	A/G

			(CAD)								
27525938	Su, G. B	2016	Coronary artery disease (CAD)	Asian	Case-control study				IL17A	rs2275913 G/A	G/A
27525938	Su, G. B	2016	Coronary artery disease (CAD)	Asian	Case-control study				IL17A	rs8193037 G/A	G/A
27525938	Su, G. B	2016	Coronary artery disease (CAD)	Asian	Case-control study				IL17A	rs8193036 C/T	C/T
27525938	Su, G. B	2016	Coronary artery disease (CAD)	Asian	Case-control study				IL17A	rs3748067 C/T	C/T
27561723	Resende, E. P	2017	Asthma	Caucasian	Case-control study	83	192	275	IL17A	rs2275913 G/A	G/A
27561723	Resende, E. P	2017	Rhinitis	Caucasian	Case-control study	149	192	341	IL17A	rs2275913 G/A	G/A
27577072	Zhou, F	2016	Gastric Cancer (GC)	Asian	Case-control study	1121	1216	2337	IL17A	rs1974226 C/T	C/T
27577072	Zhou, F	2016	Gastric Cancer (GC)	Asian	Case-control study				IL17A	rs2275913 G/A	G/A
27577072	Zhou, F	2016	Gastric Cancer (GC)	Asian	Case-control study				IL17A	rs3819024 A/G	A/G
27577072	Zhou, F	2016	Gastric Cancer (GC)	Asian	Case-control study				IL17A	rs4711998 A/G	A/G
27577072	Zhou, F	2016	Gastric Cancer (GC)	Asian	Case-control study				IL17A	rs8193036 C/T	C/T
27591988	Białecka, M	2016	Psoriasis	Caucasian	Case-control study	407	205	612	IL17A	rs2275913 G/A	G/A
27591988	Białecka, M	2016	Psoriasis	Caucasian	Case-control study				IL17F	rs763780 T/C	T/C
27591988	Białecka, M	2016	Psoriasis	Caucasian	Case-control study				IL17F	rs11465553 C/T	C/T
27591988	Białecka, M	2016	Psoriasis	Caucasian	Case-control study				IL17F	rs2397084 T/C	T/C
27595159	Kharwar, N. K	2017	Guillain-Barré syndrome (GBS)	Other	Case-control study	80	75	155	IL17F	rs2397084 T/C	T/C
27595159	Kharwar, N. K	2017	Guillain-Barré syndrome (GBS)	Other	Case-control study				IL17F	rs763780 T/C	T/C
27774581	Kim, S. Y	2018	Psoriasis	Asian	Case-control study	208	266	474	IL17F	rs763780 T/C	T/C
28098168	Rolandelli, A	2017	Tuberculosis (TB)	Mixed	Case-control study	185	207	392	IL17A	rs2275913 G/A	G/A
28143790	Marwa, O. S	2017	Rheumatoid arthritis (RA)	Caucasian	Case-control study	108	202	310	IL17A	rs2275913 G/A	G/A
28143790	Marwa, O. S	2017	Rheumatoid arthritis (RA)	Caucasian	Case-control study				IL17F	rs763780 T/C	T/C
28143790	Marwa, O. S	2017	Rheumatoid arthritis (RA)	Caucasian	Case-control study				IL17F	rs2397084 T/C	T/C
28224332	Tian, J	2017	Necrotizing enterocolitis (NEC)	Asian	Case-control study	102	120	222	IL17A	rs2275913 G/A	G/A
28224332	Tian, J	2017	Necrotizing enterocolitis	Asian	Case-control study				IL17F	rs763780 T/C	T/C

			(NEC)								
28277785	Ren, W	2017	Hepatitis B Virus (HBV)	Asian	Case-control study	478	577	1055	IL17A	rs2275913 G/A	G/A
28277785	Ren, W	2017	Hepatitis B Virus (HBV)	Asian	Case-control study	550	589	1139	IL17F	rs763780 T/C	T/C
28362993	Si, F. Z	2017	Laryngeal cancer	Asian	Case-control study	325	325	650	IL17A	rs2275913 G/A	G/A
28362993	Si, F. Z	2017	Laryngeal cancer	Asian	Case-control study				IL17A	rs3748067 C/T	C/T
28362993	Si, F. Z	2017	Laryngeal cancer	Asian	Case-control study				IL17F	rs763780 T/C	T/C
28410448	Neco, Hvpc	2017	HTLV-1-associated myelopathy/tropical spastic paraparesis (HAM/TSP)	Mixed	Case-control study	29	87	116	IL17A	rs2275913 G/A	G/A
28470012	Reis, P. G	2017	Chagas disease (CD)	Mixed	Case-control study	212	150	362	IL17A	rs2275913 G/A	G/A
28470012	Reis, P. G	2017	Chagas disease (CD)	Mixed	Case-control study				IL17F	rs763780 T/C	T/C
28470012	Reis, P. G	2017	Chagas disease (CD)	Mixed	Case-control study	48	150	198	IL17A	rs2275913 G/A	G/A
28470012	Reis, P. G	2017	Chagas disease (CD)	Mixed	Case-control study				IL17F	rs763780 T/C	T/C
28481151	Hassani, E	2017	Silicotic	Kurdish	Case-control study	48	62	110	IL17A	rs4711998 A/G	A/G
28481151	Hassani, E	2017	Silicotic	Kurdish	Case-control study				IL17F	rs763780 T/C	T/C
28547498	Gomes da Silva, I. I. F	2017	Rheumatoid arthritis (RA)	Mixed	Case-control study	127	134	261	IL17A	rs2275913 G/A	G/A
28547498	Gomes da Silva, I. I. F	2017	Rheumatoid arthritis (RA)	Mixed	Case-control study				IL17F	rs763780 T/C	T/C
28595093	Ju, H	2017	Hepatitis B Virus (HBV)	Asian	Case-control study	139	145	284	IL17A	rs4711998 A/G	A/G
28595093	Ju, H	2017	Hepatitis B Virus (HBV)	Asian	Case-control study				IL17A	rs2275913 G/A	G/A
28595093	Ju, H	2017	Hepatitis B Virus (HBV)	Asian	Case-control study				IL17F	rs763780 T/C	T/C
28647382	Holster, A	2018	Bronchiolitis	Caucasian	Case-control study	165	405	570	IL17A	rs2275913 G/A	G/A
28731539	Liehu M, M	2017	Bacillus Calmette-Guérin (BCG) osteitis	Caucasian	Case-control study	132	405	537	IL17A	rs2275913 G/A	G/A
28741807	Gueiros, L. A	2018	Oral lichen planus (OLP)	Mixed	Case-control study	83	99	182	IL17A	rs2275913 G/A	G/A
28786198	Kasamatsu, T	2018	Multiple myeloma (MM)	Asian	Case-control study	120	201	321	IL17A	rs2275913 G/A	G/A
28873198	Niu, A. Q	2017	Cervical cancer	Asian	Case-control study	185	370	555	IL17A	rs2275913 G/A	G/A
28873198	Niu, A. Q	2017	Cervical cancer	Asian	Case-control study				IL17A	rs3748067 C/T	C/T
28965252	Singh, R	2018	Functional dyspepsia (FD)	Other	Case-control study	26	195	221	IL17F	rs2397084 T/C	T/C
28965252	Singh, R	2018	Functional dyspepsia (FD)	Other	Case-control study	55	250	305	IL17F	rs2397084 T/C	T/C

28965252	Singh, R	2018	Functional dyspepsia (FD)	Other	Case-control study	156	250	406	IL17F	rs2397084 T/C	T/C
28965252	Singh, R	2018	Functional dyspepsia (FD)	Other	Case-control study				IL17F	rs763780 T/C	T/C
28965252	Singh, R	2018	Functional dyspepsia (FD)	Other	Case-control study				IL17F	rs763780 T/C	T/C
28965252	Singh, R	2018	Functional dyspepsia (FD)	Other	Case-control study				IL17F	rs763780 T/C	T/C
29194748	Vrgoc, G	2018	Osteoarthritis (OA)	Caucasian	Case-control study	186	407	593	IL17A	rs2275913 G/A	G/A
29194748	Vrgoc, G	2018	Osteoarthritis (OA)	Caucasian	Case-control study	172	407	579	IL17A	rs2275913 G/A	G/A
29194748	Vrgoc, G	2018	Osteoarthritis (OA)	Caucasian	Case-control study	210	528	738	IL17F	rs763780 T/C	T/C
29194748	Vrgoc, G	2018	Osteoarthritis (OA)	Caucasian	Case-control study	232	528	760	IL17F	rs763780 T/C	T/C
29194748	Vrgoc, G	2018	Osteoarthritis (OA)	Caucasian	Case-control study	230	186	416	IL17F	rs1889570 C/T	C/T
29194748	Vrgoc, G	2018	Osteoarthritis (OA)	Caucasian	Case-control study	244	186	430	IL17F	rs1889570 C/T	C/T
29372577	Mansouri, F	2018	Tuberculosis (TB)	Caucasian	Case-control study	76	119	195	IL17A	rs2275913 G/A	G/A
29400002	Vahabi, S	2018	Periodontitis	Caucasian	Case-control study	99	75	174	IL17F	rs2397084 T/C	T/C
29530464	Tang, H	2018	Viral myocarditis (VMC)	Asian	Case-control study	236	259	495	IL17A	rs2275913 G/A	G/A
29530464	Tang, H	2018	Viral myocarditis (VMC)	Asian	Case-control study				IL17A	rs3819025 G/A	G/A
29530464	Tang, H	2018	Viral myocarditis (VMC)	Asian	Case-control study				IL17A	rs3748067 C/T	C/T
29549443	Li, F	2018	Enterovirus 71 infection	Asian	Case-control study	221	330	551	IL17F	rs1889570 C/T	C/T
29549443	Li, F	2018	Enterovirus 71 infection	Asian	Case-control study	115	330	445	IL17F	rs1889570 C/T	C/T
29549443	Li, F	2018	Enterovirus 71 infection	Asian	Case-control study				IL17F	rs4715290 A/G	A/G
29549443	Li, F	2018	Enterovirus 71 infection	Asian	Case-control study				IL17F	rs4715290 A/G	A/G
29584788	Dhaouadi, T	2018	Rheumatoid arthritis (RA)	Caucasian	Case-control study	115	91	206	IL17A	rs2275913 G/A	G/A
29584788	Dhaouadi, T	2018	Rheumatoid arthritis (RA)	Caucasian	Case-control study				IL17RC	rs708567 C/T	C/T
29689450	Bedoui, S. A	2018	Colorectal cance (CRC)	Caucasian	Case-control study	294	268	562	IL17A	rs3819024 A/G	A/G
29689450	Bedoui, S. A	2018	Colorectal cance (CRC)	Caucasian	Case-control study				IL17A	rs2275913 G/A	G/A
29689450	Bedoui, S. A	2018	Colorectal cance (CRC)	Caucasian	Case-control study				IL17A	rs3819025 G/A	G/A
29689450	Bedoui, S. A	2018	Colorectal cance (CRC)	Caucasian	Case-control study				IL17A	rs10484879 C/A	C/A
29689450	Bedoui, S. A	2018	Colorectal cance (CRC)	Caucasian	Case-control study				IL17A	rs7747909 G/A	G/A
29689450	Bedoui, S. A	2018	Colorectal cance (CRC)	Caucasian	Case-control study				IL17A	rs3748067 C/T	C/T
29704988	Dai, Y	2018	Chronic obstructive pulmonary disease (COPD)	Asian	Case-control study	152	201	353	IL17A	rs2275913 G/A	G/A
29704988	Dai, Y	2018	Chronic obstructive pulmonary disease (COPD)	Asian	Case-control study				IL17A	rs3819024 A/G	A/G

29704988	Dai, Y	2018	Chronic obstructive pulmonary disease (COPD)	Asian	Case-control study				IL17A	rs8193036 C/T	C/T
29704988	Dai, Y	2018	Chronic obstructive pulmonary disease (COPD)	Asian	Case-control study				IL17A	rs3748067 C/T	C/T
29704988	Dai, Y	2018	Chronic obstructive pulmonary disease (COPD)	Asian	Case-control study				IL17F	rs763780 T/C	T/C
29733805	Akbulut, U. E	2019	Non-alcoholic fatty liver disease (NAFLD)	Caucasian	Case-control study	101	85	186	IL17A	rs2275913 G/A	G/A
29764467	Wang, P	2018	Ossification of the posterior longitudinal ligament (OPLL)	Asian	Case-control study	100	100	200	IL17RC	rs199772854 C/A	C/A
29764467	Wang, P	2018	Ossification of the posterior longitudinal ligament (OPLL)	Asian	Case-control study				IL17RC	rs76999397 G/A	G/A
29764467	Wang, P	2018	Ossification of the posterior longitudinal ligament (OPLL)	Asian	Case-control study				IL17RC	rs189013166 G/A	G/A
29849482	Rocha Loures, M. A	2018	Spondyloarthritis (SpA)	Mixed	Case-control study	172	210	382	IL17A	rs2275913 G/A	G/A
29849482	Rocha Loures, M. A	2018	Spondyloarthritis (SpA)	Mixed	Case-control study				IL17F	rs763780 T/C	T/C
29849482	Rocha Loures, M. A	2018	Spondyloarthritis (SpA)	Mixed	Case-control study	67	210	277	IL17A	rs2275913 G/A	G/A
29849482	Rocha Loures, M. A	2018	Spondyloarthritis (SpA)	Mixed	Case-control study				IL17F	rs763780 T/C	T/C
29970680	Samiei, G	2018	Colorectal cance (CRC)	Asian	Case-control study	70	80	150	IL17A	rs2275913 G/A	G/A
29970680	Samiei, G	2018	Colorectal cance (CRC)	Asian	Case-control study				IL17F	rs763780 T/C	T/C
29985710	Poomarimuthu, M	2018	Rheumatic heart disease (RHD)	Other	Case-control study	99	126	225	IL17A	rs2275913 G/A	G/A
29985710	Poomarimuthu, M	2018	Rheumatic heart disease (RHD)	Other	Case-control study				IL17F	rs763780 T/C	T/C
30036556	Liang, T	2018	Asthma	Asian	Case-control study	221	223	444	IL17A	rs3748067 C/T	C/T
30036556	Liang, T	2018	Asthma	Asian	Case-control study				IL17F	rs2397084 T/C	T/C
30036556	Liang, T	2018	Asthma	Asian	Case-control study				IL17F	rs763780 T/C	T/C
30138899	Subbanna, M	2018	Schizophrenia	Other	Case-control study				IL17A	rs2275913 G/A	G/A
30196825	Debnath, M	2018	Guillain-Barré syndrome	Other	Case-control study	220	205	425	IL17A	rs2275913 G/A	G/A

			(GBS)								
30208882	Sode, J	2018	Ankylosing spondylitis (AS)	Caucasian	Case-control study	709	795	1504	IL17A	rs2275913 G/A	G/A
30233234	Al Obeed, O. A	2018	Colorectal cance (CRC)	Mixed	Case-control study	117	100	217	IL17A	rs2275913 G/A	G/A
30233234	Al Obeed, O. A	2018	Colorectal cance (CRC)	Mixed	Case-control study				IL17F	rs763780 T/C	T/C
30303246	Aquino, J. S	2019	Leprosy	Mixed	Case-control study	32	150	182	IL17A	rs2275913 G/A	G/A
30303246	Aquino, J. S	2019	Leprosy	Mixed	Case-control study	118	150	268	IL17F	rs763780 T/C	T/C
30303246	Aquino, J. S	2019	Leprosy	Mixed	Case-control study				IL17A	rs2275913 G/A	G/A
30303246	Aquino, J. S	2019	Leprosy	Mixed	Case-control study				IL17F	rs763780 T/C	T/C
30398030	Montúfar Robles, I	2019	Rheumatoid arthritis (RA)	Mixed	Case-control study	501	499	1000	IL17A	rs8193037 G/A	G/A
30398030	Montúfar Robles, I	2019	Rheumatoid arthritis (RA)	Mixed	Case-control study				IL17A	rs2275913 G/A	G/A
30398030	Montúfar Robles, I	2019	Rheumatoid arthritis (RA)	Mixed	Case-control study				IL17A	rs3819024 A/G	A/G
30398030	Montúfar Robles, I	2019	Rheumatoid arthritis (RA)	Mixed	Case-control study				IL17A	rs8193036 C/T	C/T
30398030	Montúfar Robles, I	2019	Systemic lupus erythematosus (SLE)	Mixed	Case-control study	367	499	866	IL17A	rs8193037 G/A	G/A
30398030	Montúfar Robles, I	2019	Systemic lupus erythematosus (SLE)	Mixed	Case-control study				IL17A	rs2275913 G/A	G/A
30398030	Montúfar Robles, I	2019	Systemic lupus erythematosus (SLE)	Mixed	Case-control study				IL17A	rs3819024 A/G	A/G
30398030	Montúfar Robles, I	2019	Systemic lupus erythematosus (SLE)	Mixed	Case-control study				IL17A	rs8193036 C/T	C/T
30399422	El Sharkawi, F. Z	2019	Multiple sclerosis (MS)	African	Case-control study	49	95	144	IL17F	rs763780 T/C	T/C
30399422	El Sharkawi, F. Z	2019	Multiple sclerosis (MS)	African	Case-control study	34	95	129	IL17F	rs763780 T/C	T/C
30446888	Aguín, N	2020	Prostate cancer (PCa)	Caucasian	Case-control study	241	192	433	IL17A	rs2275913 G/A	G/A
30594845	Tolba, F. M	2019	Immune thrombocytopenia (ITP)	African	Case-control study	63	112	175	IL17F	rs763780 T/C	T/C
30594845	Tolba, F. M	2019	Immune thrombocytopenia (ITP)	African	Case-control study	42	112	154	IL17F	rs763780 T/C	T/C
30655311	Xie, M	2019	Acute respiratory distress syndrome (ARDS)	Asian	Case-control study	210	210	420	IL17F	rs763780 T/C	T/C
30655311	Xie, M	2019	Acute respiratory distress syndrome (ARDS)	Asian	Case-control study				IL17A	rs2275913 G/A	G/A
30655311	Xie, M	2019	Acute respiratory distress	Asian	Case-control study				IL17A	rs8193036 C/T	C/T

			syndrome (ARDS)								
30658595	Bai, Y	2019	Osteoarthritis (OA)	Asian	Case-control study	594	576	1170	IL17A	rs2275913 G/A	G/A
30658595	Bai, Y	2019	Osteoarthritis (OA)	Asian	Case-control study				IL17F	rs763780 T/C	T/C
30666004	Kaur, R	2018	Psoriasis	Other	Case-control study	166	150	316	IL17A	rs10484879 C/A	C/A
30666004	Kaur, R	2018	Psoriasis	Other	Case-control study				IL17F	rs763780 T/C	T/C
30848577	Hesampour, F	2019	Polycystic ovary syndrome (PCOS)	Caucasian	Case-control study	150	150	300	IL17A	rs2275913 G/A	G/A
30851127	Keramat, F	2019	Brucellosis	Caucasian	Case-control study	86	86	172	IL17A	rs4711998 A/G	A/G
30851127	Keramat, F	2019	Brucellosis	Caucasian	Case-control study				IL17A	rs8193036 C/T	C/T
30851127	Keramat, F	2019	Brucellosis	Caucasian	Case-control study				IL17A	rs3819024 A/G	A/G
30851127	Keramat, F	2019	Brucellosis	Caucasian	Case-control study				IL17A	rs2275913 G/A	G/A
30851127	Keramat, F	2019	Brucellosis	Caucasian	Case-control study				IL17A	rs3819025 G/A	G/A
30851127	Keramat, F	2019	Brucellosis	Caucasian	Case-control study				IL17A	rs8193038 A/G	A/G
30851127	Keramat, F	2019	Brucellosis	Caucasian	Case-control study				IL17A	rs3804513 A/T	A/T
30851127	Keramat, F	2019	Brucellosis	Caucasian	Case-control study				IL17A	rs1974226 C/T	C/T
30851127	Keramat, F	2019	Brucellosis	Caucasian	Case-control study				IL17A	rs3748067 C/T	C/T
30896662	Jiang, L	2019	Osteoarthritis (OA)	Asian	Case-control study	410	507	917	IL17A	rs2275913 G/A	G/A
30896662	Jiang, L	2019	Osteoarthritis (OA)	Asian	Case-control study				IL17F	rs763780 T/C	T/C
30898714	Pasha, H. F	2019	Systemic lupus erythematosus (SLE)	African	Case-control study	80	80	160	IL17A	rs2275913 G/A	G/A
31074535	Zhao, Q	2019	Coronary artery disease (CAD)	Asian	Case-control study	191	131	322	IL17A	rs8193036 C/T	C/T
31074535	Zhao, Q	2019	Coronary artery disease (CAD)	Asian	Case-control study				IL17A	rs2275913 G/A	G/A
31074535	Zhao, Q	2019	Coronary artery disease (CAD)	Asian	Case-control study				IL17A	rs3748067 C/T	C/T
31074535	Zhao, Q	2019	Coronary artery disease (CAD)	Asian	Case-control study				IL17F	rs763780 T/C	T/C
31144366	Bafrani, H. H	2019	Osteoarthritis (OA)	Caucasian	Case-control study	127	127	254	IL17A	rs2275913 G/A	G/A
31144366	Bafrani, H. H	2019	Osteoarthritis (OA)	Caucasian	Case-control study				IL17F	rs763780 T/C	T/C
31144366	Bafrani, H. H	2019	Osteoarthritis (OA)	Caucasian	Case-control study				IL17F	rs2397084 T/C	T/C
31171891	Yu, Z. Q	2019	Intestinal tuberculosis (ITB)	Asian	Case-control study	133	500	633	IL17A	rs8193036 C/T	C/T

31171891	Yu, Z. Q	2019	Inflammatory Bowel Disease (IBD)	Asian	Case-control study	128	500	628	IL17A	rs8193036 C/T	C/T
31196204	Keshavarz, M	2019	Influenza A (H1N1)	Caucasian	Case-control study	50	96	146	IL17A	rs2275913 G/A	G/A
31196204	Keshavarz, M	2019	Influenza B	Caucasian	Case-control study	30	96	126	IL17A	rs2275913 G/A	G/A
31198448	Elsissy, M	2019	Acute Myeloid Leukaemia (AML)	African	Case-control study	100	100	200	IL17A	rs2275913 G/A	G/A
31198448	Elsissy, M	2019	Acute Myeloid Leukaemia (AML)	African	Case-control study				IL17F	rs763780 T/C	T/C
31230447	Ramzi, M	2019	Torque teno virus infection	Caucasian	Cross-sectional study	27	45	72	IL17A	rs2275913 G/A	G/A
31280929	Carnt, N. A	2019	Microbial keratitis (MK)	Caucasian	Case-control study	88	185	273	IL17A	rs2275913 G/A	G/A
31280929	Carnt, N. A	2019	Microbial keratitis (MK)	Caucasian	Case-control study				IL17F	rs2397084 T/C	T/C
31344596	Gonçalves de Albuquerque, S. D. C	2019	Cutaneous leishmaniasis (CL)	Mixed	Case-control study	172	173	345	IL17A	rs2275913 G/A	G/A
31464097	Jaramillo Valverde, L	2019	Guillain-Barré syndrome (GBS)	Mixed	Case-control study	9	11	20	IL17F	rs2397084 T/C	T/C
31682719	Feng, H	2019	Colorectal cance (CRC)	Asian	Case-control study	352	433	785	IL17A	rs2275913 G/A	G/A
31682719	Feng, H	2019	Colorectal cance (CRC)	Asian	Case-control study				IL17F	rs763780 T/C	T/C
31745628	Sabry, D	2020	Psoriasis	African	Case-control study	100	100	200	IL17RA	rs4819554 G/A	G/A
31831764	Choi, B. G	2019	Psoriasis	Asian	Case-control study	116	97	213	IL17F	rs763780 T/C	T/C
31845023	Dimberg, J	2020	Acute appendicitis (AA)	Caucasian	Case-control study	100	243	343	IL17A	rs2275913 G/A	G/A
31864094	Wang, W	2020	Tuberculosis (TB)	Asian	Case-control study	926	948	1874	IL17A	rs8193036 C/T	C/T
31864094	Wang, W	2020	Tuberculosis (TB)	Asian	Case-control study				IL17A	rs8193039 G/A	G/A
31864094	Wang, W	2020	Tuberculosis (TB)	Asian	Case-control study				IL17A	rs3804513 A/G	A/G
31864094	Wang, W	2020	Tuberculosis (TB)	Asian	Case-control study				IL17A	rs4711998 A/G	A/G
31964947	Ponce Gallegos, M. A	2020	Chronic obstructive pulmonary disease (COPD)	Mixed	Case-control study	436	657	1093	IL17A	rs2275913 G/A	G/A
31964947	Ponce Gallegos, M. A	2020	Chronic obstructive pulmonary disease (COPD)	Mixed	Case-control study	190	183	373	IL17A	rs2275913 G/A	G/A
31964947	Ponce Gallegos, M. A	2020	Chronic obstructive pulmonary disease (COPD)	Mixed	Case-control study				IL17A	rs2275913 G/A	G/A
31964947	Ponce Gallegos, M.	2020	Chronic obstructive	Mixed	Case-control study				IL17A	rs2275913 G/A	G/A

	A		pulmonary disease (COPD)								
31964947	Ponce Gallegos, M. A	2020	Chronic obstructive pulmonary disease (COPD)	Mixed	Case-control study				IL17A	rs8193036 C/T	C/T
31964947	Ponce Gallegos, M. A	2020	Chronic obstructive pulmonary disease (COPD)	Mixed	Case-control study				IL17A	rs8193036 C/T	C/T
31964947	Ponce Gallegos, M. A	2020	Chronic obstructive pulmonary disease (COPD)	Mixed	Case-control study				IL17A	rs8193036 C/T	C/T
31964947	Ponce Gallegos, M. A	2020	Chronic obstructive pulmonary disease (COPD)	Mixed	Case-control study				IL17A	rs8193036 C/T	C/T
32138639	Rahimzadeh, M	2020	Acute kidney injury (AKI)	Caucasian	Case-control study	65	70	135	IL17A	rs2275913 G/A	G/A
32138639	Rahimzadeh, M	2020	Acute kidney injury (AKI)	Caucasian	Case-control study				IL17A	rs3819024 A/G	A/G
32185238	Chen, A	2020	Preeclampsia (PE)	Asian	Case-control study	631	720	1351	IL17A	rs2275913 G/A	G/A
32185238	Chen, A	2020	Preeclampsia (PE)	Asian	Case-control study				IL17F	rs763780 T/C	T/C
32185238	Chen, A	2020	Preeclampsia (PE)	Asian	Case-control study				IL17RA	rs4819554 G/A	G/A
32242803	López Mejías, R	2020	Immunoglobulin-A vasculitis (IgAV)	Caucasian	Case-control study	360	1003	1363	IL17A	rs4711998 A/G	A/G
32242803	López Mejías, R	2020	Immunoglobulin-A vasculitis (IgAV)	Caucasian	Case-control study				IL17A	rs8193036 C/T	C/T
32242803	López Mejías, R	2020	Immunoglobulin-A vasculitis (IgAV)	Caucasian	Case-control study				IL17A	rs3819024 A/G	A/G
32242803	López Mejías, R	2020	Immunoglobulin-A vasculitis (IgAV)	Caucasian	Case-control study				IL17A	rs2275913 G/A	G/A
32242803	López Mejías, R	2020	Immunoglobulin-A vasculitis (IgAV)	Caucasian	Case-control study				IL17A	rs7747909 G/A	G/A
32294519	Tayefinasrabadi, H	2020	Hepatitis B Virus (HBV)	Caucasian	Case-control study	199	172	371	IL17F	rs763780 T/C	T/C
32294519	Tayefinasrabadi, H	2020	Hepatitis B Virus (HBV)	Caucasian	Case-control study				IL17F	rs2397084 T/C	T/C
32294519	Tayefinasrabadi, H	2020	Hepatitis B Virus (HBV)	Caucasian	Case-control study				IL17A	rs2275913 G/A	G/A
32294519	Tayefinasrabadi, H	2020	Hepatitis B Virus (HBV)	Caucasian	Case-control study				IL17A	rs10484879 C/A	C/A
32355229	Tabatabaei Panah, P. S	2020	Alopecia areata	Caucasian	Case-control study	60	60	120	IL17A	rs2275913 G/A	G/A
32380889	Elkoumi, M. A	2020	Systemic lupus erythematosus (SLE)	African	Case-control study	320	320	640	IL17A	rs2275913 G/A	G/A

32380889	Elkoumi, M. A	2020	Systemic lupus erythematosus (SLE)	African	Case-control study				IL17A	rs8193036 C/T	C/T
32380889	Elkoumi, M. A	2020	Systemic lupus erythematosus (SLE)	African	Case-control study				IL17A	rs3748067 C/T	C/T
32425678	Krajewski, W	2020	Bladder Cancer	Caucasian	Case-control study	175	207	382	IL17A	rs2275913 G/A	G/A
32425678	Krajewski, W	2020	Bladder Cancer	Caucasian	Case-control study				IL17F	rs763780 T/C	T/C
32522475	Chen, S	2020	Anti-E alloantibody production	Asian	Case-control study	95	186	281	IL17F	rs763780 T/C	T/C
32537881	Zhao, T	2020	Osteoarthritis (OA)	Asian	Case-control study	796	1854	2650	IL17F	rs763780 T/C	T/C
32537881	Zhao, T	2020	Osteoarthritis (OA)	Asian	Case-control study				IL17F	rs12201582 C/A	C/A
32537881	Zhao, T	2020	Osteoarthritis (OA)	Asian	Case-control study				IL17F	rs12203736 G/A	G/A
32537881	Zhao, T	2020	Osteoarthritis (OA)	Asian	Case-control study				IL17F	rs722323 G/A	G/A
32537881	Zhao, T	2020	Osteoarthritis (OA)	Asian	Case-control study				IL17F	rs117507014 T/C	T/C
32537881	Zhao, T	2020	Osteoarthritis (OA)	Asian	Case-control study				IL17F	rs1266828 G/A	G/A
32537881	Zhao, T	2020	Osteoarthritis (OA)	Asian	Case-control study				IL17F	rs2294835 A/G	A/G
32543936	Nisar, H	2021	Rheumatoid arthritis (RA)	Caucasian	Case-control study	150	160	310	IL17F	rs763780 T/C	T/C
32543936	Nisar, H	2021	Rheumatoid arthritis (RA)	Caucasian	Case-control study				IL17A	rs2275913 G/A	G/A
32582791	Ghaznavi, H	2020	Coronary artery disease (CAD)	Caucasian	Case-control study	220	220	440	IL17A	rs2275913 G/A	G/A
32630798	Borilova Linhartova, P	2020	Periodontitis	Caucasian	Case-control study	91	210	301	IL17A	rs2275913 G/A	G/A
32654355	Lauhkonen, E	2021	Bronchiolitis	Caucasian	Case-control study	266	99	365	IL17RA	rs4819554 G/A	G/A
32946631	Korppi, M	2021	Bacillus Calmette-Guérin (BCG) osteitis	Caucasian	Case-control study	132	99	231	IL17F	rs763780 T/C	T/C
32946631	Korppi, M	2021	Bacillus Calmette-Guérin (BCG) osteitis	Caucasian	Case-control study				IL17F	rs11465553 C/T	C/T
32946631	Korppi, M	2021	Bacillus Calmette-Guérin (BCG) osteitis	Caucasian	Case-control study				IL17F	rs7741835 C/T	C/T
32950026	Coto, E	2020	Coronary artery disease (CAD)	Caucasian	Case-control study	450	390	840	IL17RA	rs4819554 G/A	G/A
32969586	Nada, H	2021	Chronic spontaneous urticaria (CSU)	African	Case-control study	70	30	100	IL17RA	rs4819554 G/A	G/A

32969586	Nada, H	2021	Chronic spontaneous urticaria (CSU)	African	Case-control study				IL17RA	rs879577 C/T	C/T
33232805	Wu, W	2021	Hepatocellular carcinoma (HCC)	Asian	Case-control study	886	1086	1972	IL17A	rs4711998 A/G	A/G
33323002	Zhang, S	2020	Colorectal cance (CRC)	Asian	Case-control study	208	312	520	IL17A	rs2275913 G/A	G/A
33340282	Tabatabaei Panah, P. S	2020	Bullous pemphigoid (BP)	Caucasian	Case-control study	40	40	80	IL17A	rs2275913 G/A	G/A
33340282	Tabatabaei Panah, P. S	2020	Bullous pemphigoid (BP)	Caucasian	Case-control study				IL17A	rs3819025 G/A	G/A
33340282	Tabatabaei Panah, P. S	2020	Bullous pemphigoid (BP)	Caucasian	Case-control study				IL17F	rs2397084 T/C	T/C
33340282	Tabatabaei Panah, P. S	2020	Bullous pemphigoid (BP)	Caucasian	Case-control study				IL17F	rs763780 T/C	T/C
33340282	Tabatabaei Panah, P. S	2020	Bullous pemphigoid (BP)	Caucasian	Case-control study				IL17RA	rs2229151 G/A	G/A
33351682	Xiang, H	2020	Recurrent aphthous ulcer (RAU)	Asian	Case-control study	125	116	241	IL17A	rs2275913 G/A	G/A
33351682	Xiang, H	2020	Recurrent aphthous ulcer (RAU)	Asian	Case-control study				IL17F	rs763780 T/C	T/C
33364812	Li, W	2020	Vulvovaginal candidiasis (VVC)	Asian	Case-control study	213	326	539	IL17A	rs2275913 G/A	G/A
33364812	Li, W	2020	Vulvovaginal candidiasis (VVC)	Asian	Case-control study				IL17F	rs763780 T/C	T/C
33387966	Amin, A	2021	Rheumatoid arthritis (RA)	Caucasian	Case-control study	50	50	100	IL17A	rs2275913 G/A	G/A
33387966	Amin, A	2021	Rheumatoid arthritis (RA)	Caucasian	Case-control study				IL17F	rs763780 T/C	T/C
33387966	Amin, A	2021	Rheumatoid arthritis (RA)	Caucasian	Case-control study				IL17F	rs2397084 T/C	T/C
33530501	Mazurek Mochol, M	2021	Periodontitis	Caucasian	Case-control study	200	160	360	IL17A	rs2275913 G/A	G/A
33530501	Mazurek Mochol, M	2021	Periodontitis	Caucasian	Case-control study				IL17F	rs763780 T/C	T/C
33548976	Fouad, N. A	2020	Psoriasis	African	Case-control study	60	60	120	IL17F	rs763780 T/C	T/C
33818451	Khatonier, R	2020	Kala-azar	Other	Case-control study	76	133	209	IL17A	rs2275913 G/A	G/A
33818451	Khatonier, R	2020	Kala-azar	Other	Case-control study				IL17A	rs8193036 C/T	C/T

34200121	Neves, J. S. F	2021	Spondyloarthritis (SpA)	Mixed	Case-control study	100	182	282	IL17A	rs2275913 G/A	G/A
34200121	Neves, J. S. F	2021	Spondyloarthritis (SpA)	Mixed	Case-control study				IL17A	rs2275913 G/A	G/A
34200121	Neves, J. S. F	2021	Spondyloarthritis (SpA)	Mixed	Case-control study	100	184	284	IL17F	rs763780 T/C	T/C
34200121	Neves, J. S. F	2021	Spondyloarthritis (SpA)	Mixed	Case-control study				IL17F	rs763780 T/C	T/C
34384609	Bertol, B. C	2021	Papillary thyroid cancer (PTC)	Mixed	Case-control study	188	170	358	IL17A	rs2275913 G/A	G/A
34384609	Bertol, B. C	2021	Papillary thyroid cancer (PTC)	Mixed	Case-control study	187	168	355	IL17RA	rs4819554 G/A	G/A
34514065	Pk, S. K	2021	Periodontitis	Other	Case-control study	15	15	30	IL17A	rs2275913 G/A	G/A
34514065	Pk, S. K	2021	Periodontitis	Other	Case-control study				IL17A	rs2275913 G/A	G/A
34514065	Pk, S. K	2021	Type 1 diabetes mellitus (T1DM)	Other	Case-control study				IL17A	rs2275913 G/A	G/A
34514065	Pk, S. K	2021	Type 1 diabetes mellitus (T1DM)	Other	Case-control study				IL17A	rs2275913 G/A	G/A
34554306	Hristova, M	2021	Systemic lupus erythematosus (SLE)	Caucasian	Case-control study	59	95	154	IL17A	rs2275913 G/A	G/A
34554306	Hristova, M	2021	Systemic lupus erythematosus (SLE)	Caucasian	Case-control study				IL17RC	rs708567 C/T	C/T
34554386	Gomes, I. B	2021	Keratoconus (KC)	Mixed	Case-control study	35	61	96	IL17A	rs2275913 G/A	G/A
34554386	Gomes, I. B	2021	Keratoconus (KC)	Mixed	Case-control study				IL17F	rs763780 T/C	T/C

Note: A, adenine; C, cytosine; G, guanine; T, thymine;

Supplementary Table 2: Associations between variants in the IL17 famliy genes associated with risk of human disease in meta-analysis under additive model (at least 3 datasets).

ID	Gene	Variant	Alleles[a]	Disease	Disease type	Ethnicity	MAF[b]	Number Evaluation: Studies	Number Evaluation: Cases	Number Evaluation: Controls	Risk of Meta-Analysis: OR (95%CI)	Risk of Meta-Analysis: P_{value}	Heterogeneity: I^2 (%)	Heterogeneity: P_Q	Venice Criteria[c]	Venice Criteria Grade	Amount of Evidence: N_{minor}	Amount of Evidence: Grade	Replication	Protection from Bias: Grade	Protection from Bias: Reasons for Bias	Reason for bias Exemption	Initial study influence: OR (95%CI)	Initial study influence: P_{value}	Deviation from HWE	P_{egger}	P_{begger}	P_{excess}	Low OR	FPRP values[d]	Credibility of Evidence[e]
**Asthma**																															
1	IL17F	rs1889570	C/T	Asthma		Overall	0.3826	3	1275	1376	1.109 (0.888-1.384)	0.361	73.5	0.023																	
1	IL17F	rs1889570	C/T	Asthma		Asian	0.3235	2	856	983	1.230 (1.075-1.401)	0.003	32.9	0.222	ABA	Moderate	1250	A	B	A	No	NA	1.329 (1.108-1.594)	<0.001	No	NA	1.000	NA	No	0.032	Strong
1	IL17F	rs1889570	C/T	Asthma		Other	0.5305	1	419	393	0.915 (0.753-1.112)	0.371	NA	NA																	
2	IL17A	rs2275913	G/A	Asthma		Overall	0.3527	6	1051	1741	0.946 (0.690-1.296)	0.728	78.8	< 0.001																	
2	IL17A	rs2275913	G/A	Asthma		Asian	0.4277	3	774	883	0.976 (0.681-1.400)	0.896	78.7	0.009																	
2	IL17A	rs2275913	G/A	Asthma		Caucasian	0.2331	3	277	534	0.891 (0.419-1.896)	0.765	85.9	0.001																	
3	IL17F	rs2397084	T/C	Asthma		Overall	0.0230	3	415	565	3.220 (1.895-5.473)	< 0.001	0.0	0.965	CAA	Weak	65	C	A	A	No	NA	3.362 (1.744-6.478)	<0.001	No	0.823	1.000	1.000	No	0.110	Weak
3	IL17F	rs2397084	T/C	Asthma		Asian	0.0045	1	221	223	3.572 (0.738-17.292)	0.114	NA	NA																	
3	IL17F	rs2397084	T/C	Asthma		Caucasian	0.0351	2	194	342	3.167 (1.805-5.556)	< 0.001	0.0	0.826																	
4	IL17F	rs763780	T/C	Asthma		Overall	0.1060	7	1714	2032	0.925 (0.670-1.277)	0.635	71.6	0.002																	
4	IL17F	rs763780	T/C	Asthma		Asian	0.1050	5	1520	1690	1.039 (0.743-1.452)	0.824	74.5	0.003																	
4	IL17F	rs763780	T/C	Asthma		Caucasian	0.0848	2	194	342	0.536 (0.294-0.979)	0.042	0.0	0.363	CAA	Weak	72	C	A	A	No	NA	0.419 (0.188-0.935)	<0.001	No	NA	1.000	NA	No	0.771	Weak
5	IL17A	rs8193036	C/T	Asthma		Asian	0.2856	3	1087	1224	0.833 (0.721-0.962)	0.013	83.1	0.003	ACC	Weak	1127	A	C	C	excess of significant findings	NA	0.902 (0.733-1.111)	0.333	No	0.368	1.000	0.001	No	0.197	Weak
**Autoimmune thyroid diseases (AITD)**																															
6	IL17F	rs763780	T/C	Autoimmune thyroid diseases (AITD)		Asian	0.1161	3	1265	1189	1.623 (1.101-2.393)	0.014	73.8	0.022	BCC	Weak	667	B	C	C	small study, excess of significant findings	NA	1.543 (0.887-2.683)	0.125	No	0.029	0.295	1.000	No	0.444	Weak
**Cervical cancer**																															
7	IL17A	rs2275913	G/A	Cervical cancer		Asian	0.3086	6	1634	2235	1.391 (1.263-1.532)	< 0.001	0.0	0.617	AAA	Strong	2622	A	A	A	No	NA	1.413 (1.267-1.576)	<0.001	No	0.761	1.000	1.000	No	0.000	Strong
8	IL17A	rs3748067	C/T	Cervical cancer		Asian	0.1721	4	1017	1418	1.493 (1.112-2.015)	0.008	69.0	0.022	BCC	Weak	878	B	C	C	publication bias	NA	1.546 (1.019-2.345)	0.04	No	0.115	0.089	0.641	No	0.264	Weak
9	IL17F	rs763780	T/C	Cervical cancer		Asian	0.2329	5	1449	1865	1.184 (1.045-1.342)	0.008	7.7	0.363	AAA	Strong	1513	A	A	A	No	NA	1.226 (1.067-1.409)	0.004	No	0.118	0.327	0.266	No	0.135	Strong
**Chronic obstructive pulmonary disease (COPD)**																															
10	IL17A	rs2275913	G/A	COPD		Overall	0.2026	5	1404	1729	1.023 (0.778-1.344)	0.871	76.0	0.002																	
10	IL17A	rs2275913	G/A	COPD		Asian	0.4527	1	152	201	0.624 (0.455-0.875)	0.004	NA	NA																	
10	IL17A	rs2275913	G/A	COPD		Mixed	0.1695	4	1252	1528	1.163 (0.976-1.385)	0.091	30.1	0.231																	
11	IL17A	rs8193036	C/T	COPD		Overall	0.7273	5	1404	1729	1.070 (0.868-1.320)	0.525	65.1	0.022																	
11	IL17A	rs8193036	C/T	COPD		Asian	0.2985	1	152	201	1.448 (1.049-2.000)	0.025	NA	NA																	
11	IL17A	rs8193036	C/T	COPD		Mixed	0.7833	4	1252	1528	0.995 (0.820-1.208)	0.960	52.5	0.097																	
**Colorectal cancer (CRC)**																															
12	IL17A	rs2275913	G/A	Colorectal cancer		Overall	0.2714	7	1345	1535	1.452 (1.178-1.790)	< 0.001	65.1	0.009	ACC	Weak	1693	A	C	C	publication bias	NA	1.417 (1.129-1.780)	0.003	No	0.124	0.035	0.706	No	0.014	Moderate
12	IL17A	rs2275913	G/A	Colorectal cancer		Asian	0.3250	3	630	825	1.524 (1.155-2.011)	0.003	63.0	0.067																	
12	IL17A	rs2275913	G/A	Colorectal cancer		Caucasian	0.2120	3	598	610	1.254 (0.871-1.805)	0.223	69.8	0.036																	
12	IL17A	rs2275913	G/A	Colorectal cancer		Mixed	0.1850	1	117	100	2.038 (1.298-3.198)	0.002	NA	NA																	
13	IL17F	rs763780	T/C	Colorectal cancer		Overall	0.1880	6	1841	1307	1.267 (0.933-1.720)	0.130	58.9	0.033																	
13	IL17F	rs763780	T/C	Colorectal cancer		Asian	0.1417	3	1422	867	1.270 (0.825-1.956)	0.278	77.1	0.011																	
13	IL17F	rs763780	T/C	Colorectal cancer		Caucasian	0.0754	2	302	340	1.403 (0.655-3.004)	0.384	65.8	0.087																	
13	IL17F	rs763780	T/C	Colorectal cancer		Mixed	0.9700	1	117	100	1.003 (0.331-3.035)	0.996	NA	NA																	
**Coronary artery disease (CAD)**																															
14	IL17A	rs2275913	G/A	Coronary artery disease		Overall	0.3293	8	3654	3298	1.179 (1.026-1.354)	0.020	69.4	0.002	ACA	Weak	4736	A	C	A	No	NA	1.214 (1.033-1.428)	0.019	No	0.128	0.326	0.277	No	0.272	Weak
14	IL17A	rs2275913	G/A	Coronary artery disease		Asian	0.3178	6	2534	2411	1.197 (1.053-1.360)	0.006	52.1	0.064																	
14	IL17A	rs2275913	G/A	Coronary artery disease		Caucasian	0.2841	1	220	220	1.527 (1.150-2.026)	0.003	NA	NA																	
14	IL17A	rs2275913	G/A	Coronary artery disease		Mixed	0.1904	1	900	667	0.870 (0.724-1.045)	0.137	NA	NA																	
15	IL17A	rs3748067	C/T	Coronary artery disease		Asian	0.6603	4	1197	1170	0.723 (0.572-0.913)	0.006	55.3	0.082	ACA	Weak	2934	A	C	A	No	NA	0.697 (0.508-0.957)	0.026	No	0.483	1.000	0.374	No	0.140	Weak
**Functional dyspepsia (FD)**																															
16	IL17A	rs2275913	G/A	Functional dyspepsia		Asian	0.3936	3	175	564	0.815 (0.632-1.052)	0.116	0.0	0.943																	
17	IL17F	rs2397084	T/C	Functional dyspepsia		Other	0.0860	3	237	695	0.885 (0.290-2.702)	0.831	79.1	0.008																	
18	IL17F	rs763780	T/C	Functional dyspepsia		Overall	0.1178	6	412	1314	0.891 (0.688-1.154)	0.383	0.0	0.667																	
18	IL17F	rs763780	T/C	Functional dyspepsia		Asian	0.1257	3	175	564	0.676 (0.447-1.022)	0.063	0.0	0.998																	
18	IL17F	rs763780	T/C	Functional dyspepsia		Other	0.1120	3	237	750	1.089 (0.779-1.522)	0.617	0.0	0.952																	
**Gastric cancer (GC)**																															
19	IL17A	rs2275913	G/A	Gastric Cancer		Overall	0.3845	18	6207	8902	1.281 (1.179-1.392)	< 0.001	60.5	< 0.001	ACA	Weak	12062	A	C	A	No	NA	1.276 (1.170-1.392)	<0.001	No	0.015	0.173	0.815	No	0.000	Moderate
19	IL17A	rs2275913	G/A	Gastric Cancer		Asian	0.3855	17	6046	8731	1.264 (1.165-1.372)	< 0.001	58.4	0.001																	
19	IL17A	rs2275913	G/A	Gastric Cancer		Caucasian	0.3333	1	161	171	1.723 (1.258-2.358)	0.001	NA	NA																	
20	IL17A	rs3748067	C/T	Gastric Cancer		Asian	0.3385	11	3022	5284	0.981 (0.750-1.283)	0.889	83.6	< 0.001																	
21	IL17A	rs4711998	A/G	Gastric Cancer		Asian	0.3316	3	1750	2066	1.001 (0.906-1.105)	0.989	41.7	0.180																	
22	IL17F	rs763780	T/C	Gastric Cancer		Asian	0.6781	10	3944	4566	1.042 (0.805-1.348)	0.756	87.4	< 0.001																	
**Hepatitis B Virus (HBV) infection**																															
23	IL17A	rs2275913	G/A	Hepatitis B Virus (HBV) infection		Overall	0.4986	13	2633	2479	0.968 (0.834-1.124)	0.671	68.4	< 0.001																	
23	IL17A	rs2275913	G/A	Hepatitis B Virus (HBV) infection		Asian	0.5085	12	2434	2307	0.964 (0.821-1.133)	0.660	70.7	< 0.001																	
23	IL17A	rs2275913	G/A	Hepatitis B Virus (HBV) infection		Caucasian	0.3663	1	199	172	1.024 (0.760-1.381)	0.875	NA	NA																	
24	IL17A	rs4711998	A/G	Hepatitis B Virus (HBV) infection		Asian	0.2324	4	533	617	1.068 (0.678-1.683)	0.776	81.9	0.001																	
25	IL17F	rs763780	T/C	Hepatitis B Virus (HBV) infection		Overall	0.1727	7	1445	1546	0.852 (0.739-0.982)	0.027	82.0	< 0.001	BBC	Weak	697	B	C	C	excess of significant findings	NA	0.788 (0.674-0.922)	0.003	No	0.285	1.000	0.065	No	0.082	Weak
25	IL17F	rs763780	T/C	Hepatitis B Virus (HBV) infection		Asian	0.1727	6	1246	1374	0.826 (0.713-0.957)	0.011	83.8	< 0.001																	
25	IL17F	rs763780	T/C	Hepatitis B Virus (HBV) infection		Caucasian	0.0610	1	199	172	1.345 (0.760-2.379)	0.309	NA	NA																	
26	IL17A	rs8193036	C/T	Hepatitis B Virus (HBV) infection		Asian	0.2656	6	1260	945	0.947 (0.707-1.269)	0.717	74.7	0.001																	
**Hepatocellular carcinoma (HCC)**																															
27	IL17A	rs2275913	G/A	Hepatocellular carcinoma (HCC)		Asian	0.5100	4	462	450	1.108 (0.755-1.626)	0.600	72.9	0.011																	
**Immune thrombocytopenia (ITP)**																															
28	IL17F	rs763780	T/C	Immune thrombocytopenia (ITP)		Overall	0.2721	6	568	748	0.683 (0.290-1.608)	0.382	89.5	< 0.001																	
28	IL17F	rs763780	T/C	Immune thrombocytopenia (ITP)		Asian	0.3186	3	413	473	0.486 (0.362-0.653)	< 0.001	0.0	0.966	CAC	Weak	529	C	A	C	small study	NA	0.489 (0.347-0.689)	<0.001	No	0.072	0.296	0.558	No	0.002	Moderate
28	IL17F	rs763780	T/C	Immune thrombocytopenia (ITP)		African	0.1916	3	155	274	0.975 (0.110-8.619)	0.382	94.0	< 0.001																	
**Inflammatory Bowel Disease (IBD)**																															
29	IL17A	rs2275913	G/A	Inflammatory Bowel Disease (IBD)		Asian	0.4202	3	580	764	1.051 (0.783-1.412)	0.740	71.1	0.031																	
30	IL17F	rs763780	T/C	Inflammatory Bowel Disease (IBD)		Overall	0.0840	5	1108	2928	0.877 (0.728-1.055)	0.164	51.9	0.081																	
30	IL17F	rs763780	T/C	Inflammatory Bowel Disease (IBD)		Asian	0.1531	3	393	994	0.750 (0.585-0.962)	0.024	51.9	0,125	CCC	Weak	397	C	C	C	HWE	NA	0.609 (0.437-0.848)	0.003	Yes	0.495	0.296	0.145	No	0.352	Weak
30	IL17F	rs763780	T/C	Inflammatory Bowel Disease (IBD)		Caucasian	0.0486	2	715	1934	1.081 (0.817-1.429)	0.587	0.0	0.417																	
31	IL17A	rs8193036	C/T	Inflammatory Bowel Disease (IBD)		Asian	0.2854	3	597	1016	1.012 (0.764-1.340)	0.936	65.1	0.057																	
**Leprosy**																															
32	IL17A	rs2275913	G/A	Leprosy		Mixed	0.2304	3	132	369	0.971 (0.726-1.299)	0.841	6.9	0.342																	
33	IL17F	rs763780	T/C	Leprosy		Overall	0.1791	5	444	537	0.364 (0.268-0.496)	< 0.001	80.8	< 0.001	CCC	Weak	257	C	C	C	first study	NA	0.586 (0.245-1.399)	0.229	No	0.122	0.462	1.000	No	0.000	Moderate
33	IL17F	rs763780	T/C	Leprosy		Mixed	0.0707	3	304	369	0.870 (0.537-1.409)	0.571	0.0	0.884																	
33	IL17F	rs763780	T/C	Leprosy		Other	0.4167	2	140	168	0.200 (0.131-0.303)	< 0.001	0.0	0.936																	
**Lung cancer**																															
34	IL17F	rs12203582	G/A	Lung cancer		Asian	0.5968	4	320	1432	1.103 (0.918-1.325)	0.293	0.0	0.790																	
35	IL17A	rs2275913	G/A	Lung cancer		Overall	0.3778	5	559	1690	1.213 (1.039-1.375)	0.014	0.0	0.918	AAA	Strong	1679	A	A	A	No	NA	1.260 (1.053-1.509)	0.012	No	0.593	0.462	0.596	No	0.046	Strong
35	IL17A	rs2275913	G/A	Lung cancer		Asian	0.4092	4	320	1432	1.260 (1.053-1.509)	0.012	0.0	0.963																	
35	IL17A	rs2275913	G/A	Lung cancer		Caucasian	0.2035	1	239	258	1.088 (0.802-1.477)	0.586	NA	NA																	
36	IL17A	rs3819024	A/G	Lung cancer		Asian	0.4426	3	322	1098	0.940 (0.784-1.127)	0.503	0.0	0.832																	
37	IL17A	rs8193037	G/A	Lung cancer		Asian	0.1120	3	322	1098	2.129 (1.677-2.702)	< 0.001	49.5	0.138	BBA	Moderate	384	B	B	A	No	NA	1.662 (1.174-2.351)	0.004	No	0.641	1.000	1.000	No	0.000	Strong
**Multiple sclerosis (MS)**																															
38	IL17F	rs763780	T/C	Multiple sclerosis (MS)		Overall	0.1414	4	774	1064	1.154 (0.608-2.193)	0.661	85.1	< 0.001																	
38	IL17F	rs763780	T/C	Multiple sclerosis (MS)		Asian	0.1386	2	691	874	0.687 (0.548-0.862)	0.001	0.0	0.884	BAA	Moderate	377	B	A	A	No	NA	0.691 (0.542-0.882)	<0.05	No	NA	1.000	NA	No	0.036	Strong
38	IL17F	rs763780	T/C	Multiple sclerosis (MS)		African	0.1737	2	83	190	2.064 (1.348-3.161)	0.001	0.0	0.682	BAA	M	116	B	A	A	No	NA	2.275 (1.210-4.297)	<0.05	No	NA	1.000	NA	No	0.187	M
**Osteoarthritis (OA)**																															
39	IL17A	rs2275913	G/A	Osteoarthritis (OA)		Overall	0.3448	6	1791	2324	1.232 (1.024-1.483)	0.027	71.7	0.003	ACA	Weak	3075	A	C	A	No	NA	1.175 (1.060-1.301)	0.002	No	0.463	0.260	0.688	No	0.347	Weak
39	IL17A	rs2275913	G/A	Osteoarthritis (OA)		Asian	0.3665	3	1306	1383	1.411 (1.105-1.804)	0.006	77.8	0.011																	
39	IL17A	rs2275913	G/A	Osteoarthritis (OA)		Caucasian	0.3130	3	485	941	1.048 (0.839-1.310)	0.680	38.8	0.195																	
40	IL17F	rs763780	T/C	Osteoarthritis (OA)		Overall	0.1088	7	2671	4420	1.344 (1.111-1.626)	0.002	57.1	0.030	ACA	Weak	1725	A	C	A	No	NA	1.352 (1.077-1.697)	0.009	No	0.735	0.764	0.463	No	0.049	Moderate
40	IL17F	rs763780	T/C	Osteoarthritis (OA)		Asian	0.1348	4	2102	3237	1.239 (1.026-1.497)	0.026	57.3	0.071																	
40	IL17F	rs763780	T/C	Osteoarthritis (OA)		Caucasian	0.0376	3	569	1138	1.754 (1.157-2.661)	0.008	39.3	0.193																	
**Periodontitis**																															
41	IL17A	rs2275913	G/A	Periodontitis		Overall	0.3253	11	935	971	1.304 (0.892-1.908)	0.171	83.0	< 0.001																	
41	IL17A	rs2275913	G/A	Periodontitis		Caucasian	0.3547	3	535	524	0.942 (0.782-1.134)	0.526	0.0	0.744																	
41	IL17A	rs2275913	G/A	Periodontitis		Mixed	0.2920	4	300	347	1.040 (0.514-2.103)	0.914	84.0	< 0.001																	
41	IL17A	rs2275913	G/A	Periodontitis		Other	0.2800	4	100	100	2.498 (0.993-6.235)	0.052	74.3	0.009																	
42	IL17F	rs763780	T/C	Periodontitis		Overall	0.0563	5	500	507	1.251 (0.840-1.860)	0.270	0.0	0.520																	
42	IL17F	rs763780	T/C	Periodontitis		Mixed	0.0656	4	300	347	1.506 (0.955-2.373)	0.078	0.0	0.935																	
42	IL17F	rs763780	T/C	Periodontitis		Caucasian	0.0375	1	200	160	0.658 (0.281-1.543)	0.336	NA	NA																	
**Pre-eclampsia (PE)**																															
43	IL17A	rs2275913	G/A	Periodontitis		Overall	0.4129	3	1923	2296	1.009 (0.919-1.108)	0.848	0.0	0.860																	
43	IL17A	rs2275913	G/A	Periodontitis		Asian	0.4111	2	1662	2018	1.020 (0.922-1.129)	0.702	0.0	0.896																	
43	IL17A	rs2275913	G/A	Periodontitis		Caucasian	0.4245	1	261	278	0.950 (0.745-1.210)	0.676	NA	NA																	
44	IL17F	rs763780	T/C	Periodontitis		Overall	0.0979	3	1923	2296	0.995 (0.852-1.162)	0.947	54.4	0.111																	
44	IL17F	rs763780	T/C	Periodontitis		Asian	0.1017	2	1662	2018	0.938 (0.793-1.109)	0.451	0.0	0.342																	
44	IL17F	rs763780	T/C	Periodontitis		Caucasian	0.0737	1	261	278	1.449 (0.948-2.217)	0.087	NA	NA																	
**Psoriasis**																															
45	IL17F	rs763780	T/C	Psoriasis		Overall	0.1219	6	1151	975	1.499 (0.899-2.499)	0.121	80.2	< 0.001																	
45	IL17F	rs763780	T/C	Psoriasis		Asian	0.0955	2	324	363	1.571 (1.118-2.207)	0.009	0.0	0.371	BAA	Moderate	157	B	A	A	No	NA	2.082 (1.028-4.214)	0.008	No	NA	1.000	NA	No	0.307	Weak
45	IL17F	rs763780	T/C	Psoriasis		Caucasian	0.0274	2	601	462	1.378 (0.811-2.343)	0.236	0.0	0.371																	
45	IL17F	rs763780	T/C	Psoriasis		African	0.0750	1	60	60	4.111 (1.856-9.105)	< 0.001	NA	NA																	
45	IL17F	rs763780	T/C	Psoriasis		Other	0.4567	1	166	150	0.676 (0.492-0.929)	0.016	NA	NA																	
**Recurrent Miscarriage (RM)**																															
46	IL17A	rs2275913	G/A	Recurrent Miscarriage (RM)		Overall	0.4725	3	290	309	1.148 (0.753-1.752)	0.520	65.6	0.055																	
46	IL17A	rs2275913	G/A	Recurrent Miscarriage (RM)		Caucasian	0.5265	2	170	189	0.924 (0.666-1.282)	0.634	0.0	0.451																	
46	IL17A	rs2275913	G/A	Recurrent Miscarriage (RM)		African	0.3875	1	120	120	1.634 (1.137-2.348)	0.008	NA	NA																	
**Rheumatoid arthritis (RA)**																															
47	IL17A	rs2275913	G/A	Rheumatoid arthritis (RA)		Overall	0.3661	13	3826	4011	0.862 (0.833-0.955)	0.001	6.8	0.378	AAA	Strong	5527	A	A	A	No	NA	0.904 (0.835-0.978)	0.012	No	0.948	0.951	0.681	No	0.001	Strong
47	IL17A	rs2275913	G/A	Rheumatoid arthritis (RA)		Asian	0.4633	1	615	839	0.875 (0.754-1.016)	0.080	NA	NA																	
47	IL17A	rs2275913	G/A	Rheumatoid arthritis (RA)		Caucasian	0.3908	8	2452	2389	0.896 (0.824-0.974)	0.010	27.4	0.209																	
47	IL17A	rs2275913	G/A	Rheumatoid arthritis (RA)		Mixed	0.2687	4	759	783	0.897 (0.744-1.082)	0.255	5.0	0.368																	
48	IL17F	rs2397084	T/C	Rheumatoid arthritis (RA)		Caucasian	0.1056	4	800	695	1.575 (0.499-4.968)	0.439	94.9	< 0.001																	
49	IL17A	rs3819024	A/G	Rheumatoid arthritis (RA)		Overall	0.3712	3	2053	2266	0.914 (0.834-1.002)	0.056	0.0	0.603																	
49	IL17A	rs3819024	A/G	Rheumatoid arthritis (RA)		Asian	0.4816	1	615	839	0.880 (0.758-1.021)	0.092	NA	NA																	
49	IL17A	rs3819024	A/G	Rheumatoid arthritis (RA)		Caucasian	0.3842	1	937	928	0.913 (0.800-1.043)	0.179	NA	NA																	
49	IL17A	rs3819024	A/G	Rheumatoid arthritis (RA)		Mixed	0.1513	1	501	499	1.019 (0.798-1.300)	0.882	NA	NA																	
50	IL17F	rs763780	T/C	Rheumatoid arthritis (RA)		Overall	0.0713	9	1297	1264	1.650 (0.792-3.437)	0.181	89.9	< 0.001																	
50	IL17F	rs763780	T/C	Rheumatoid arthritis (RA)		Caucasian	0.0751	6	1039	980	2.070 (0.794-5.395)	0.137	92.9	< 0.001																	
50	IL17F	rs763780	T/C	Rheumatoid arthritis (RA)		Mixed	0.0581	3	258	284	0.977 (0.527-1.812)	0.941	23.4	0.271																	
51	IL17A	rs8193036	C/T	Rheumatoid arthritis (RA)		Overall	0.3941	3	2052	2261	1.074 (0.905-1.276)	0.414	65.8	0.054																	
51	IL17A	rs8193036	C/T	Rheumatoid arthritis (RA)		Asian	0.2555	1	615	839	1.250 (1.056-1.479)	0.010	NA	NA																	
51	IL17A	rs8193036	C/T	Rheumatoid arthritis (RA)		Caucasian	0.2974	1	936	923	0.953 (0.827-1.097)	0.502	NA	NA																	
51	IL17A	rs8193036	C/T	Rheumatoid arthritis (RA)		Mixed	0.7986	1	501	499	1.050 (0.843-1.309)	0.662	NA	NA																	
**Spondyloarthritis (SpA)**																															
52	IL17A	rs2275913	G/A	Spondyloarthritis (SpA)		Mixed	0.2500	4	439	784	1.401 (1.156-1.698)	0.001	25.2	0.260	BBA	Moderate	641	B	B	A	No	NA	1.397 (1.097-1.778)	0.007	No	0.669	0.308	0.159	No	0.015	Strong
53	IL17F	rs763780	T/C	Spondyloarthritis (SpA)		Mixed	0.0522	4	439	788	3.684 (2.737-4.960)	< 0.001	0.0	0.431	BAA	Moderate	213	B	A	A	No	High consistent OR	3.781 (2.635-5.426)	<0.001	No	0.890	1.000	1.000	No	0.000	Strong
**Systemic lupus erythematosus (SLE)**																															
54	IL17A	rs2275913	G/A	Systemic lupus erythematosus (SLE)		Overall	0.2338	5	941	1289	1.160 (1.007-1.336)	0.040	53.5	0.072	ACA	Weak	1093	A	C	A	No	NA	1.218 (1.044-1.422)	0.012	No	0.764	0.806	0.100	No	0.429	Weak
54	IL17A	rs2275913	G/A	Systemic lupus erythematosus (SLE)		African	0.2807	3	515	695	1.281 (1.071-1.533)	0.007	63.2	0.066																	
54	IL17A	rs2275913	G/A	Systemic lupus erythematosus (SLE)		Caucasian	0.3105	1	59	95	0.975 (0.592-1.604)	0.920	NA	NA																	
54	IL17A	rs2275913	G/A	Systemic lupus erythematosus (SLE)		Mixed	0.1573	1	367	499	0.985 (0.758-1.281)	0.910	NA	NA																	
**Tuberculosis (TB)**																															
55	IL17A	rs2275913	G/A	Tuberculosis (TB)		Overall	0.4066	12	4240	4983	0.998 (0.854-1.165)	0.975	79.5	< 0.001																	
55	IL17A	rs2275913	G/A	Tuberculosis (TB)		Asian	0.4283	5	3137	3549	1.066 (0.894-1.270)	0.478	80.4	< 0.001																	
55	IL17A	rs2275913	G/A	Tuberculosis (TB)		Caucasian	0.4039	5	727	1052	1.120 (0.799-1.572)	0.511	79.4	0.001																	
55	IL17A	rs2275913	G/A	Tuberculosis (TB)		Mixed	0.2287	2	376	382	0.594 (0.399-0.884)	0.010	48.6	0.163	BBA	Moderate	256	B	B	A	No	NA	0.708 (0.501-0.999)	<0.05	No	NA	1.000	NA	No	0.406	Weak
56	IL17A	rs3748067	C/T	Tuberculosis (TB)		Asian	0.1429	3	2365	2305	1.244 (0.939-1.649)	0.129	71.4	0.030																	
57	IL17F	rs763780	T/C	Tuberculosis (TB)		Overall	0.0990	8	3574	4238	1.380 (1.084-1.758)	0.009	72.0	0.001	ACA	Weak	1732	A	C	A	No	NA	1.369 (1.016-1.844)	0.039	No	0.345	0.711	0.727	No	0.188	Weak
57	IL17F	rs763780	T/C	Tuberculosis (TB)		Asian	0.1139	5	3137	3549	1.426 (1.082-1.879)	0.012	0.0	0.665																	
57	IL17F	rs763780	T/C	Tuberculosis (TB)		Caucasian	0.0225	3	437	689	1.088 (0.607-1.951)	0.776	83.3	< 0.001																	
**Type 1 diabetes mellitus (T1DM)**																															
58	IL17A	rs2275913	G/A	T1DM		Overall	0.3288	3	155	184	1.223 (0.887-1.685)	0.219	31.4	0.233																	
58	IL17A	rs2275913	G/A	T1DM		Other	0.2333	2	30	30	0.580 (0.229-1.466)	0.249	0.0	0.782																	
58	IL17A	rs2275913	G/A	T1DM		Caucasian	0.3474	1	125	154	1.360 (0.965-1.918)	0.079	NA	NA																	

需要去掉

Note: OR, odds ratio; A, adenine; T, thymine; G, guanine; C, cytosine; NA: Not Available.
HWE: = Yes: Significance lost excluding studies with controls violating HWE; No: Significance was not lost excluding the study with controls violating HWE;
Low OR = Low magnitude of the association (i.e. 0.87<OR<1.15)
Publication bias = Possible existence of potential publication bias (Begg's test, p<0.10)
Small study = Possible existence of potential small study effect (modified Egger's test, p<0.10).
An excess of significant findings based on the excess significance test. (p< 0.10 is usually considered evidence for significant evidence of an excess of significant findings.)

[a]Major alleles/minor alleles.

[b]Frequency of minor allele in controls.

[c]Strength of epidemiological evidence based on the Venice criteria.

[d]The prior probability of FPRP is 0.05, and the FPRP level of noteworthiness is 0.20.

[e]Degree of epidemiological credibility based on the combination of results from Venice guidelines and FPRP tests.

Supplementary Table 3: Variants in IL-17 family genes showing no relation to risk of disease in meta-analyses in additive model (at least 3 datasets)

Gene	Variant	Alleles[a]	Disease	Ethnicity	MAF[b]	Number Evaluation			Risk of Meta-Analysis		Heterogeneity		Power (%)	the value of Power (%) if the MAF is 0.2	the value of power (%) if the MAF is 0.1
						Studies	Cases	Controls	OR (95%CI)	P_{value}	I^2(%)	P_Q			
**Asthma**															
IL17A	rs2275913	G/A	Asthma	Overall	0.3527	6	1051	1741	0.946 (0.690-1.296)	0.728	78.8	< 0.001	58.85	45.57	28.71
IL17A	rs2275913	G/A	Asthma	Asian	0.4277	3	774	883	0.976 (0.681-1.400)	0.896	78.7	0.009			
IL17A	rs2275913	G/A	Asthma	Caucasian	0.2331	3	277	534	0.891 (0.419-1.896)	0.765	85.9	0.001			
**Chronic obstructive pulmonary disease (COPD)**															
IL17A	rs2275913	G/A	COPD	Overall	0.2026	5	1404	1729	1.023 (0.778-1.344)	0.871	76.0	0.002	57.40	57.01	36.52
IL17A	rs2275913	G/A	COPD	Asian	0.4527	1	152	201	0.624 (0.455-0.875)	0.004	NA	NA			
IL17A	rs2275913	G/A	COPD	Mixed	0.1695	4	1252	1528	1.163 (0.976-1.385)	0.091	30.1	0.231			
IL17A	rs8193036	C/T	COPD	Overall	0.7273	5	1404	1729	1.070 (0.868-1.320)	0.525	65.1	0.022	63.06	57.04	36.52
IL17A	rs8193036	C/T	COPD	Asian	0.2985	1	152	201	1.448 (1.049-2.000)	0.025	NA	NA			
IL17A	rs8193036	C/T	COPD	Mixed	0.7833	4	1252	1528	0.995 (0.820-1.208)	0.960	52.5	0.097			
**Colorectal cancer (CRC)**															
IL17F	rs763780	T/C	Colorectal cancer	Overall	0.1880	6	1841	1307	1.267 (0.933-1.720)	0.130	58.9	0.033	66.74	68.71	45.60
IL17F	rs763780	T/C	Colorectal cancer	Asian	0.1417	3	1422	867	1.270 (0.825-1.956)	0.278	77.1	0.011			
IL17F	rs763780	T/C	Colorectal cancer	Caucasian	0.0754	2	302	340	1.403 (0.655-3.004)	0.384	65.8	0.087			
IL17F	rs763780	T/C	Colorectal cancer	Mixed	0.9700	1	117	100	1.003 (0.331-3.035)	0.996	NA	NA			
**Functional dyspepsia (FD)**															
IL17A	rs2275913	G/A	Functional dyspepsia	Asian	0.3936	3	175	564	0.815 (0.632-1.052)	0.116	0.0	0.943	14.87	11.74	8.80
IL17F	rs763780	T/C	Functional dyspepsia	Overall	0.1178	6	412	1314	0.891 (0.688-1.154)	0.383	0.0	0.667	15.55	21.22	14.12
IL17F	rs763780	T/C	Functional dyspepsia	Asian	0.1257	3	175	564	0.676 (0.447-1.022)	0.063	0.0	0.998			
IL17F	rs763780	T/C	Functional dyspepsia	Other	0.1120	3	237	750	1.089 (0.779-1.522)	0.617	0.0	0.952			
**Gastric cancer (GC)**															
IL17A	rs3748067	C/T	Gastric Cancer	Asian	0.3385	11	3022	5284	0.981 (0.750-1.283)	0.889	83.6	< 0.001	95.66	88.02	65.88
IL17A	rs4711998	A/G	Gastric Cancer	Asian	0.3316	3	1750	2066	1.001 (0.906-1.105)	0.989	41.7	0.180	79.64	66.51	43.77
IL17F	rs763780	T/C	Gastric Cancer	Asian	0.6781	10	3944	4566	1.042 (0.805-1.348)	0.756	87.4	< 0.001	98.15	94.76	77.23
**Hepatitis B Virus (HBV) infection**															
IL17A	rs2275913	G/A	Hepatitis B Virus (HBV) infection	Overall	0.4986	13	2633	2479	0.968 (0.834-1.124)	0.671	68.4	< 0.001	94.76	83.32	59.93
IL17A	rs2275913	G/A	Hepatitis B Virus (HBV) infection	Asian	0.5085	12	2434	2307	0.964 (0.821-1.133)	0.660	70.7	< 0.001			
IL17A	rs2275913	G/A	Hepatitis B Virus (HBV) infection	Caucasian	0.3663	1	199	172	1.024 (0.760-1.381)	0.875	NA	NA			
IL17A	rs4711998	A/G	Hepatitis B Virus (HBV) infection	Asian	0.2324	4	533	617	1.068 (0.678-1.683)	0.776	81.9	0.001	28.38	26.06	16.88
IL17A	rs8193036	C/T	Hepatitis B Virus (HBV) infection	Asian	0.2656	6	1260	945	0.947 (0.707-1.269)	0.717	74.7	0.001	60.46	52.59	33.38
**Hepatocellular carcinoma (HCC)**															
IL17A	rs2275913	G/A	Hepatocellular carcinoma (HCC)	Asian	0.5100	4	462	450	1.108 (0.755-1.626)	0.600	72.9	0.011	32.26	23.22	15.26
**Inflammatory Bowel Disease (IBD)**															
IL17A	rs2275913	G/A	Inflammatory Bowel Disease (IBD)	Asian	0.4202	3	580	764	1.051 (0.783-1.412)	0.740	71.1	0.031	38.56	27.93	17.96
IL17A	rs8193036	C/T	Inflammatory Bowel Disease (IBD)	Asian	0.2854	3	597	1016	1.012 (0.764-1.340)	0.936	65.1	0.057	34.62	28.60	18.35
**Lung cancer**															
IL17F	rs12203582	G/A	Lung cancer	Asian	0.5968	4	320	1432	1.103 (0.918-1.325)	0.293	0.0	0.790	22.94	17.52	12.04
IL17A	rs3819024	A/G	Lung cancer	Asian	0.4426	3	322	1098	0.940 (0.784-1.127)	0.503	0.0	0.832	23.93	17.60	12.08
**Periodontitis**															
IL17A	rs2275913	G/A	Periodontitis	Overall	0.3253	11	935	971	1.304 (0.892-1.908)	0.171	83.0	< 0.001	52.58	41.47	26.09
IL17A	rs2275913	G/A	Periodontitis	Caucasian	0.3547	3	535	524	0.942 (0.782-1.134)	0.526	0.0	0.744			
IL17A	rs2275913	G/A	Periodontitis	Mixed	0.2920	4	300	347	1.040 (0.514-2.103)	0.914	84.0	< 0.001			
IL17A	rs2275913	G/A	Periodontitis	Other	0.2800	4	100	100	2.498 (0.993-6.235)	0.052	74.3	0.009			
IL17F	rs763780	T/C	Periodontitis	Overall	0.0563	5	500	507	1.251 (0.840-1.860)	0.270	0.0	0.520	11.52	24.75	16.13
IL17F	rs763780	T/C	Periodontitis	Mixed	0.0656	4	300	347	1.506 (0.955-2.373)	0.078	0.0	0.935			
IL17F	rs763780	T/C	Periodontitis	Caucasian	0.0375	1	200	160	0.658 (0.281-1.543)	0.336	NA	NA			
**Pre-eclampsia (PE)**															
IL17A	rs2275913	G/A	Periodontitis	Overall	0.4129	3	1923	2296	1.009 (0.919-1.108)	0.848	0.0	0.860	85.82	70.59	47.21
IL17A	rs2275913	G/A	Periodontitis	Asian	0.4111	2	1662	2018	1.020 (0.922-1.129)	0.702	0.0	0.896			
IL17A	rs2275913	G/A	Periodontitis	Caucasian	0.4245	1	261	278	0.950 (0.745-1.210)	0.676	NA	NA			
IL17F	rs763780	T/C	Periodontitis	Overall	0.0979	3	1923	2296	0.995 (0.852-1.162)	0.947	54.4	0.111	46.52	70.59	47.21
IL17F	rs763780	T/C	Periodontitis	Asian	0.1017	2	1662	2018	0.938 (0.793-1.109)	0.451	0.0	0.342			
IL17F	rs763780	T/C	Periodontitis	Caucasian	0.0737	1	261	278	1.449 (0.948-2.217)	0.087	NA	NA			
**Recurrent Miscarriage (RM)**															
IL17A	rs2275913	G/A	Recurrent Miscarriage (RM)	Overall	0.4725	3	290	309	1.148 (0.753-1.752)	0.520	65.6	0.055	22.12	16.31	11.36
IL17A	rs2275913	G/A	Recurrent Miscarriage (RM)	Caucasian	0.5265	2	170	189	0.924 (0.666-1.282)	0.634	0.0	0.451			
IL17A	rs2275913	G/A	Recurrent Miscarriage (RM)	African	0.3875	1	120	120	1.634 (1.137-2.348)	0.008	NA	NA			
**Rheumatoid arthritis (RA)**															
IL17F	rs2397084	T/C	Rheumatoid arthritis (RA)	Caucasian	0.1056	4	800	695	1.575 (0.499-4.968)	0.439	94.9	< 0.001	23.90	36.48	23.01
IL17A	rs3819024	A/G	Rheumatoid arthritis (RA)	Overall	0.3712	3	2053	2266	0.914 (0.834-1.002)	0.056	0.0	0.603			
IL17A	rs3819024	A/G	Rheumatoid arthritis (RA)	Asian	0.4816	1	615	839	0.880 (0.758-1.021)	0.092	NA	NA			
IL17A	rs3819024	A/G	Rheumatoid arthritis (RA)	Caucasian	0.3842	1	937	928	0.913 (0.800-1.043)	0.179	NA	NA			
IL17A	rs3819024	A/G	Rheumatoid arthritis (RA)	Mixed	0.1513	1	501	499	1.019 (0.798-1.300)	0.882	NA	NA			
IL17F	rs763780	T/C	Rheumatoid arthritis (RA)	Overall	0.0713	9	1297	1264	1.650 (0.792-3.437)	0.181	89.9	< 0.001	26.62	57.36	34.91
IL17F	rs763780	T/C	Rheumatoid arthritis (RA)	Caucasian	0.0751	6	1039	980	2.070 (0.794-5.395)	0.137	92.9	< 0.001			
IL17F	rs763780	T/C	Rheumatoid arthritis (RA)	Mixed	0.0581	3	258	284	0.977 (0.527-1.812)	0.941	23.4	0.271			
IL17A	rs8193036	C/T	Rheumatoid arthritis (RA)	Overall	0.3941	3	2052	2261	1.074 (0.905-1.276)	0.414	65.8	0.054	87.56	73.37	49.70
IL17A	rs8193036	C/T	Rheumatoid arthritis (RA)	Asian	0.2555	1	615	839	1.250 (1.056-1.479)	0.010	NA	NA			
IL17A	rs8193036	C/T	Rheumatoid arthritis (RA)	Caucasian	0.2974	1	936	923	0.953 (0.827-1.097)	0.502	NA	NA			
IL17A	rs8193036	C/T	Rheumatoid arthritis (RA)	Mixed	0.7986	1	501	499	1.050 (0.843-1.309)	0.662	NA	NA			
**Tuberculosis (TB)**															
IL17A	rs3748067	C/T	Tuberculosis (TB)	Asian	0.1429	3	2365	2305	1.244 (0.939-1.649)	0.129	71.4	0.030	68.34	79.22	55.41
**Type 1 diabetes mellitus (T1DM)**															
IL17A	rs2275913	G/A	T1DM	Overall	0.3288	3	155	184	1.223 (0.887-1.685)	0.219	31.4	0.233	13.11	10.95	8.36
IL17A	rs2275913	G/A	T1DM	Other	0.2333	2	30	30	0.580 (0.229-1.466)	0.249	0.0	0.782			
IL17A	rs2275913	G/A	T1DM	Caucasian	0.3474	1	125	154	1.360 (0.965-1.918)	0.079	NA	NA			

Note: OR, odds ratio; A, adenine; T, thymine; G, guanine; C, cytosine; NA: Not Available.

[a]Major alleles/minor alleles.

[b]Frequency of minor allele in controls.

Supplementary Table 4: Associations between variants in the IL17 family genes associated with risk of human disease in meta-analysis under additive model based on two datasets.

ID	Gene	Variant	Alleles[a]	Disease	Ethnicity	Number Evaluation			Risk of Meta-Analysis		Heterogeneity		FPRP values[b]	Credibility of Evidence[c]
						Studies	Cases	Controls	OR (95%CI)	P_{value}	I^2(%)	P_Q		
**Ankylosing spondylitis (AS)**														
1	IL17A	rs2275913	G/A	Ankylosing spondylitis (AS)	Caucasian	2	810	901	1.028 (0.868-1.217)	0.748	9.9	0.292		
**2**	**IL17RA**	**rs4819554**	**G/A**	**Ankylosing spondylitis (AS)**	**Caucasian**	**2**	**599**	**956**	**0.776 (0.652-0.923)**	**0.004**	**0.0**	**0.697**	0.076	Moderate
**Asthma**														
3	IL17A	rs3748067	C/T	Asthma	Asian	2	702	769	1.705 (0.721-4.0.5)	0.225	87.4	0.005		
**Autoimmune thyroid diseases (AITD)**														
4	IL17A	rs2275913	G/A	Autoimmune thyroid diseases (AITD)	Asian	2	508	448	0.992 (0.825-1.194)	0.934	0.0	0.865		
5	IL17A	rs3819025	G/A	Autoimmune thyroid diseases (AITD)	Asian	2	508	448	0.796 (0.568-1.115)	0.185	55.5	0.134		
6	IL17A	rs8193037	G/A	Autoimmune thyroid diseases (AITD)	Asian	2	508	448	0.983 (0.736-1.312)	0.906	0.0	0.869		
**Behcet's disease (BD)**														
**7**	**IL17A**	**rs2275913**	**G/A**	**Behcet's disease (BD)**	**Asian**	**2**	**503**	**671**	**0.844 (0.715-0.997)**	**0.046**	**0.0**	**0.878**	0.467	Weak
8	IL17F	rs763780	T/C	Behcet's disease (BD)	Asian	2	461	526	0.969 (0.748-1.254)	0.811	0.0	0.933		
**Bladder Cancer**														
9	IL17A	rs2275913	G/A	Bladder Cancer	Overall	2	476	653	1.204 (0.899-1.614)	0.213	62.4	0.103		
**10**	**IL17F**	**rs763780**	**T/C**	**Bladder Cancer**	**Overall**	**2**	**476**	**653**	**0.697 (0.522-0.930)**	**0.014**	**0.0**	**0.793**	0.303	Weak
**Bronchiolitis**														
**11**	**IL17A**	**rs2275913**	**G/A**	**Bronchiolitis**	**Overall**	**2**	**309**	**610**	**1.223 (1.005-1.488)**	**0.045**	**0.0**	**0.342**	0.462	Weak
**Brucellosis**														
12	IL17A	rs1974226	C/T	Brucellosis	Caucasian	2	262	170	1.584 (0.355-7.054)	0.546	91.3	0.001		
13	IL17A	rs2275913	G/A	Brucellosis	Caucasian	2	262	170	0.303 (0.065-1.420)	0.130	94.0	< 0.001		
14	IL17A	rs3748067	C/T	Brucellosis	Caucasian	2	262	170	0.436 (0.010-18.486)	0.664	98.6	< 0.001		
15	IL17A	rs3804513	A/T	Brucellosis	Caucasian	2	262	170	0.900 (0.471-1.721)	0.750	70.8	0.064		
**16**	**IL17A**	**rs3819024**	**A/G**	**Brucellosis**	**Caucasian**	**2**	**262**	**170**	**0.371 (0.156-0.883)**	**0.025**	**88.1**	**0.004**	0.837	Weak
17	IL17A	rs3819025	G/A	Brucellosis	Caucasian	2	262	170	0.156 (0.017-1.413)	0.098	95.5	< 0.001		
18	IL17A	rs4711998	A/G	Brucellosis	Caucasian	2	262	170	3.306 (0.143-76.536)	0.456	98.9	< 0.001		
19	IL17A	rs8193036	C/T	Brucellosis	Caucasian	2	262	170	0.854 (0.553-1.319)	0.476	50.3	0.156		
20	IL17A	rs8193038	A/G	Brucellosis	Caucasian	2	262	170	0.369 (0.094-1.445)	0.152	80.0	0.025		
**Cervical cancer**														
21	IL17F	rs1266828	G/A	Cervical cancer	Asian	2	480	696	0.884 (0.752-1.054)	0.170	0.0	0.688		
22	IL17A	rs3819025	G/A	Cervical cancer	Asian	2	480	696	0.939 (0.777-1.136)	0.519	0.0	0.650		
23	IL17F	rs9382084	G/T	Cervical cancer	Asian	2	480	696	1.157 (0.976-1.370)	0.290	0.0	0.980		
**Chagas disease (CD)**														
**24**	**IL17A**	**rs2275913**	**G/A**	**Chronic Chagas cardiomyopathy (CCC)**	**Mixed**	**2**	**260**	**300**	**1.421 (1.063-1.900)**	**0.018**	**0.0**	**0.474**	0.344	Weak
25	IL17F	rs763780	T/C	Chronic Chagas cardiomyopathy (CCC)	Mixed	2	260	300	1.149 (0.691-1.910)	0.592	0.0	0.818		
**Colorectal cance (CRC)**														
**26**	**IL17**	**rs6973569**	**G/A**	**Colorectal cance (CRC)**	**Asian**	**2**	**601**	**1254**	**0.796 (0.655-0.967)**	**0.022**	**0.0**	**0.334**	0.298	Weak
**Coronary artery disease (CAD)**														
27	IL17A	rs3819024	A/G	Coronary artery disease	Asian	2	1250	1154	0.927 (0.757-1.135)	0.461	51.4	0.152		
28	IL17A	rs3819025	G/A	Coronary artery disease (CAD)	Asian	2	1446	1383	0.904 (0.801-1.021)	0.102	0.0	0.704		
29	IL17F	rs763780	T/C	Coronary artery disease	Asian	2	497	434	1.245 (0.933-1.662)	0.136	0.0	0.579		
**30**	**IL17A**	**rs8193036**	**C/T**	**Coronary artery disease (CAD)**	**Asian**	**2**	**410**	**357**	**0.675 (0.519-0.878)**	**0.003**	**0.0**	**0.334**	0.107	Moderate
**31**	**IL17A**	**rs8193037**	**G/A**	**Coronary artery disease (CAD)**	**Asian**	**2**	**1250**	**1154**	**0.653 (0.534-0.798)**	**< 0.001**	**0.0**	**0.461**	0.001	Strong
**Enterovirus 71 infection**														
32	IL17F	rs1889570	C/T	Enterovirus 71 infection	Asian	2	336	660	1.326 (0.843-3.085)	0.222	81.0	0.022		
33	IL17F	rs4715290	A/G	Enterovirus 71 infection	Asian	2	336	660	0.922 (0.696-1.222)	0.572	0.0	0.525		
**Gastric cancer**														
34	IL17A	rs3819024	A/G	Gastric cancer	Asian	2	1364	1692	0.952 (0.860-1.055)	0.349	0.0	0.560		
35	IL17A	rs3819025	G/A	Gastric cancer	Asian	2	536	1026	0.843 (0.641-1.108)	0.220	45.0	0.178		
36	IL17A	rs8193036	C/T	Gastric cancer	Asian	2	1364	1692	1.102 (0.912-1.142)	0.728	0.0	0.718		
37	IL17F	rs9382084	G/T	Gastric cancer	Asian	2	293	1100	1.036 (0.861-1.247)	0.705	0.0	0.802		
**Gastric cancer**														
38	IL17A	rs3819024	A/G	Gastric cancer	Asian	2	1364	1692	0.952 (0.860-1.055)	0.349	0.0	0.560		
39	IL17A	rs3819025	G/A	Gastric cancer	Asian	2	536	1026	0.843 (0.641-1.108)	0.220	45.0	0.178		
40	IL17A	rs8193036	C/T	Gastric cancer	Asian	2	1364	1692	1.020 (0.912-1.142)	0.728	0.0	0.718		
41	IL17F	rs9382084	G/T	Gastric cancer	Asian	2	293	1100	1.036 (0.861-1.247)	0.705	0.0	0.802		
**Gastro-duodenal ulcer (UL)**														
**42**	**IL17A**	**rs2275913**	**G/A**	**Gastro-duodenal ulcer (UL)**	**Asian**	**2**	**185**	**726**	**1.416 (1.123-1.785)**	**0.003**	**0.0**	**0.631**	0.082	Moderate
43	IL17F	rs763780	T/C	Gastro-duodenal ulcer (UL)	Asian	2	185	726	0.647 (0.335-1.252)	0.197	49.4	0.160		
**Hepatocellular carcinoma (HCC)**														
**44**	**IL17A**	**rs4711998**	**A/G**	**Hepatocellular carcinoma (HCC)**	**Asian**	**2**	**1041**	**1257**	**0.870 (0.759-0.997)**	**0.045**	**0.0**	**0.636**	0.462	Weak
45	IL17A	rs8193036	C/T	Hepatocellular carcinoma (HCC)	Asian	2	272	259	1.137 (0.860-1.504)	0.367	0.0	0.422		
**Inflammatory Bowel Disease (IBD)**														
46	IL17A	rs3819025	G/A	Inflammatory Bowel Disease (IBD)	Asian	2	469	516	1.115 (0.884-1.406)	0.357	0.0	0.534		
**Lung cancer (LC)**														
47	IL17F	rs763780	T/C	Lung cancer (LC)	Overall	2	559	616	1.333 (0.798-2.225)	0.272	64.5	0.095		
**Multiple sclerosis (MS)**														
48	IL17A	rs2275913	G/A	Multiple sclerosis (MS)	Asian	2	691	874	1.014 (0.880-1.168)	0.851	0.0	0.594		
**Osteoarthritis (OA)**														
49	IL17F	rs1889570	C/T	Osteoarthritis (OA)	Caucasian	2	474	372	0.964 (0.796-1.168)	0.709	0.0	0.425		
**Papillary thyroid cancer (PTC)**														
50	IL17A	rs2275913	G/A	Papillary thyroid cancer (PTC)	Overall	2	282	430	1.120 (0.886-1.417)	0.344	0.0	0.834		
**51**	**IL17RA**	**rs4819554**	**G/A**	**Papillary thyroid cancer (PTC)**	**Overall**	**2**	**281**	**428**	**1.293 (1.013-1.650)**	**0.039**	**0.0**	**0.502**	0.455	Weak
**Periodontitis**														
52	IL-17R	rs879576	G/A	Periodontitis	Caucasian	2	110	166	0.868 (0.544-1.385)	0.553	0.0	0.858		
**Pre-eclampsia (PE)**														
53	IL17RA	rs4819554	G/A	Pre-eclampsia (PE)	Asian	2	1662	2018	0.997 (0.902-1.013)	0.961	0.0	0.449		
**Psoriasis**														
54	IL17RA	rs4819554	G/A	Psoriasis	Overall	2	360	347	1.254 (0.668-2.353)	0.482	86.9	0.006		
55	IL17A	rs2275913	G/A	Psoriasis	Caucasian	2	601	402	0.963 (0.795-1.166)	0.696	0.0	0.521		
56	IL17F	rs2397084	T/C	Psoriasis	Caucasian	2	987	772	0.846 (0.661-1.081)	0.181	0.0	0.992		
57	IL17RA	rs4819554	G/A	Psoriasis	Overall	2	680	667	0.566 (0.291-1.101)	0.094	88.3	0.004		
**Recurrent Miscarriage (RM)**														
**58**	**IL17F**	**rs763780**	**T/C**	**Recurrent Miscarriage**	**Overall**	**2**	**205**	**205**	**0.641 (0.483-0.851)**	**0.002**	**0.0**	**0.444**	0.092	Moderate
**Rheumatoid arthritis (RA)**														
59	IL17A	rs4711998	A/G	Rheumatoid arthritis (RA)	Overall	2	1529	1738	1.069 (0.957-1.195)	0.238	0.0	0.888		
60	IL17A	rs8193037	G/A	Rheumatoid arthritis (RA)	Overall	2	1116	1338	1.050 (0.809-1.364)	0.712	44.8	0.178		
**Silicosis**														
**61**	**IL17F**	**rs763780**	**T/C**	**Silicosis**	**Overall**	**2**	**161**	**178**	**0.511 (0.266-0.980)**	**0.043**	**15.7**	**0.276**	0.795	Weak
**Systemic lupus erythematosus (SLE)**														
62	IL17F	rs2397084	T/C	Systemic lupus erythematosus (SLE)	Overall	2	249	401	0.804 (0.549-1.179)	0.264	0.0	0.896		
63	IL17F	rs763780	T/C	Systemic lupus erythematosus (SLE)	Overall	2	249	401	2.123 (0.894-5.043)	0.088	72.5	0.056		
64	IL17A	rs8193036	C/T	Systemic lupus erythematosus (SLE)	Overall	2	684	819	0.967 (0.824-1.136)	0.685	0.0	0.498		
**Tuberculosis (TB)**														
**65**	**IL17F**	**rs1889570**	**C/T**	**Tuberculosis (TB)**	**Asian**	**2**	**772**	**1244**	**0.859 (0.751-0.982)**	**0.026**	**0.0**	**0.560**	0.331	Weak
66	IL17A	rs8193036	C/T	Tuberculosis (TB)	Asian	2	2524	2474	0.917 (0.724-1.163)	0.476	86.2	0.007		

Note: OR, odds ratio; A, adenine; T, thymine; G, guanine; C, cytosine; NA: Not Available.

[a]**Major alleles/minor alleles.**

[b]The prior probability of FPRP is 0.05, and the FPRP level of noteworthiness is 0.20.

[c]Degree of epidemiological credibility based on the combination of results from Venice guidelines and FPRP tests (Strong, if FPRP < 0.05; Moderate, if 0.05 ≤ FPRP ≤ 0.2; Weak, if FPRP > 0.2).

Supplementary Table 5: Associations between variants in the IL17 family genes associated with risk of human disease in meta-analysis under additive model based on one dataset.

PMID/Ref	First author	Publishing Year	Disease	Country/region	Ethnicity	Study design	Case	Control	Sample size	Gene name	Variant	Allelic[a]	OR 95%CI	FPRP[b]
16630936	Kawaguchi, M	2006	Asthma	Japan	Asian	Case-control study	432	435	867	IL17F	rs1266828 G/A	G/A	1.034 (0.820-1.303)	
16630936	Kawaguchi, M	2006	Asthma	Japan	Asian	Case-control study	432	435	867	IL17F	rs7771511 C/T	C/T	1.137 (0.838-1.542)	
16630936	Kawaguchi, M	2006	Asthma	Japan	Asian	Case-control study	432	435	867	IL17F	rs766748 C/T	C/T	1.196 (0.960-1.490)	
18774769	Metzger, K	2008	Chronic fatigue syndrome (CFS)	Belgium	Caucasian	Case-control study	89	56	145	IL17F	rs763780 T/C	T/C	**0.246 (0.103-0.587)**	0.708
19019635	Shibata, S	2009	Psoriasis vulgaris (PsV)	Japan	Asian	Case-control study	153	103	256	IL17F	rs763780 T/C	T/C	1.212 (0.687-2.137)	
19019635	Shibata, S	2009	Atopic dermatitis (AD)	Japan	Asian	Case-control study	160	103	263	IL17F	rs763780 T/C	T/C	1.442 (0.831-2.500)	
19118269	Jung, J. S	2009	Asthma	Korea	Asian	Case-control study	954	265	1219	IL17RB	rs28385732 G/A	G/A	1.091 (0.799-1.490)	
19118269	Jung, J. S	2009	Asthma	Korea	Asian	Case-control study	954	265	1219	IL17RB	rs3733072 G/A	G/A	**0.619 (0.474-0.807)**	0.025
19118269	Jung, J. S	2009	Asthma	Korea	Asian	Case-control study	954	265	1219	IL17RB	rs3733075 C/T	C/T	0.896 (0.723-1.110)	
19118269	Jung, J. S	2009	Asthma	Korea	Asian	Case-control study	954	265	1219	IL17RB	rs6766099 C/T	C/T	1.002 (0.803-1.252)	
19118269	Jung, J. S	2009	Asthma	Korea	Asian	Case-control study	954	265	1219	IL17RB	rs1043261 C/T	C/T	1.111 (0.804-1.534)	
19118269	Jung, J. S	2009	Asthma	Korea	Asian	Case-control study	954	265	1219	IL17RB	rs3017 A/G	A/G	1.014 (0.830-1.237)	
19208686	Nordang, G. B	2009	Rheumatoid arthritis (RA)	Norway	Caucasian	Case-control study	921	920	1841	IL17A	rs7747909 G/A	G/A	0.980 (0.837-1.146)	
19210369	Wang, J. Y	2009	Asthma	China (Taipei)	Asian	Case-control study	481	546	1027	IL17A	rs4711998 A/G	A/G	0.922 (0.754-1.128)	
19210369	Wang, J. Y	2009	Asthma	China (Taipei)	Asian	Case-control study	481	546	1027	IL17A	rs3819024 A/G	A/G	1.085 (0.909-1.296)	
19210369	Wang, J. Y	2009	Asthma	China (Taipei)	Asian	Case-control study	481	546	1027	IL17A	rs3819025 G/A	G/A	1.020 (0.810-1.284)	
19210369	Wang, J. Y	2009	Asthma	China (Taipei)	Asian	Case-control study	481	546	1027	IL17A	rs8193038 A/G	A/G	1.099 (0.829-1.457)	
19210369	Wang, J. Y	2009	Asthma	China (Taipei)	Asian	Case-control study	481	546	1027	IL17A	rs3804513 A/T	A/T	**0.244 (0.157-0.378)**	0.002
19210369	Wang, J. Y	2009	Asthma	China (Taipei)	Asian	Case-control study	481	546	1027	IL17A	rs1974226 C/T	C/T	**0.168 (0.115-0.245)**	0.000
19838108	Pei, F	2009	Myocardial infarction (MI)	China	Asian	Case-control study	513	477	990	IL17F	rs763780 T/C	T/C	0.922 (0.695-1.223)	
20620187	Shu, Q	2010	Vogt–Koyanagi–Harada (VKH) syndrome	China	Asian	Case-control study	385	412	797	IL17F	rs763780 T/C	T/C	**0.657 (0.487-0.886)**	0.195
20620187	Shu, Q	2010	Vogt–Koyanagi–Harada (VKH) syndrome	China	Asian	Case-control study	385	412	797	IL17A	rs2275913 G/A	G/A	1.151 (0.945-1.402)	
21062626	Zhang, X	2010	Coronary artery disease (CAD)	China	Asian	Case-control study	1031	935	1966	IL17A	rs4711998 A/G	A/G	1.096 (0.941-1.277)	
21737283	Jiang, N	2011	Brain arteriovenous malformation (BAVM)	China	Asian	Case-control study	53	120	173	IL17A	rs2275913 G/A	G/A	0.645 (0.407-1.024)	
22118860	Wang, H	2012	Neuromyelitisoptica (NMO)	China	Asian	Case-control study	52	131	183	IL17A	rs2275913 G/A	G/A	1.046 (0.663-1.650)	
22118860	Wang, H	2012	Neuromyelitisoptica (NMO)	China	Asian	Case-control study	52	131	183	IL17F	rs763780 T/C	T/C	**0.330 (0.136-0.803)**	0.802
22123380	Kohyama, K	2011	Aspirin-exacerbated respiratory disease (AERD)	Japan	Asian	Case-control study	95	100	195	IL17A	rs8193036 C/T	C/T	0.688 (0.448-1.056)	
22123380	Kohyama, K	2011	Aspirin tolerant asthma (ATA)	Japan	Asian	Case-control study	300	100	400	IL17A	rs8193036 C/T	C/T	1.113 (0.798-1.552)	
22461912	Wang, L	2012	Breast cancer	China	Asian	Case-control study	491	502	993	IL17A	rs2275913 G/A	G/A	**1.316 (1.098-1.577)**	0.057
22461912	Wang, L	2012	Breast cancer	China	Asian	Case-control study	491	502	993	IL17A	rs3819025 G/A	G/A	**0.521 (0.408-0.665)**	0.000
22461912	Wang, L	2012	Breast cancer	China	Asian	Case-control study	491	502	993	IL17A	rs3748067 C/T	C/T	0.923 (0.736-1.158)	
22461912	Wang, L	2012	Breast cancer	China	Asian	Case-control study	491	502	993	IL17F	rs763780 T/C	T/C	1.046 (0.794-1.378)	
22461912	Wang, L	2012	Breast cancer	China	Asian	Case-control study	491	502	993	IL17F	rs7771511 C/T	C/T	1.025 (0.776-1.354)	
22461912	Wang, L	2012	Breast cancer	China	Asian	Case-control study	491	502	993	IL17F	rs12203582 G/A	G/A	1.112 (0.925-1.337)	
22461912	Wang, L	2012	Breast cancer	China	Asian	Case-control study	491	502	993	IL17F	rs9382084 G/T	G/T	1.098 (0.913-1.319)	
22461912	Wang, L	2012	Breast cancer	China	Asian	Case-control study	491	502	993	IL17F	rs1266828 G/A	G/A	0.977 (0.773-1.235)	
22483685	Kim, E. S	2012	Behcet's disease (BD)	Korea	Asian	Case-control study	141	259	400	IL17A	rs4711998 A/G	A/G	1.376 (0.999-1.896)	
22483685	Kim, E. S	2012	Behcet's disease (BD)	Korea	Asian	Case-control study	141	259	400	IL17A	rs8193036 C/T	C/T	1.311 (0.963-1.784)	
22483685	Kim, E. S	2012	Behcet's disease (BD)	Korea	Asian	Case-control study	141	259	400	IL17A	rs9791323 A/C	A/C	1.427 (0.804-2.534)	
22483685	Kim, E. S	2012	Behcet's disease (BD)	Korea	Asian	Case-control study	141	259	400	IL17A	rs3819024 A/G	A/G	0.896 (0.669-1.200)	
22483685	Kim, E. S	2012	Behcet's disease (BD)	Korea	Asian	Case-control study	141	259	400	IL17A	rs8193037 G/A	G/A	1.017 (0.661-1.564)	
22507625	Wang, M	2012	Allergic rhinitis (AR)	China	Asian	Case-control study	279	281	560	IL17A	rs4711998 A/G	A/G	0.901 (0.683-1.189)	
22507625	Wang, M	2012	Allergic rhinitis (AR)	China	Asian	Case-control study	279	281	560	IL17A	rs8193036 C/T	C/T	0.936 (0.718-1.221)	
22507625	Wang, M	2012	Allergic rhinitis (AR)	China	Asian	Case-control study	279	281	560	IL17A	rs3819024 A/G	A/G	**4.868 (3.939-6.017)**	0.000
22507625	Wang, M	2012	Allergic rhinitis (AR)	China	Asian	Case-control study	279	281	560	IL17A	rs3819025 G/A	G/A	0.887 (0.643-1.222)	
22507625	Wang, M	2012	Allergic rhinitis (AR)	China	Asian	Case-control study	279	281	560	IL17A	rs3804513 A/T	A/T	1.314 (0.823-1.870)	
22507625	Wang, M	2012	Allergic rhinitis (AR)	China	Asian	Case-control study	279	281	560	IL17A	rs1974226 C/T	C/T	0.997 (0.784-1.269)	
22507625	Wang, M	2012	Allergic rhinitis (AR)	China	Asian	Case-control study	279	281	560	IL17F	rs13209590 A/G	A/G	1.172 (0.838-1.638)	
22507625	Wang, M	2012	Allergic rhinitis (AR)	China	Asian	Case-control study	279	281	560	IL17F	rs7771466 C/A	C/A	**0.672 (0.459-0.985)**	0.605
22507625	Wang, M	2012	Allergic rhinitis (AR)	China	Asian	Case-control study	279	281	560	IL17F	rs1266828 G/A	G/A	1.202 (0.864-1.673)	
22507625	Wang, M	2012	Allergic rhinitis (AR)	China	Asian	Case-control study	279	281	560	IL17F	rs669161 A/G	A/G	0.994 (0.780-1.267)	
22507625	Wang, M	2012	Allergic rhinitis (AR)	China	Asian	Case-control study	197	281	478	IL17A	rs4711998 A/G	A/G	1.220 (0.910-1.635)	
22507625	Wang, M	2012	Allergic rhinitis (AR)	China	Asian	Case-control study	197	281	478	IL17A	rs8193036 C/T	C/T	1.210 (0.910-1.610)	
22507625	Wang, M	2012	Allergic rhinitis (AR)	China	Asian	Case-control study	197	281	478	IL17A	rs3819024 A/G	A/G	0.836 (0.640-1.093)	
22507625	Wang, M	2012	Allergic rhinitis (AR)	China	Asian	Case-control study	197	281	478	IL17A	rs3819025 G/A	G/A	0.909 (0.639-1.292)	
22507625	Wang, M	2012	Allergic rhinitis (AR)	China	Asian	Case-control study	197	281	478	IL17A	rs1974226 C/T	C/T	1.073 (0.823-1.399)	
22507625	Wang, M	2012	Allergic rhinitis (AR)	China	Asian	Case-control study	197	281	478	IL17F	rs13209590 A/G	A/G	0.711 (0.473-1.069)	
22507625	Wang, M	2012	Allergic rhinitis (AR)	China	Asian	Case-control study	197	281	478	IL17F	rs7771466 C/A	C/A	1.111 (0.724-1.706)	
22507625	Wang, M	2012	Allergic rhinitis (AR)	China	Asian	Case-control study	197	281	478	IL17F	rs1266828 G/A	G/A	1.078 (0.755-1.538)	
22507625	Wang, M	2012	Allergic rhinitis (AR)	China	Asian	Case-control study	197	281	478	IL17F	rs669161 A/G	A/G	0.790 (0.605-1.031)	
22559912	Popovic K, D	2013	Primary antiphospholipid syndrome (PAPS)	Serbia	Caucasian	Case-control study	50	50	100	IL17A	rs2275913 G/A	G/A	1.088 (0.616-1.920)	
22812194	Qian, F	2012	Asthma	China	Asian	Case-control study	318	352	670	IL17F	rs13209590 A/G	A/G	0.957 (0.704-1.301)	
22999050	Zhou, S	2012	Adolescent idiopathic scoliosis (AIS)	China	Asian	Case-control study	529	512	1041	IL-17RC	rs708567 C/T	C/T	**0.664 (0.460-0.956)**	0.517
23111159	Guo, T	2013	Autoimmune thyroid diseases (AITD)	China	Asian	Case-control study	757	741	1498	IL17F	rs761167 C/T	C/T	0.927 (0.797-1.079)	
23111159	Guo, T	2013	Autoimmune thyroid diseases (AITD)	China	Asian	Case-control study	757	741	1498	IL17F	rs9463772 C/T	C/T	**1.535 (1.241-1.900)**	0.004
23111159	Guo, T	2013	Autoimmune thyroid diseases (AITD)	China	Asian	Case-control study	757	741	1498	IL17F	rs2397084 T/C	T/C	1.101 (0.548-2.213)	
23280722	Li, N	2014	Chronic hepatitis (CH)	China	Asian	Case-control study	134	125	259	IL17A	rs8193036 C/T	C/T	0.795 (0.530-1.193)	
23280722	Li, N	2014	Chronic hepatitis (CH)	China	Asian	Case-control study	134	125	259	IL17A	rs2275913 G/A	G/A	**1.981 (1.398-2.807)**	0.038
23323524	adkhodazadeh ,	2013	Periodontitis	Iran	Caucasian	Cross-Sectional Study	75	84	159	IL17A	rs10484879 C/A	C/A	0.822 (0.477-1.417)	
23323524	adkhodazadeh ,	2013	peri-implantitis (PI)	Iran	Caucasian	Cross-Sectional Study	38	84	122	IL17A	rs10484879 C/A	C/A	**0.445 (0.203-0.976)**	0.840
23376081	Peng, Y	2013	Dilated cardiomyopathy (DCM)	China	Asian	Case-control study	288	421	709	IL17A	rs2275913 G/A	G/A	1.075 (0.867-1.333)	
23376081	Peng, Y	2013	Dilated cardiomyopathy (DCM)	China	Asian	Case-control study	288	421	709	IL17F	rs763780 T/C	T/C	1.033 (0.770-1.385)	
23485736	Nie, K	2013	Parkinson's Disease	China	Asian	Case-control study	302	294	596	IL17A	rs8193036 C/T	C/T	1.211 (0.925-1.587)	
23485736	Nie, K	2013	Parkinson's Disease	China	Asian	Case-control study	302	294	596	IL17A	rs2275913 G/A	G/A	1.119 (0.891-1.405)	
23895419	Yin, J	2013	Esophageal cancer (EC)	China	Asian	Case-control study	380	380	760	IL17A	rs2275913 G/A	G/A	1.077 (0.877-1.322)	
23895419	Yin, J	2013	Esophageal cancer (EC)	China	Asian	Case-control study	380	380	760	IL17A	rs3819024 A/G	A/G	1.138 (0.928-1.397)	
23895419	Yin, J	2013	Esophageal cancer (EC)	China	Asian	Case-control study	380	380	760	IL17A	rs3819025 G/A	G/A	0.883 (0.665-1.170)	
23895419	Yin, J	2013	Esophageal cancer (EC)	China	Asian	Case-control study	380	380	760	IL17A	rs4711998 A/G	A/G	0.859 (0.681-1.082)	
23895419	Yin, J	2013	Esophageal cancer (EC)	China	Asian	Case-control study	380	380	760	IL17A	rs8193036 C/T	C/T	0.959 (0.764-1.203)	
23895419	Yin, J	2013	Esophageal cancer (EC)	China	Asian	Case-control study	380	380	760	IL17A	rs8193037 G/A	G/A	0.873 (0.627-1.215)	
24315816	Qinghai, Z	2014	Gastric Cancer (GC)	China	Asian	Case-control study	293	550	843	IL17F	rs12203582 G/A	G/A	0.864 (0.704-1.059)	
24548421	Kaabachi, W	2014	Lung cancer (LC)	Tunisia	Caucasian	Case-control study	239	258	497	IL17F	rs2397084 T/C	T/C	0.770 (0.479-1.236)	
24690400	arakas Celik,	2014	Subacute sclerosing panencephalitis (SSPE)	Turkey	Caucasian	Case-control study	54	81	135	IL17F	rs763780 T/C	T/C	**0.114 (0.026-0.494)**	0.885
24690400	arakas Celik,	2014	Subacute sclerosing panencephalitis (SSPE)	Turkey	Caucasian	Case-control study	54	81	135	IL17F	rs2397084 T/C	T/C	1.460 (0.674-3.162)	
24690400	arakas Celik,	2014	Subacute sclerosing panencephalitis (SSPE)	Turkey	Caucasian	Case-control study	54	81	135	IL17A	rs2275913 G/A	G/A	1.127 (0.680-1.868)	
24793548	Wróbel, T	2014	Leukemia	Poland	Caucasian	Case-control study	62	125	187	IL17A	rs2275913 G/A	G/A	0.998 (0.646-1.542)	
24793548	Wróbel, T	2014	Leukemia	Poland	Caucasian	Case-control study	62	125	187	IL17F	rs763780 T/C	T/C	**5.487 (2.600-11.579)**	0.311
24893702	Ren, Z	2014	Gastric Cancer (GC)	China	Asian	Case-control study	243	476	719	IL17A	rs8193037 G/A	G/A	1.031 (0.735-1.446)	
25159155	aradowska G,	2015	Mixed connective tissue disease (MCTD)	Poland	Caucasian	Case-control study	66	106	172	IL17F	rs763780 T/C	T/C	**3.269 (1.346-7.942)**	0.799
25223230	Narbutt, J	2015	Atopic dermatitis (AD)	Poland	Caucasian	Case-control study	166	160	326	IL17A	rs2275913 G/A	G/A	1.260 (0.905-1.754)	
25296730	Ma, M	2015	Colorectal cance (CRC)	China	Asian	Case-control study	1000	354	1354	IL17F	rs2397084 T/C	T/C	1.021 (0.802-1.300)	
25374205	Chen, Y	2014	Silicosis	China	Asian	Case-control study	113	116	229	IL17A	A-832G A/G	A/G	1.060 (0.710-1.581)	
25429834	Xi, X. E	2015	Hepatocellular carcinoma (HCC)	China	Asian	Case-control study	155	171	326	IL17F	rs763780 T/C	T/C	1.029 (0.703-1.507)	
25469655	Ma, Q. Y	2015	Lung cancer (LC)	China	Asian	Case-control study	330	382	712	IL17A	rs142078089 A/G	A/G	0.940 (0.762-1.160)	
25469655	Ma, Q. Y	2015	Lung cancer (LC)	China	Asian	Case-control study	330	382	712	IL17A	rs17879568 G/A	G/A	**1.993 (1.508-2.633)**	0.010
25484349	Lee, Y. C	2015	Papillary thyroid cancer (PTC)	Korea	Asian	Case-control study	94	260	354	IL17A	rs3819024 A/G	A/G	1.123 (0.804-1.569)	
25484349	Lee, Y. C	2015	Papillary thyroid cancer (PTC)	Korea	Asian	Case-control study	94	260	354	IL17RA	rs879575 C/T	C/T	0.827 (0.225-3.038)	
25484349	Lee, Y. C	2015	Papillary thyroid cancer (PTC)	Korea	Asian	Case-control study	94	260	354	IL17RA	rs879577 C/T	C/T	0.546 (0.224-1.334)	
25484349	Lee, Y. C	2015	Papillary thyroid cancer (PTC)	Korea	Asian	Case-control study	94	260	354	IL17RA	rs2229151 G/A	G/A	1.168 (0.831-1.643)	
25484349	Lee, Y. C	2015	Papillary thyroid cancer (PTC)	Korea	Asian	Case-control study	94	260	354	IL17RB	rs2232350 T/C	T/C	0.655 (0.413-1.038)	
25484349	Lee, Y. C	2015	Papillary thyroid cancer (PTC)	Korea	Asian	Case-control study	94	260	354	IL17RB	rs1025689 C/G	C/G	0.760 (0.542-1.065)	
25510225	Boroń, D	2014	Osteoporosis	Poland	Caucasian	Case-control study	306	158	464	IL17A	rs2275913 G/A	G/A	0.867 (0.657-1.144)	
25579009	Li, N	2015	Oral squamous cell carcinoma (OSCC)	China	Asian	Case-control study	121	103	224	IL17A	rs2275913 G/A	G/A	**1.736 (1.168-2.581)**	0.341
25579009	Li, N	2015	Oral squamous cell carcinoma (OSCC)	China	Asian	Case-control study	121	101	222	IL17F	rs763780 T/C	T/C	1.468 (0.804-2.678)	
25579009	Li, N	2015	Oral squamous cell carcinoma (OSCC)	China	Asian	Case-control study	121	101	222	IL17F	rs9382084 G/T	G/T	**1.501 (1.026-2.196)**	0.581

25636567	Coto , E	2015	Chronic kidney disease (CKD)	Spain	Caucasian	Case-control study	90	560	650	IL17RA	rs4819554 G/A	G/A	**1.862 (1.175-2.950)**	0.463
25871515	Shen, L	2015	Rheumatoid arthritis (RA)	China	Asian	Case-control study	615	839	1454	IL17A	rs3819025 G/A	G/A	**1.233 (1.011-1.504)**	0.431
26017027	Aytekin , N	2015	Aopecia areata	Turkey	Caucasian	Case-control study	100	71	171	IL17F	rs763780 T/C	T/C	1.619 (0.787-3.328)	
26045845	Du, J	2015	Tuberculosis (TB)	China	Asian	Case-control study	428	428	856	IL17F	rs9382084 G/T	G/T	1.109 (0.914-1.344)	
26073462	He, Y	2017	Lung cancer (LC)	China	Asian	Case-control study	320	358	678	IL17A	rs3748067 C/T	C/T	0.758 (0.563-1.022)	
26073462	He, Y	2017	Lung cancer (LC)	China	Asian	Case-control study	320	358	678	IL17A	rs3819025 G/A	G/A	0.836 (0.623-1.122)	
26073462	He, Y	2017	Lung cancer (LC)	China	Asian	Case-control study	320	358	678	IL17F	rs1266828 G/A	G/A	0.756 (0.554-1.031)	
25615631	argas Alarcón,	2015	Coronary artery disease (CAD)	Mexico	Mixed	Case-control study	900	667	1567	IL17A	rs8193036 C/T	C/T	0.982 (0.827-1.166)	
25615631	argas Alarcón,	2015	Coronary artery disease (CAD)	Mexico	Mixed	Case-control study	900	667	1567	IL17A	rs3819024 A/G	A/G	0.864 (0.719-1.038)	
25615631	argas Alarcón,	2015	Coronary artery disease (CAD)	Mexico	Mixed	Case-control study	900	667	1567	IL17A	rs8193037 G/A	G/A	1.137 (0.868-1.488)	
26223249	Han , R	2015	Coal workers ' pneumoconiosis (CWP)	China	Asian	Case-control study	692	687	1379	IL17A	rs2275913 G/A	G/A	1.020 (0.877-1.185)	
26223249	Han , R	2015	Coal workers ' pneumoconiosis (CWP)	China	Asian	Case-control study	693	690	1383	IL17A	rs3748067 C/T	C/T	**0.813 (0.668-0.990)**	0.434
26223249	Han , R	2015	Coal workers ' pneumoconiosis (CWP)	China	Asian	Case-control study	693	689	1382	IL17A	rs4711998 A/G	A/G	0.992 (0.841-1.170)	
26223249	Han , R	2015	Coal workers ' pneumoconiosis (CWP)	China	Asian	Case-control study	693	690	1383	IL17A	rs8193036 C/T	C/T	**0.811 (0.690-0.953)**	0.173
26337284	Yue, Y. X	2016	Myasthenia gravis (MG)	China	Asian	Case-control study	480	487	967	IL17A	rs8193036 C/T	**C/T**	0.956 (0.785-1.165)	
26337284	Yue, Y. X	2016	Myasthenia gravis (MG)	China	Asian	Case-control study	480	487	967	IL17A	rs2275913 G/A	G/A	1.068 (0.891-1.278)	
26337284	Yue, Y. X	2016	Myasthenia gravis (MG)	China	Asian	Case-control study	480	487	967	IL17A	rs3748067 C/T	C/T	0.861 (0.680-1.092)	
26337284	Yue, Y. X	2016	Myasthenia gravis (MG)	China	Asian	Case-control study	480	487	967	IL17F	rs763780 T/C	T/C	0.974 (0.730-1.300)	
26347322	Batalla , A	2015	Psoriasis	Spain	Caucasian	Case-control study	580	567	1147	IL17E	rs79877597 C/A	C/A	1.183 (0.973-1.438)	
26367076	LBassuoni M.	2015	Chronic infections with HCV	Egypt	African	Case-control study	75	20	95	IL17A	rs2275913 G/A	G/A	1.313 (0.646-2.668)	
26400516	Romanowski , M	2015	Post-transplant diabetes mellitus (PTDM)	Poland	Caucasian	Case-control study	23	146	169	IL17A	rs2275913 G/A	G/A	0.803 (0.410-1.573)	
26400516	Romanowski , M	2015	Post-transplant diabetes mellitus (PTDM)	Poland	Caucasian	Case-control study	23	146	169	IL17F	rs11465553 C/T	C/T	1.288 (0.358-4.636)	
26400516	Romanowski , M	2015	Post-transplant diabetes mellitus (PTDM)	Poland	Caucasian	Case-control study	23	146	169	IL17F	rs2397084 T/C	T/C	1.302 (0.472-3.594)	
26400516	Romanowski , M	2015	Post-transplant diabetes mellitus (PTDM)	Poland	Caucasian	Case-control study	23	146	169	IL17F	rs763780 T/C	T/C	**5.325 (1.757-16.143)**	0.825
26890073	Zhou , Z	2016	Gout	China	Asian	Case-control study	1101	1239	2340	IL17A	rs2275913 G/A	G/A	**0.585 (0.515-0.665)**	0.000
26890073	Zhou , Z	2016	Gout	China	Asian	Case-control study	1101	1239	2340	IL17F	rs763780 T/C	T/C	1.050 (0.870-1.268)	
26890073	Zhou , Z	2016	Gout	China	Asian	Case-control study	1101	1239	2340	IL17RA	rs4819554 G/A	G/A	0.931 (0.828-1.046)	
27003425	Korytina , G. F	2016	Chronic obstructive pulmonary disease (COPD)	Russia	Tatars	Case-control study	425	457	882	IL17A	rs4711998 A/G	A/G	1.116 (0.913-1.365)	
27003425	Korytina , G. F	2016	Chronic obstructive pulmonary disease (COPD)	Russia	Tatars	Case-control study	425	457	882	IL17A	rs1974226 C/T	C/T	**1.253 (1.008-1.558)**	0.460
27021337	Xu, H	2016	Henoch–Schonlein purpura (HSP).	China	Asian	Case-control study	148	202	350	IL17A	rs2275913 G/A	G/A	**0.695 (0.514-0.940)**	0.363
27021337	Xu, H	2016	Henoch–Schonlein purpura (HSP).	China	Asian	Case-control study	148	202	350	IL17A	rs8193037 G/A	G/A	1.133 (0.709-1.810)	
27021337	Xu, H	2016	Henoch–Schonlein purpura (HSP).	China	Asian	Case-control study	148	202	350	IL17A	rs3819025 G/A	G/A	1.462 (0.990-2.157)	
27021337	Xu, H	2016	Henoch–Schonlein purpura (HSP).	China	Asian	Case-control study	148	202	350	IL17F	rs763780 T/C	T/C	0.888 (0.557-1.418)	
27021337	Xu, H	2016	Henoch–Schonlein purpura (HSP).	China	Asian	Case-control study	148	202	350	IL17F	rs9463772 C/T	C/T	1.115 (0.740-1.681)	
27155288	Rogo , L. D	2016	Influenza A/H3N2 virus infection	Iran	Caucasian	Case-control study	96	147	243	IL17A	rs2275913 G/A	G/A	**0.467 (0.317-0.690)**	0.063
27155288	Rogo , L. D	2016	Influenza like illness (ILI)	Iran	Caucasian	Case-control study	114	147	261	IL17A	rs2275913 G/A	G/A	**0.574 (0.400-0.822)**	0.183
27155445	Erkol İnal, E	2016	Ankylosing spondylitis (AS)	Turkey	Caucasian	Case-control study	101	106	207	IL17F	rs763780 T/C	T/C	**2.276 (1.161-4.461)**	0.738
27155445	Erkol İnal, E	2016	Ankylosing spondylitis (AS)	Turkey	Caucasian	Case-control study	101	106	207	IL17F	rs2397084 T/C	T/C	0.674 (0.347-1.309)	
27169372	Pawlik , A	2016	Rheumatoid arthritis (RA)	Poland	Caucasian	Case-control study	422	337	759	IL17F	rs11465553 C/T	C/T	0.983 (0.622-1.554)	
27312555	Liu, S	2016	Immune thrombocytopenia (ITP)	China	Asian	Case-control study	146	137	283	IL17A	rs2275913 G/A	G/A	1.314 (0.942-1.833)	
27339100	Wang , M	2016	Tuberculosis (TB)	China	Asian	Case-control study	1601	1526	3127	IL17A	rs3819024 A/G	A/G	0.997 (0.902-1.102)	
27561723	Resende , E. P	2017	Rhinitis	Portugal	Caucasian	Case-control study	149	192	341	IL17A	rs2275913 G/A	G/A	0.974 (0.699-1.357)	
27577072	Zhou, F	2016	Gastric Cancer (GC)	China	Asian	Case-control study	1121	1216	2337	IL17A	rs1974226 C/T	C/T	0.999 (0.802-1.246)	
27591988	Białecka , M	2016	Psoriasis	Poland	Caucasian	Case-control study	407	205	612	IL17F	rs11465553 C/T	C/T	1.353 (0.764-2.395)	
27595159	Kharwar , N. K	2017	Guillain-Barré syndrome (GBS)	India	Other	Case-control study	80	75	155	IL17F	rs763780 T/C	T/C	0.964 (0.599-1.553)	
28224332	Tian, J	2017	Necrotizing enterocolitis (NEC)	China	Asian	Case-control study	102	120	222	IL17A	rs2275913 G/A	G/A	0.995 (0.683-1.450)	
28224332	Tian, J	2017	Necrotizing enterocolitis (NEC)	China	Asian	Case-control study	102	120	222	IL17F	rs763780 T/C	T/C	**1.824 (1.062-3.132)**	0.700
28362993	Si, F. Z	2017	Laryngeal cancer	China	Asian	Case-control study	325	325	650	IL17A	rs2275913 G/A	G/A	**1.567 (1.245-1.927)**	0.001
28362993	Si, F. Z	2017	Laryngeal cancer	China	Asian	Case-control study	325	325	650	IL17A	rs3748067 C/T	C/T	**0.665 (0.454-0.974)**	0.581
28362993	Si, F. Z	2017	Laryngeal cancer	China	Asian	Case-control study	325	325	650	IL17F	rs763780 T/C	T/C	**0.676 (0.476-0.959)**	0.502
28410448	Neco, Hvpc	2017	HTLV-1-associated myelopathy/tropical spastic paraparesis (HAM/TSP)	Brazil	Mixed	Case-control study	29	87	116	IL17A	rs2275913 G/A	G/A	1.548 (0.767-3.123)	
28470012	Reis, P. G	2017	Chagas disease (CD)	Brazil	Mixed	Case-control study	212	150	362	IL17A	rs2275913 G/A	G/A	**1.522 (1.078-2.149)**	0.409
28470012	Reis, P. G	2017	Chagas disease (CD)	Brazil	Mixed	Case-control study	212	150	362	IL17F	rs763780 T/C	T/C	1.193 (0.652-2.182)	
28470012	Reis, P. G	2017	Chagas disease (CD)	Brazil	Mixed	Case-control study	48	150	198	IL17A	rs2275913 G/A	G/A	1.205 (0.704-2.063)	
28470012	Reis, P. G	2017	Chagas disease (CD)	Brazil	Mixed	Case-control study	48	150	198	IL17F	rs763780 T/C	T/C	1.044 (0.402-2.711)	
28481151	Hassani , E	2017	Silicosis	Iran	Kurdish	Case-control study	48	62	110	IL17A	rs4711998 A/G	A/G	1.194 (0.680-2.097)	
28731539	Liehu M, M	2017	Bacillus Calmette-Guérin (BCG) osteitis	Finland	Caucasian	Case-control study	132	405	537	IL17A	rs2275913 G/A	G/A	**1.447 (1.095-1.912)**	0.096
28741807	Gueiros , L. A	2018	Oral lichen planus (OLP)	Brazil	Mixed	Case-control study	83	99	182	IL17A	rs2275913 G/A	G/A	**2.416 (1.514-3.856)**	0.153
28786198	Kasamatsu , T	2018	Multiple myeloma (MM)	Japan	Asian	Case-control study	120	201	321	IL17A	rs2275913 G/A	G/A	0.981 (0.707-1.360)	
28873198	Niu, A. Q	2017	Cervical cancer	China	Asian	Case-control study	185	370	555	IL17A	rs2275913 G/A	G/A	1.142 (0.873-1.494)	
28873198	Niu, A. Q	2017	Cervical cancer	China	Asian	Case-control study	185	370	555	IL17A	rs3748067 C/T	C/T	**2.313 (1.558-3.436)**	0.038
29400002	Vahabi , S	2018	Periodontitis	Iran	Caucasian	Case-control study	99	75	174	IL17F	rs2397084 T/C	T/C	1.420 (0.658-3.062)	
29530464	Tang, H	2018	Viral myocarditis (VMC)	China	Asian	Case-control study	236	259	495	IL17A	rs2275913 G/A	G/A	**1.930 (1.429-2.607)**	0.007
29530464	Tang, H	2018	Viral myocarditis (VMC)	China	Asian	Case-control study	236	259	495	IL17A	rs3819025 G/A	G/A	0.972 (0.699-1.351)	
29530464	Tang, H	2018	Viral myocarditis (VMC)	China	Asian	Case-control study	236	259	495	IL17A	rs3748067 C/T	C/T	0.884 (0.637-1.227)	
29584788	Dhaouadi , T	2018	Rheumatoid arthritis (RA)	Tunisia	Caucasian	Case-control study	115	91	206	IL17RC	rs708567 C/T	C/T	1.001 (0.670-1.497)	
29689450	Bedoui , S. A	2018	Colorectal cance (CRC)	Tunisia	Caucasian	Case-control study	294	268	562	IL17A	rs3819024 A/G	A/G	1.041 (0.784-1.383)	
29689450	Bedoui , S. A	2018	Colorectal cance (CRC)	Tunisia	Caucasian	Case-control study	294	268	562	IL17A	rs3819025 G/A	G/A	1.053 (0.763-1.453)	
29689450	Bedoui , S. A	2018	Colorectal cance (CRC)	Tunisia	Caucasian	Case-control study	294	268	562	IL17A	rs10484879 C/A	C/A	**2.036 (1.418-2.925)**	0.044
29689450	Bedoui , S. A	2018	Colorectal cance (CRC)	Tunisia	Caucasian	Case-control study	294	268	562	IL17A	rs7747909 G/A	G/A	0.798 (0.584-1.089)	
29689450	Bedoui , S. A	2018	Colorectal cance (CRC)	Tunisia	Caucasian	Case-control study	294	268	562	IL17A	rs3748067 C/T	C/T	1.053 (0.763-1.453)	
29704988	Dai, Y	2018	Chronic obstructive pulmonary disease (COPD)	China	Asian	Case-control study	152	201	353	IL17A	rs3819024 A/G	A/G	0.919 (0.675-1.251)	
29704988	Dai, Y	2018	Chronic obstructive pulmonary disease (COPD)	China	Asian	Case-control study	152	201	353	IL17A	rs3748067 C/T	C/T	**1.599 (1.077-2.373)**	0.500
29704988	Dai, Y	2018	Chronic obstructive pulmonary disease (COPD)	China	Asian	Case-control study	152	201	353	IL17F	rs763780 T/C	T/C	**1.960 (1.288-2.981)**	0.230
29733805	Akbulut , U. E	2019	Non-alcoholic fatty liver disease (NAFLD)	Turkey	Caucasian	Case-control study	101	85	186	IL17A	rs2275913 G/A	G/A	**1.928 (1.269-2.929)**	0.249
29764467	Wang , P	2018	Ossification of the posterior longitudinal ligament (OPLL)	China	Asian	Case-control study	100	100	200	IL17RC	rs199772854 C/A	C/A	**6.319 (1.396-28.611)**	0.911
29764467	Wang , P	2018	Ossification of the posterior longitudinal ligament (OPLL)	China	Asian	Case-control study	100	100	200	IL17RC	rs76999397 G/A	G/A	**4.714 (1.006-22.102)**	0.927
29764467	Wang , P	2018	Ossification of the posterior longitudinal ligament (OPLL)	China	Asian	Case-control study	100	100	200	IL17RC	rs189013166 G/A	G/A	**8.292 (1.027-66.923)**	0.943
29985710	oomarimuthu , I	2018	Rheumatic heart disease (RHD)	India	Other	Case-control study	99	126	225	IL17A	rs2275913 G/A	G/A	1.349 (0.921-1.978)	
29985710	oomarimuthu , I	2018	Rheumatic heart disease (RHD)	India	Other	Case-control study	99	126	225	IL17F	rs763780 T/C	T/C	1.020 (0.514-2.024)	
30138899	Subbanna , M	2018	Schizophrenia	India	Other	Case-control study	221	223	444	IL17A	rs2275913 G/A	G/A	0.878 (0.668-1.153)	
30196825	Debnath , M	2018	Guillain-Barré syndrome (GBS)	India	Other	Case-control study	220	205	425	IL17A	rs2275913 G/A	G/A	0.791 (0.599-1.045)	
30398030	Montúfar Robles , I	2019	Systemic lupus erythematosus (SLE)	Mexico	Mixed	Case-control study	367	499	866	IL17A	rs8193037 G/A	G/A	1.275 (0.904-1.798)	
30398030	Montúfar Robles , I	2019	Systemic lupus erythematosus (SLE)	Mexico	Mixed	Case-control study	367	499	866	IL17A	rs3819024 A/G	A/G	1.010 (0.775-1.317)	
30446888	Aguín, N	2020	Prostate cancer (PCa)	Spain	Caucasian	Case-control study	241	192	433	IL17A	rs2275913 G/A	G/A	0.978 (0.735-1.301)	

30655311	Xie, M	2019	Acute respiratory distress syndrome (ARDS)	China	Asian	Case-control study	210	210	420	IL17F	rs763780 T/C	T/C	1.444 (0.885-2.356)	
30655311	Xie, M	2019	Acute respiratory distress syndrome (ARDS)	China	Asian	Case-control study	210	210	420	IL17A	rs2275913 G/A	G/A	**0.223 (0.161-0.307)**	0.000
30655311	Xie, M	2019	Acute respiratory distress syndrome (ARDS)	China	Asian	Case-control study	210	210	420	IL17A	rs8193036 C/T	C/T	**2.230 (1.648-3.018)**	0.001
30848577	Hesampour, F	2019	Polycystic ovary syndrome (PCOS)	Iran	Caucasian	Case-control study	150	150	300	IL17A	rs2275913 G/A	G/A	**0.545 (0.355-0.839)**	0.381
31144366	Bafrani, H. H	2019	Osteoarthritis (OA)	Iran	Caucasian	Case-control study	127	127	254	IL17F	rs2397084 T/C	T/C	0.881 (0.439-1.770)	
31171891	Yu, Z. Q	2019	Intestinal tuberculosis (ITB)	China	Asian	Case-control study	133	500	633	IL17A	rs8193036 C/T	C/T	1.049 (0.777-1.416)	
31196204	Keshavarz, M	2019	Influenza A (H1N1)	Iran	Caucasian	Case-control study	50	96	146	IL17A	rs2275913 G/A	G/A	**0.474 (0.272-0.829)**	0.592
31196204	Keshavarz, M	2019	Influenza B	Iran	Caucasian	Case-control study	30	96	126	IL17A	rs2275913 G/A	G/A	0.690 (0.359-1.326)	
31198448	Elsissy, M	2019	Acute Myeloid Leukaemia (AML)	Egypt	African	Case-control study	100	100	200	IL17A	rs2275913 G/A	G/A	0.729 (0.486-1.091)	
31198448	Elsissy, M	2019	Acute Myeloid Leukaemia (AML)	Egypt	African	Case-control study	100	100	200	IL17F	rs763780 T/C	T/C	**0.505 (0.317-0.805)**	0.390
31230447	Ramzi, M	2019	Torque teno virus infection	Iran	Caucasian	Cross-sectional study	27	45	72	IL17A	rs2275913 G/A	G/A	0.844 (0.378-1.885)	
31280929	Carnt, N. A	2019	Microbial keratitis (MK)	Australia and UK	Caucasian	Case-control study	88	185	273	IL17A	rs2275913 G/A	G/A	1.041 (0.708-1.529)	
31280929	Carnt, N. A	2019	Microbial keratitis (MK)	Australia and UK	Caucasian	Case-control study	88	185	273	IL17F	rs2397084 T/C	T/C	1.248 (0.714-2.182)	
31344596	Gonçalves de Albuquerque, S. D. C	2019	Cutaneous leishmaniasis (CL)	Brazil	Mixed	Case-control study	172	173	345	IL17A	rs2275913 G/A	G/A	1.245 (0.896-1.730)	
31845023	Dimberg, J	2020	Acute appendicitis (AA)	Sweden	Caucasian	Case-control study	100	243	343	IL17A	rs2275913 G/A	G/A	**1.969 (1.408-2.754)**	0.025
31864094	Wang, W	2020	Tuberculosis (TB)	China	Asian	Case-control study	926	948	1874	IL17A	rs8193039 G/A	G/A	0.957 (0.772-1.186)	
31864094	Wang, W	2020	Tuberculosis (TB)	China	Asian	Case-control study	926	948	1874	IL17A	rs3804513 A/T	A/T	1.034 (0.807-1.324)	
31864094	Wang, W	2020	Tuberculosis (TB)	China	Asian	Case-control study	926	948	1874	IL17A	rs3804513 A/G	A/G	0.989 (0.850-1.151)	
32138639	Rahimzadeh, M	2020	Acute kidney injury (AKI)	Iran	Caucasian	Case-control study	65	70	135	IL17A	rs2275913 G/A	G/A	0.913 (0.531-1.572)	
32138639	Rahimzadeh, M	2020	Acute kidney injury (AKI)	Iran	Caucasian	Case-control study	65	70	135	IL17A	rs3819024 A/G	A/G	0.659 (0.402-1.082)	
32242803	López Mejías, R	2020	Immunoglobulin-A vasculitis (IgAV)	Spain	Caucasian	Case-control study	360	1003	1363	IL17A	rs4711998 A/G	A/G	0.979 (0.808-1.186)	
32242803	López Mejías, R	2020	Immunoglobulin-A vasculitis (IgAV)	Spain	Caucasian	Case-control study	360	1003	1363	IL17A	rs8193036 C/T	C/T	0.883 (0.722-1.078)	
32242803	López Mejías, R	2020	Immunoglobulin-A vasculitis (IgAV)	Spain	Caucasian	Case-control study	360	1003	1363	IL17A	rs3819024 A/G	A/G	1.109 (0.926-1.327)	
32242803	López Mejías, R	2020	Immunoglobulin-A vasculitis (IgAV)	Spain	Caucasian	Case-control study	360	1003	1363	IL17A	rs2275913 G/A	G/A	1.034 (0.863-1.238)	
32242803	López Mejías, R	2020	Immunoglobulin-A vasculitis (IgAV)	Spain	Caucasian	Case-control study	360	1003	1363	IL17A	rs7747909 G/A	G/A	0.963 (0.794-1.167)	
32294519	Tayefinasrabadi, H	2020	Hepatitis B Virus (HBV)	Iran	Caucasian	Case-control study	199	172	371	IL17F	rs2397084 T/C	T/C	1.009 (0.630-1.616)	
32294519	Tayefinasrabadi, H	2020	Hepatitis B Virus (HBV)	Iran	Caucasian	Case-control study	199	172	371	IL17A	rs10484879 C/A	C/A	**0.690 (0.490-0.970)**	0.518
32355229	Tabatabaei Panah, P. S	2020	Alopecia areata	Iran	Caucasian	Case-control study	60	60	120	IL17A	rs2275913 G/A	G/A	0.915 (0.399-2.095)	
32380889	Elkoumi, M. A	2020	Systemic lupus erythematosus (SLE)	Egypt	African	Case-control study	320	320	640	IL17A	rs3748067 C/T	C/T	0.891 (0.704-1.128)	
32522475	Chen, S	2020	Anti-E alloantibody production	China	Asian	Case-control study	95	186	281	IL17F	rs763780 T/C	T/C	**3.077 (1.663-5.695)**	0.372
32537881	Zhao, T	2020	Osteoarthritis (OA)	China	Asian	Case-control study	796	1854	2650	IL17F	rs12201582 C/A	C/A	**0.225 (0.155-0.327)**	0.000
32537881	Zhao, T	2020	Osteoarthritis (OA)	China	Asian	Case-control study	796	1854	2650	IL17F	rs12203736 G/A	G/A	0.979 (0.869-1.103)	
32537881	Zhao, T	2020	Osteoarthritis (OA)	China	Asian	Case-control study	796	1854	2650	IL17F	rs722323 G/A	G/A	**1.589 (1.392-1.814)**	0.000
32537881	Zhao, T	2020	Osteoarthritis (OA)	China	Asian	Case-control study	796	1854	2650	IL17F	rs117507014 T/C	T/C	0.966 (0.820-1.137)	
32537881	Zhao, T	2020	Osteoarthritis (OA)	China	Asian	Case-control study	796	1854	2650	IL17F	rs1266828 G/A	G/A	0.961 (0.821-1.125)	
32537881	Zhao, T	2020	Osteoarthritis (OA)	China	Asian	Case-control study	796	1854	2650	IL17F	rs2294835 A/G	A/G	1.024 (0.903-1.161)	
32654355	Lauhkonen, E	2021	Bronchiolitis	Finland	Caucasian	Case-control study	266	99	365	IL17RA	rs4819554 G/A	G/A	0.775 (0.502-1.196)	
32946631	Korppi, M	2021	Bacillus Calmette-Guérin (BCG) osteitis	Finland	Caucasian	Case-control study	132	99	231	IL17F	rs763780 T/C	T/C	1.109 (0.560-2.198)	
32946631	Korppi, M	2021	Bacillus Calmette-Guérin (BCG) osteitis	Finland	Caucasian	Case-control study	132	99	231	IL17F	rs11465553 C/T	C/T	1.842 (0.789-4.299)	
32946631	Korppi, M	2021	Bacillus Calmette-Guérin (BCG) osteitis	Finland	Caucasian	Case-control study	132	99	231	IL17F	rs7741835 C/T	C/T	1.061 (0.660-1.706)	
32950026	Coto, E	2020	Coronary artery disease (CAD)	Spain	Caucasian	Case-control study	450	390	840	IL17RA	rs4819554 G/A	G/A	0.857 (0.682-1.078)	
32969586	Nada, H	2021	Chronic spontaneous urticaria (CSU)	Egypt	African	Case-control study	70	30	100	IL17RA	rs4819554 G/A	G/A	**0.533 (0.289-0.982)**	0.778
32969586	Nada, H	2021	Chronic spontaneous urticaria (CSU)	Egypt	African	Case-control study	70	30	100	IL17RA	rs879577 C/T	C/T	**0.486 (0.262-0.899)**	0.722
33340282	Tabatabaei Panah, P. S	2020	Bullous pemphigoid (BP)	Iran	Caucasian	Case-control study	40	40	80	IL17F	rs2397084 T/C	T/C	1.370 (0.453-4.146)	
33340282	Tabatabaei Panah, P. S	2020	Bullous pemphigoid (BP)	Iran	Caucasian	Case-control study	40	40	80	IL17F	rs763780 T/C	T/C	2.111 (0.609-7.315)	
33340282	Tabatabaei Panah, P. S	2020	Bullous pemphigoid (BP)	Iran	Caucasian	Case-control study	40	40	80	IL17RA	rs2229151 G/A	G/A	1.541 (0.418-5.681)	
33351682	Xiang, H	2020	Recurrent aphthous ulcer (RAU)	China	Asian	Case-control study	125	116	241	IL17A	rs2275913 G/A	G/A	**1.783 (1.242-2.560)**	0.158
33351682	Xiang, H	2020	Recurrent aphthous ulcer (RAU)	China	Asian	Case-control study	125	116	241	IL17F	rs763780 T/C	T/C	**1.969 (1.257-3.083)**	0.332
33364812	Li, W	2020	Vulvovaginal candidiasis (VVC)	China	Asian	Case-control study	213	326	539	IL17A	rs2275913 G/A	G/A	**1.406 (1.087-1.818)**	0.205
33364812	Li, W	2020	Vulvovaginal candidiasis (VVC)	China	Asian	Case-control study	213	326	539	IL17F	rs763780 T/C	T/C	**1.449 (1.083-1.928)**	0.259
33818451	Khatonier, R	2020	Kala-azar	India	Other	Case-control study	76	133	209	IL17A	rs2275913 G/A	G/A	1.205 (0.766-1.896)	
33818451	Khatonier, R	2020	Kala-azar	India	Other	Case-control study	76	133	209	IL17A	rs8193036 C/T	C/T	**0.609 (0.389-0.953)**	0.622
34554306	Hristova, M	2021	Systemic lupus erythematosus (SLE)	Bulgaria	Caucasian	Case-control study	59	95	154	IL17RC	rs708567 C/T	C/T	0.837 (0.528-1.327)	
34554386	Gomes, I. B	2021	Keratoconus (KC)	Brazil	Mixed	Case-control study	35	61	96	IL17A	rs2275913 G/A	G/A	1.110 (0.595-2.071)	
34554386	Gomes, I. B	2021	Keratoconus (KC)	Brazil	Mixed	Case-control study	35	61	96	IL17F	rs763780 T/C	T/C	0.657 (0.357-1.208)	

Note: OR, odds ratio; A, adenine; T, thymine; G, guanine; C, cytosine;

[a]Major alleles/minor alleles.

[b]The prior probability of FPRP is 0.05, and the FPRP level of noteworthiness is 0.20.

Supplementary Table 6. Analyses of expression quantitative trait locus (eQTL) in significant variants associated with risk of cancer and non-neoplastic diseases

Variant	Gene	Tissue	*P* value	Effect Size
rs2275913	GSTA8P, IL17A	Testis	8.3×10^{-5}	0.17
rs763780	MCM3,IL17F	Muscle	1.0×10^{-4}	0.2
rs8193037	MCM3,IL17F	Esophagus	1.1×10^{-5}	0.43
rs1889570	IL17F	NA	NA	NA

Data source: Genotype-Tissue Expression (GTEx) Project. NA: Not applicable.

Supplementary Table 7. Correlations (r^2) among the four variants showing strong evidence using data from the 1000 Genomes Project.

**European**				
Variants	rs763780	rs1889570	rs2275913	rs8193037
rs763780	1			
rs1889570	0.0035	1		
rs2275913	0.0094	0.303	1	
rs8193037	0.0010	0.0123	0.0099	1
**Asian**				
Variants	rs763780	rs1889570	rs2275913	rs8193037
rs763780	1			
rs1889570	0.1091	1		
rs2275913	0.0011	0.2131	1	
rs8193037	0.0175	0.0369	0.1	1
**African**				
Variants	rs763780	rs1889570	rs2275913	rs8193037
rs763780	1			
rs1889570	0.0186	1		
rs2275913	0.0049	0.0303	1	
rs8193037	0.0004	0.0009	0.0002	1

moderate   weak   uncorrelated

Data source: ldlink.nci.nih.gov.
Asians, European, and African were selected from five major population categories provided by the databases.
